# Radiographic Response Assessment Strategies for Early-Phase Brain Trials in Complex Tumor Types and Drug Combinations: from Digital “Flipbooks” to Control Systems Theory

**DOI:** 10.1007/s13311-022-01241-8

**Published:** 2022-04-22

**Authors:** Benjamin M. Ellingson, Victor A. Levin, Timothy F. Cloughesy

**Affiliations:** 1 UCLA Brain Tumor Imaging Laboratory, Center for Computer Vision and Imaging Biomarkers, David Geffen School of Medicine, University of California Los Angeles, Los Angeles, CA USA; 2 Department of Radiologic Sciences, David Geffen School of Medicine, University of California Los Angeles, Los Angeles, CA USA; 3 David Geffen School of Medicine, UCLA Brain Tumor Program, University of California Los Angeles, Los Angeles, CA USA; 4 Department of Neuro-Oncology, The University of Texas MD Anderson Cancer Center, Houston, TX USA; 5 Department of Neurosurgery, UCSF Medical School, San Francisco, CA USA; 6 Department of Neurology, David Geffen School of Medicine, University of California Los Angeles, Los Angeles, CA USA

**Keywords:** Digital flipbooks, RANO, Brain tumors, Imaging response, Early-phase trials, Control systems theory

## Abstract

There is an urgent need for drug development in brain tumors. While current radiographic response assessment provides instructions for identifying large treatment effects in simple high- and low-grade gliomas, there remains a void of strategies to evaluate complex or difficult to measure tumors or tumors of mixed grade with enhancing and non-enhancing components. Furthermore, most patients exhibit some period of alteration in tumor growth after starting a new therapy, but simple response categorization (e.g., stable disease, progressive disease) fails to provide any meaningful insight into the depth or degree of potential “subclinical” therapeutic response. We propose a creative solution to these issues based on a tiered strategy meant to increase confidence in identifying therapeutic effects even in the most challenging tumor types, while also providing a framework for complex evaluation of combination and sequential treatment schemes. Specifically, we demonstrate the utility of digital “flipbooks” to quickly identify subtle changes in complex tumors. We show how a modified Levin criteria can be used to quantify the degree of visual changes, while establishing estimates of the association between tumor volume and visual inspection. Lastly, we introduce the concept of quantifying therapeutic response using control systems theory. We propose measuring changes in volume (proportional), the area under the volume vs. time curve (integral) and changes in growth rates (derivative) to utilize a “PID” controller model of single or combination therapeutic activity.

**Supplementary Information:**

The online version contains supplementary material available at 10.1007/s13311-022-01241-8.

## Role of Imaging in Response Assessment and Clinical Care in Neuro-Oncology

A recent statistical report of primary and other central nervous system (CNS) tumors compiled by the Central Brain Tumor Registry of the United States (CBTRUS) estimated 83,830 new cases of primary malignant and non-malignant brain, and other CNS tumors would be expected to be diagnosed in the USA in 2020 [1]. This includes an expected 24,970 primary malignant and 58,860 primary non-malignant tumors with glioblastoma (GBM) being 49% of malignant tumors and meningiomas being the most common primary non-malignant tumor at 54% of non-malignant tumors. They found the incidence rate for GBM was 3.23 per 100,000 population, followed by malignant glioma not otherwise specified at 0.51 per 100,000, and diffuse astrocytoma was 0.45 per 100,000 population.

Unfortunately, the survival outcome for high-grade infiltrative glioma patients, such as those with GBM, is quite dire with a median overall survival (OS) of 14–16 months [2, 3] and 8–10% of patients surviving beyond 5 years after diagnosis [3, 4]. Currently, the standard of care for newly diagnosed GBM patients consists of maximum safe surgical resection, followed by radiotherapy plus concomitant and adjuvant temozolomide. Even with the introduction of temozolomide and its universal acceptance as the adjuvant treatment of choice for newly diagnosed GBM patients [2], the dismal prognosis has not changed substantially in the past 30 years. In addition, at recurrence, few therapeutic options exist. A careful review of the clinical trials from 2006 to 2012 involving recurrent or progressive GBM found a minority of patients eligible for a second surgery or reirradiation. Most patients require further chemotherapy with drugs different than temozolomide. Sadly, patients failing adjuvant temozolomide experience progression-free survival (PFS) rates at 6 months of 20–30% with nitrosoureas, temozolomide re-challenge, or bevacizumab [5]. There has been an urgent and unmet need for new drugs to treat high-grade gliomas for more than three decades [6, 7].

The reasons for this failure of therapeutic innovation have many causes not the least of which is pharmaceutical industry aversion for high risk, low reward drug development that has plagued drug discovery, and development for high-grade infiltrative gliomas such as GBM. This is also reflected in the current regulatory environment that considers OS as the gold standard for effective therapy and, in most cases, the only standard. Unfortunately, OS may not directly reflect the impact of specific treatment regimens because of potential confounding effects like prognostic factors and potential subsequent therapies. This is especially relevant when studying new therapeutics, where even subtle evidence of treatment effects may be insightful. Therefore, we will argue that non-invasive radiographic endpoints can be an effective tool to study tumor behavior in the context of drug development for brain tumors.

Currently, T1-weighted MR images used with the addition of T1-shortening gadolinium-based contrast agents are the standard for radiographic diagnosis and response assessment of high-grade, malignant brain tumors. Areas of blood–brain barrier breakdown allow diffusion of the contrast agent out of vasculature into the extravascular extracellular space [8], resulting in hyperintensity on T1-weighted images in the most aggressive portion of the tumor [9, 10]. Cytoarchitectural disruption from infiltrating tumor and vasogenic edema both cause increase in tissue T2, resulting in subsequent hyperintensity on T2-weighted turbo spin echo and T2-weighted fluid-attenuated inversion recovery (FLAIR) MRI [11–14]. Thus, the combination of contrast-enhanced T1-weighted images and T2-weighted FLAIR (or turbo spin echo) is often used to characterize enhancing and non-enhancing disease in both high-grade (HGGs) and low-grade gliomas (LGGs).

## Challenges with Complex and Mixed Tumor Types

**Fig. 1:**
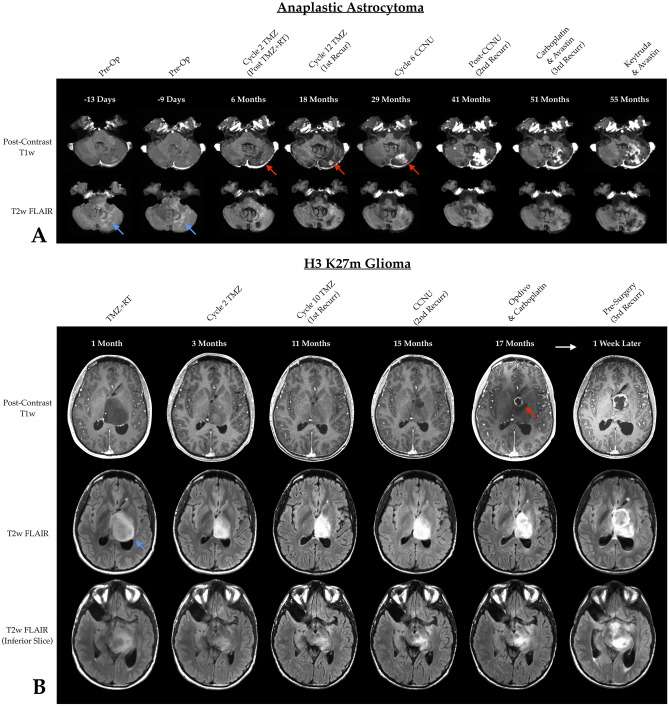
**A** A 64-year-old female patient with a cerebellar grade 3 anaplastic astrocytoma (AA) showing serial post-contrast T1-weighted (top row) and T2-weighted FLAIR images (bottom row). The lesion started out non-enhancing and only (blue arrows), but over time developed both enhancing (red arrows) and non-enhancing tumor growth. **B** A 22-year-old male patient with an H3 K27m mutation showing serial post-contrast T1-weighted (top row) and T2-weighted FLAIR images (middle row). Also showed is T2-weighted FLAIR of an inferior slice demonstrating tumor growth. Similar to (A), this patient demonstrated early non-enhancing tumor (blue arrows) that later developed enhancing disease (red arrow), both of which continue to grow

**Fig. 2:**
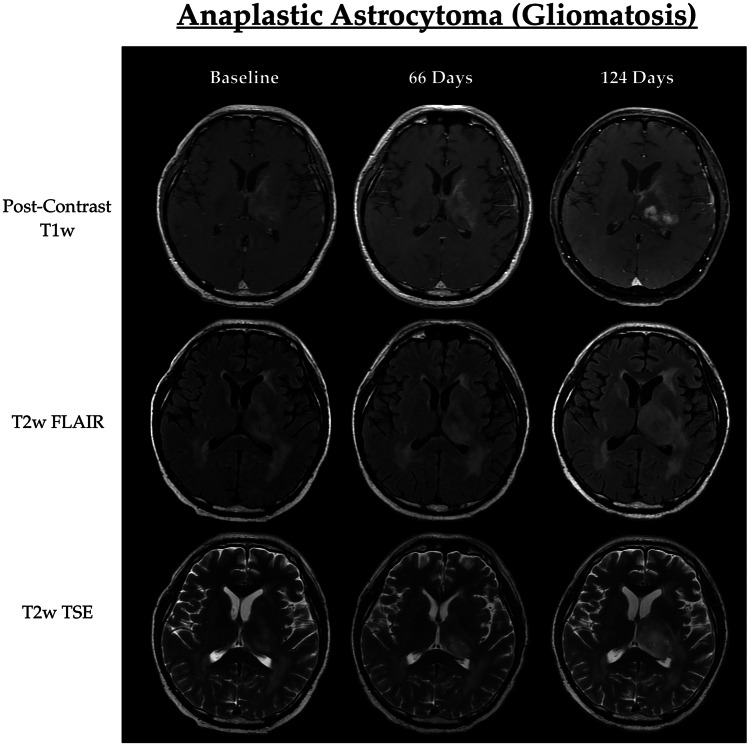
A 48-year-old patient with an anaplastic astrocytoma exhibiting difficult-to-measure gliomatosis-like features

While the standard HGG RANO criteria [15, 16] is useful for response criteria for high-grade gliomas exhibiting contrast enhancement and the LGG RANO criteria [17] is useful for radiographic response assessment in non-enhancing LGGs, there are no guidelines for “mixed”-grade gliomas, “molecular” (IDH wild type) glioblastoma, or patients that may not have measurable contrast enhancement at diagnosis or baseline but may develop enhancement over time (Fig. 1A). Similarly, there is no straightforward way of evaluating complex and unique genetic tumors that have a mixture of enhancing and non-enhancing tumor throughout their clinical history such as those exhibited by H3K27m midline gliomas (Fig. 1B). Additionally, radiographic response criteria strongly depend on the ability and accuracy of lesion measurements, whether it is bidirectional or volumetric. Therefore, the current response assessment tools lack guidance on how to study difficult to measure tumors, including those exhibiting “gliomatosis”-like features (Fig. 2).

## Challenges Assessing Drug Combinations in Gliomas

Although most contemporary brain tumor clinical trials involve the evaluation of single therapeutic agents, strategies regarding how to best evaluate these agents and combination therapies are lacking. At times, even single-agent therapy dose and frequency of administration are defined by drug experience against extracranial cancers, preclinical toxicology models, or a limited phase I study. A precise understanding of the temporal and dose dependence of a single drug would be of great help in the design of therapeutic combinations. In theory, single agents with some measurable therapeutic or clinical “activity” might be candidates for exploration of combination therapies. However, the current RANO framework does not appreciate this potential nuance, ascribing “stable disease” to many agents that are seemingly below the radiographic threshold for “response.” Even if single agents are effective at shrinking the tumor, the synergistic effects of an additional agent are not easily quantified using the current RANO criteria beyond incremental increases in the objective response rate (ORR).

## Tiered Approach to Radiographic Response Assessment

**Fig. 3:**
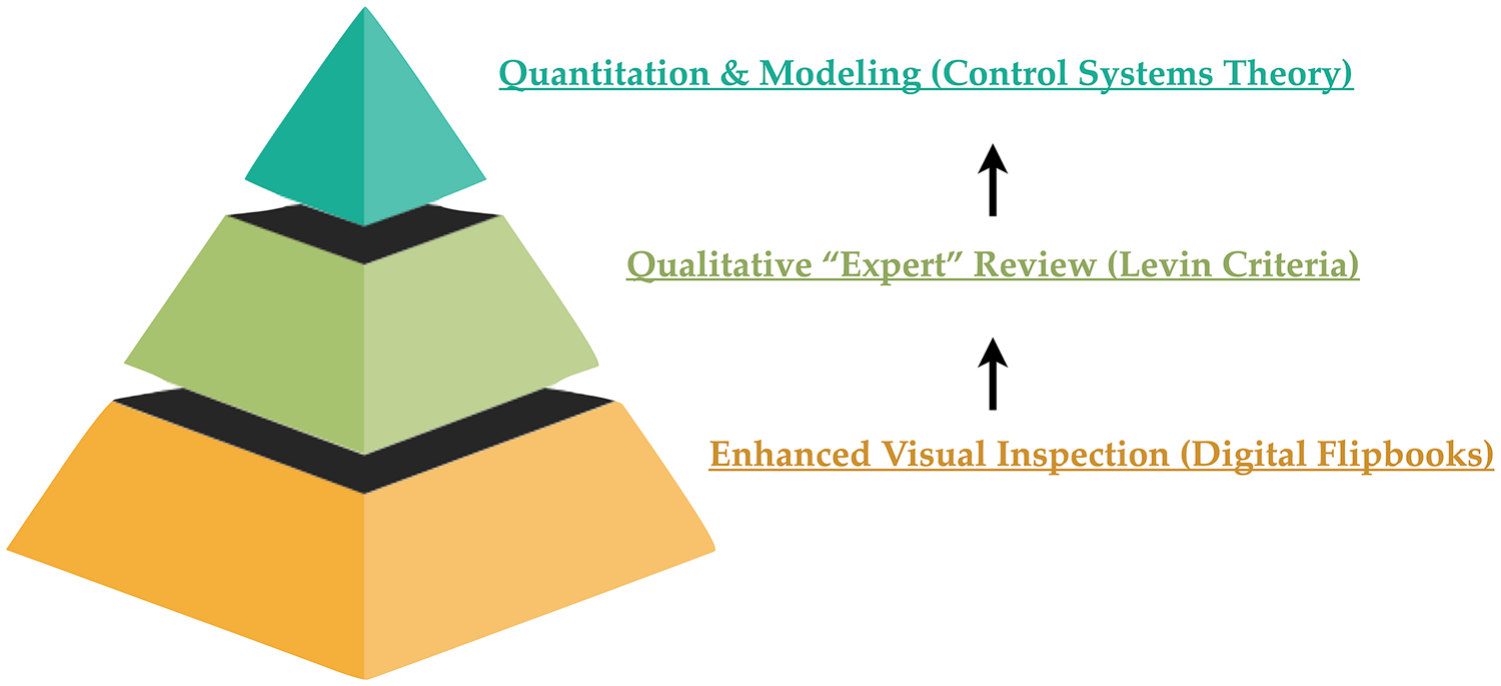
Tiered approach to radiographic assessment for any type of brain tumor. At the foundation, all patients can undergo an enhancing visual inspection of serial changes after image standardization (i.e., using BTIP [21]) and co-registration/alignment (e.g., digital flipbooks) have been performed. Even in easy-to-measure tumors, confirmation of quantitative changes using flipbooks can provide additional confidence in the accuracy of the measurements. Once enhanced visual inspection is performed, a qualitative “expert” review can be performed on enhancing tumor (using post-contrast T1-weighted images) and/or non-enhancing tumor (using T2/T2-weighted FLAIR) using modifications to the Levin criteria [27]. Readers can rate whether tumors are stable, growing, or shrinking, and to what level of confidence they have in this possible change (e.g. “possibly,” “definitely,” or “significantly” larger). If the lesion can be quantified, then more sophisticated modeling techniques can be implemented, including the use of control systems theory or mathematical modeling. Note that even semi-quantitative scoring can be used as a signal over time, but with less granularity than quantitative volume measurements

At a fundamental level, any patient that has a response to a therapeutic agent should have images that convincingly demonstrate this effect. In tumors that are non-measurable, whether to complex, small, or difficult to measure, a meaningful therapeutic effect should at a minimum be intuitively obvious to even the most casual observer. Tumors that *are* measurable *and* have a meaningful radiographic response should also be able to provide visual evidence to corroborate this effect. Thus, one may consider utilizing a “tiered” approach to evaluate *any* brain tumor patient that is on a study drug by starting with a visual inspection of the radiographic data before and during therapy to get the gestalt of the effect, then adding various levels of quantitation and sophistication to enhance and build confidence around this observation (Fig. 3).

## Enhanced Visual Inspection Using Digital “Flipbooks”

**Fig. 4:**
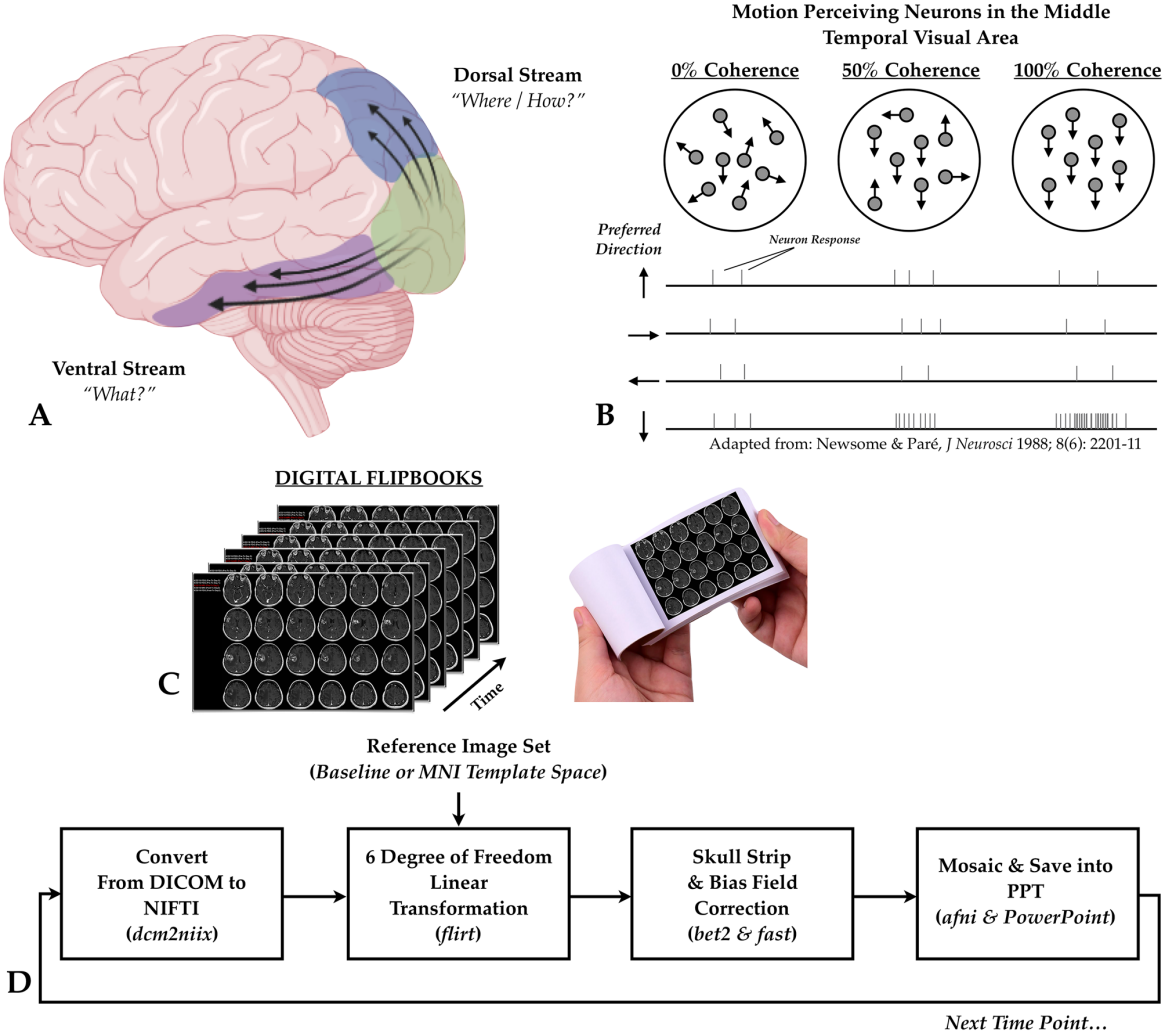
**A** The dorsal and ventral streams of visual motion sensation [18–20]. **B** Motion perceiving neurons in the middle temporal visual area, showing increased action potential firing rate in orientation-dependent neurons when the motion is coherent and in the same direction (adapted from [20]). **C** Digital “flipbooks” created to exploit these neurons by registering/aligning images over time, allowing the user to flip through each time point on separate pages. **D** Process for creating digital flipbooks starts with conversion of DICOM to NIFTI format. Then, images are registered to a reference image set, either the patient’s own baseline or some template dataset (e.g., MNI152) using a 6 degrees of freedom linear transformation. Following alignment, images are skull stripped and bias field corrected for uniformity. Lastly, images are displayed as a mosaic and saved into PowerPoint or a PDF for viewing. This process is then repeated for each time point

At the foundation of such a proposed unified approach to response assessment is a qualitative visual assessment of radiographic images over time, both before and during the therapies of interest. While it may seem obvious that such an evaluation is elemental, the current “side-by-side” comparison of images is flawed or at least suboptimal. We posit that we may be able to take advantage of the unique ability for motion perceiving neurons [18–20] in the middle temporal visual area (Fig. 4A–B) to identify subtle changes or “motion” in the images by turning these “side-by-side” comparisons into *videos* or “flipbooks,” with each page being a separate time point (Fig. 4C). Importantly, images should be standardized using international guidelines [21–23] and adequately registered using a rigid-body, 6 degrees of freedom (no stretching or skewing) to either a baseline or reference time point or to a standard radiographic atlas (e.g., MNI or Talairach template) in order to ensure the eyes are picking out the most relevant features within the images. While standardization of image acquisition and using the same scanner over time are best for consistent images over time, skull stripping to reduce differences in fat saturation parameters and bias field correction to adjust for differences in coil sensitivity may be desired. Lastly, the registered and processed images can be resliced and put into a mosaic (e.g., 6 images across and 4 images down every 3 mm from inferior to superior slices) and saved/imported into a PDF or PowerPoint (PPT) slide deck for easy viewing (note: use of transitions between slides can also be beneficial to enhance the visual changes). An example of how this process can be implemented using open-source tools is illustrated in Fig. 4D. First, images are converted from DICOM format into *NIFTI,* a standard 3D (or 4D) format used by many open-source neuroimaging software packages, using *dcm2niix* (part of many software packages, like AFNI, otherwise can be downloaded here: https://github.com/rordenlab/dcm2niix/releases). Next, *NIFTI* images can be registered using the *flirt* command in FSL [24–26] (https://fsl.fmrib.ox.ac.uk/fsl/fslwiki/FLIRT). Skull stripping can then be performed using the Brain Extraction Tool (*bet2*) (https://fsl.fmrib.ox.ac.uk/fsl/fslwiki/BET/UserGuide) and bias field correction can be performed using *fast* in FSL (https://fsl.fmrib.ox.ac.uk/fsl/fslwiki/FAST). Lastly, *AFNI* (https://afni.nimh.nih.gov) can be used for visualization and creation of the mosaic, which can then be saved and used in PowerPoint (see Supplemental Materials for an example of flipbooks).

## Modified Levin Criteria

**Fig. 5:**
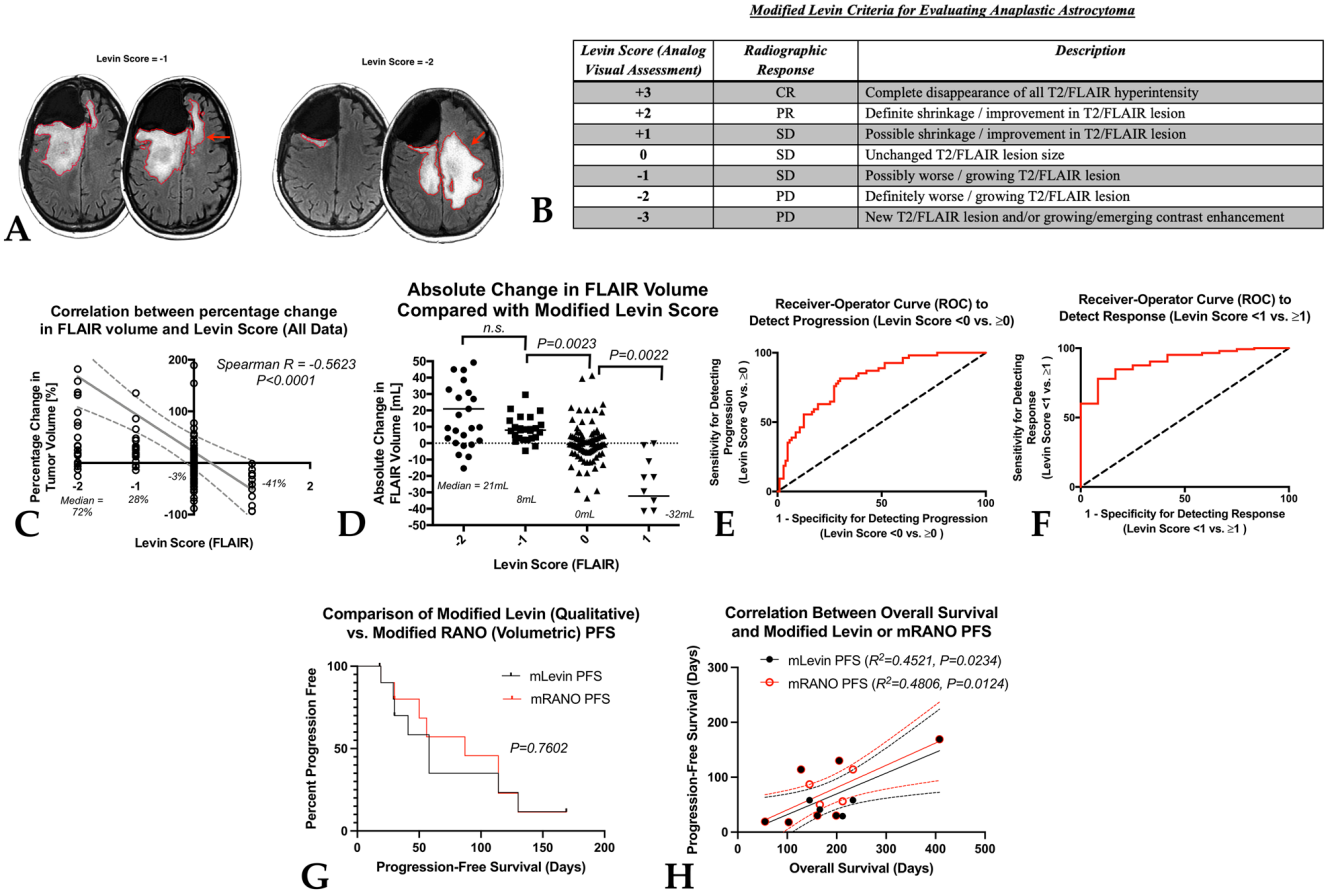
**A** Example T2-weighted FLAIR images showing modified Levin scores of “ − 1” and “ − 2,” corresponding to scans that are “possibly worse” and “definitely worse.” **B** Example modified Levin criteria for evaluating non-enhancing tumor in anaplastic astrocytomas. **C** Correlation between percentage change in FLAIR volume and modified Levin score (*Spearman, R* =  *− 0.5623, P* < *0.0001*). **D** Comparison between absolute change in FLAIR volume compared with modified Levin score. **E** Receiver operator curve (ROC) applied to FLAIR changes in order to detect progression. **F** ROC applied to FLAIR changes to detect response. **G** Kaplan–Meier curves comparing progression free survival (PFS) between modified RANO (volumetric) and the modified Levin criteria (*P* = *0.7602*). **H** Correlation between overall survival and modified Levin- or mRANO-defined PFS

In 1977, the Levin criteria [27] were introduced as a tool for evaluating brain tumor patients in how to interpret serial changes in neurological examinations, radionuclide scintiscans, CT scans, and EEG during chemotherapy and/or chemoradiation. This criterion presented an intuitive, numerical rating scale devised to designate change in tumor status and the degree of confidence or magnitude of these changes. As the next layer of our proposed “tiered” approach to response assessment, we offer a modification of these original Levin criteria can be added on top of the visual enhancement provided by digital flipbooks in order to add nuance to visual inspection (Fig. 5A–B). For example, non-enhancing tumor can be evaluated simply using the guide illustrated in Fig. 5B and a similar approach can be taken for both enhancing tumor (on post-contrast T1-weighted images) and non-enhancing tumor (on T2-weighted TSE or FLAIR images). Briefly, if no change in the tumor occurs between two sequential time points, the score would be “0” and would indicate stable disease (SD). If the tumor looks *possibly* larger or *possibly* smaller, or the degree of T2 hyperintensity looks worse or slightly more resolved, the score would be − 1 or + 1, respectively, with negative values denoting tumors looking worse. If there is increased confidence, where tumors look *definitely* worse or better, the score would be − 2 or + 2, reflecting progressive disease (PD) or partial response (PR), respectively. If the images demonstrate emerging enhancement or a new lesion, the score would be − 3 and would constitute PD. If there is complete disappearance of all T2 hyperintensity, the score would be + 3, and the patient would have a complete response (CR). Note that for both PR and CR, this would need to be durable for at least 4 weeks to constitute a confirmed PR or CR.

To show the link between these modified Levin criteria and lesion volume changes, we conducted a small retrospective study between Kaiser (San Francisco) and UCLA. A total of 37 anaplastic astrocytoma patients from Kaiser, evaluated at 5 time points for a total of 148 unique comparisons, and 40 anaplastic astrocytoma patients from UCLA, evaluated at 2 time points for a total of 40 unique comparisons, for a total of 188 comparisons were evaluated by an independent neuroradiologist at UCLA (Fig. 5C–D). Results suggested a median change in absolute tumor volume and percentage change necessary for a designation of “ − 2”, or PD, was 21 mL or 72%, respectively, while the median change was only 8 mL or 28% for a designation of “ − 1.” The median change observed for a modified Levin score of “0,” or no change, was 0 mL or around 3% change. Approximately 9 of the 188 comparisons illustrated “possible shrinkage,” or a score of + 1, which had a median change in FLAIR volume of – 32 mL or a percentage change of around − 41% between scans. No patients had scores of + 2 (definitely better), − 3, or + 3. There was a significant correlation between Levin score and the percentage change in tumor volume (Fig. 5C; *Spearman correlation, R* =  *− 0.5623, P* < *0.0001*). Results suggest a significant difference in absolute volume of FLAIR hyperintensity between Levin scores of “ − 1” vs. “0” (Fig. 5D; *Dunn’s test, P* = *0.0023*) and “ + 1” vs. “0” (*Dunn’s test, P* = *0.0022*), but no real difference between “ − 1” and “ − 2” (*possibly* vs. *definitely* worse). Receiver operator characteristic (ROC) analysis suggests a cutoff of around + 15% change in FLAIR volume provides a sensitivity of 78% and specificity of 71% for identifying progression, as defined by a Levin score of − 1 or − 2 (Fig. 5E; *ROC area under curve (AUC)* = *0.8046* ± *0.0351, P* < *0.0001*), while a cutoff of − 20% provided an 85% sensitivity and 83% specificity for detecting a response, as defined as a + 1 (Fig. 5F; *ROC AUC* = *0.9040* ± *0.0360; P* < *0.0001*). Together, these suggest a strong link between a qualitative “expert” review using a modified Levin criteria and quantitative tumor volume measurements in anaplastic astrocytoma.

In addition to quantifying the relationship between qualitative “expert” review and quantitative tumor volume measurements, we recently compared the progression-free survival (PFS) estimated using the modified RANO criteria (mRANO) [15] with the PFS calculated using a modified Levin criterion, applied to both T2 hyperintense and contrast enhancing tumor in 12 recurrent glioblastoma patients treated with osimertinib (Fig. 5G–H; ClinicalTrials.gov #NCT03732352). By defining the date of progression as the time from start of treatment to a *confirmed* PD event (e.g., a total score of − 2 followed by confirmation), we identified no difference in PFS between the quantitative mRANO criteria and the qualitative Levin criteria (Fig. 5G; *Log-rank, P* = *0.7602*). Additionally, we found a strong correlation between PFS and OS for both the mRANO criteria (Fig. 5H; *R*^*2*^ = *0.4806, P* = *0.0124*) and the Levin criteria (*R*^*2*^ = *0.4521, P* = *0.0234*), and no difference between the two (*P* = *0.7959*). Jointly, these data suggest that a qualitative, “expert” rating of the gestalt changes in brain tumor images can provide similar information to more detailed quantitative analysis.

In the case of mixed-grade or complex lesions, one might consider tracking *both* enhancing and non-enhancing tumor behaviors using similar criteria. Disease progression (PD) would then be determined by whether *either* enhancing or non-enhancing disease “definitely” progressed, possibly including confirmation, and response (PR) would require a “definite” response from *both* enhancing and non-enhancing tumor components. In the case of multifocal disease, evaluation should consider the behavior of all lesions, holistically, as it is outlined in the mRANO criteria for measurable lesions [15]. While it may be technically possible to determine changes for more than one discrete lesion, tracking changes over time becomes problematic due to complexity in determining how to manage the patient and trial when observing mixed responses—particularly when one lesion may be responding while the other growing. Additionally, quantifying response for individual lesions in multifocal disease becomes challenging when it is not clear whether a lesion is truly multifocal, as some multifocal enhancing lesions have contiguous T2 hyperintense regions connecting them, suggesting they are one (non-enhancing) tumor with two distinct foci of enhancement. Therefore, in practice, mixed responses should be gauged based on the gestalt. For example, if one lesion is larger and the other lesion is stable, then the gestalt would suggest that, overall, the tumor burden has increased, and, thus, the modified Levin score could be − 1 or − 2. If, on the other hand, one lesion has increased and another similarly sized lesion has decreased about the same degree, one might conclude that the overall tumor burden for the patient is unchanged, or a score of 0. While this approach is pragmatic and makes logistical sense from a clinical trial management perspective, it is important to note that tracking individual lesion responses may be beneficial for different circumstances, as the nature of the therapy might also impact the need to deal with the uncommon multifocal condition.

## Control Systems Theory for Quantifying Drug Behavior in Glioblastoma

**Fig. 6:**
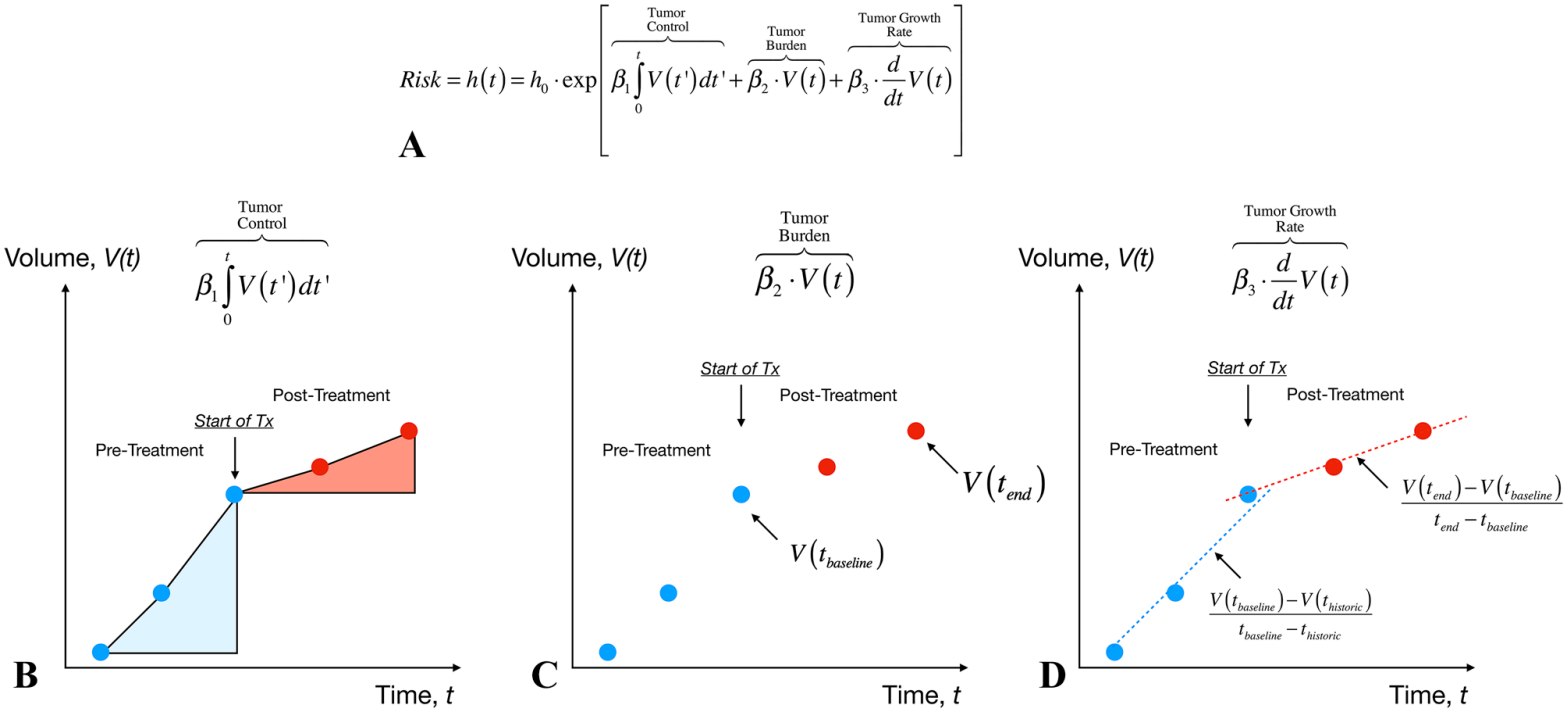
**A** Cox proportional hazard model including *proportional* (change in tumor burden), *integral* (tumor control), and *derivative* (tumor growth rate) components of tumor volume changes. **B** The “integral” component, or “area under the volume vs. time curve,” allows for evaluation and comparison of complex tumor volume changes by looking at the area before and after treatment. **C** The “proportional” component, corresponding to changes in the absolute volume, compares tumor volume between the current time point and the baseline. **D** The “derivative” component, reflecting the linear growth rate changes before and after treatment, can be used to determine whether tumor growth rates are altered as a result of treatment

Extensive clinical studies have shown a clear association between tumor size [28–37], change in tumor size (or growth rate) [28, 38–40], and survival in glioblastoma. This evidence appears to suggest a more comprehensive modeling approach that takes both parameters into account may be valuable. A logical expansion of the proportional (change in tumor volume) and derivative (growth rate) parameters is an *integral* parameter that loosely can be thought of as a *tumor control* parameter. The comprehensive risk associated with a given treatment can then be described as the linear combination of the *proportional*, *integral*, and *derivative* (PID) terms using a Cox proportional hazards model as (Fig. 6A):

$$\begin{aligned}Risk&=h\left(t\right)\\&={h}_{0}\cdot\mathrm{exp}\left[\overbrace{{\beta }_{1}\underset{0}{\overset{t}{\int }}V\left(t^{\prime}\right)dt^{\prime}}^{\mathrm{Tumor\;Control}}+\overbrace{{\beta }_{2}\cdot V\left(t\right)}^{\mathrm{Tumor\;Burden}}+\overbrace{{\beta }_{3}\cdot \frac{d}{dt}V(t)}^{\mathrm{Tumor\;Growth\;Rate}}\right]\end{aligned}$$

where *h(t)* is the risk or hazard as a function of time with base hazard *h*_*0*_, *β*_*1*_ is the coefficient for the risk associated with the *integral* or “tumor control” component, *β*_*2*_ is the coefficient for the risk associated with the *proportional* or “current tumor burden” component, *β*_*3*_ is the coefficient for the risk associated with the *derivative* or “growth rate” component, and *V(t)* is the tumor volume at time point *t*. One way to think of this is to compare measurements of these parameters before and after treatment. For example, the *integral* term would essentially compare the area under the volume vs. time curve (Fig. 6B). Conceptually, given the same time duration, if there is no change in growth rate, the areas under the curve should also be equivalent. However, if the tumor is stabilized for a period of time, no matter how convoluted the shape of the curve, this will manifest as a *smaller* area and thus a higher level of tumor control. Similarly, if the tumor shrinks, this will result in a *negative* area and similarly indicate a higher level of tumor control. Additionally, change in tumor size, as computed at the end of the treatment duration (*V(t*_*end*_*)*) minus the baseline time point (*V(t*_*baseline*_*)*), can be used similarly to interpret treatment effect, with smaller or negative differences indicating a stronger therapeutic effect (Fig. 6C). In the same fashion, changes in the linear growth rate over the treatment duration and a similar duration of time can be used to quantify the effects of new drugs, with smaller and more negative differences suggestive of stronger therapeutic effect (Fig. 6D). Using all three PID parameters allows for better characterization of how a therapy influences tumor growth behavior, as a therapy may be cytotoxic/cytoreductive and affect the *proportional* component, may stabilize, or control the tumor and influence the *integral* component, and/or it may slow down tumor growth rate and impact the *derivative* component of the model—all of which are meaningful for the survival of brain tumor patients.

**Fig. 7:**
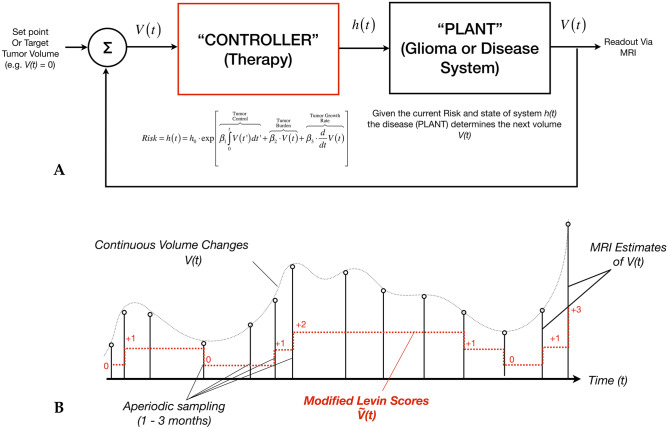
**A** Linear control systems theory applied to treatment response modeling. The “controller,” or therapy, is used to regulate or alter the “plant,” or disease system. The volume measured by MRI is used as the input to the “PID Controller” or model. The output of the controller is the hazard ratio, or influence of the therapy on the disease. Given the relative risk resulting from the treatment, the disease system then determines the tumor volume at the next time point. Note that if no treatment is applied, the plant or disease is uncontrolled and the tumor volume vs. time is dictated purely from the disease system. In order to model continuous tumor growth behavior, *V(t)*, it must first be described in terms of discrete or sampled data as measured by MRI estimates of *V(t)*. This sampling, however, is often aperiodic (e.g., between 1 and 3 months for glioblastoma) depending on if the patient is exhibiting rapid changes that require more frequent observation. Like quantitative measurements of tumor volume, *qualitative* modified Levin scores can be used over time as an estimate of tumor growth behavior $\tilde{V}\left( t \right)$ (red) and used in the same types of control system models

The aforementioned PID components, and their mathematical definitions, can conveniently be described and characterized using linear control systems theory [41, 42]. Using this theory, the therapy or therapeutic regimen can be thought of as a “PID controller” [43–45] for the “disease” system (or “Plant”) (Fig. 7A), much like how a car’s speed controller is used to control the car (or Plant) by setting a certain target speed for the control system and the controller then reacts to bring the plant to its desired velocity. The “set point” for the tumor volume (desired “speed”) might be naturally desired to be a value of zero, a complete response, making the current tumor volume the direct input to the “controller”, or therapeutic paradigm. Using historic volumetric data, the internal characteristics of this controller then determines how this new measurement of tumor volume will influence the hazard or risk to the patient, *h(t)*, using the characteristics described by the PID model. Given this risk, *h(t)*, the characteristics of the patient’s tumor then dictate how this adjustment in risk is translated into some change in tumor volume for the next time point, which can be measured using MRI.

It is intuitive to think of the treatment regimen for a brain tumor patient as some sort of control system for a runaway, uncontrollable vehicle that continues to accelerate. Beyond this convenient metaphor, the use of control systems theory in this context provides powerful insight into how to optimize a therapeutic regimen in the context of drug development or clinical management of brain tumors. Knowing something about the timing of the expected response, either from preclinical models, pharmacodynamics/pharmacokinetics, or the natural history of the disease, it may be possible to provide insight into how frequently to scan patients using principles outlined in discrete control system theory [46] (i.e., Nyquist criteria for stability [47] and non-periodic sampling schemes[48]). Given enough data, it may even be possible to perturb a system using different types of therapies and characterize the “plant” or disease state for different genetic subtypes of tumor, or even predict future tumor growth and treatment resistance [49].

**Fig. 8:**
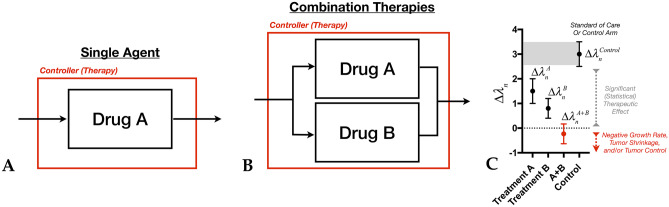
**A** The effects of a single drug “A” can be modeled as a single system block element with a known transfer function *R*_*A*_*(s)* by quantifying the change in the area under the volume vs. time curve, $$\begin{aligned}\Delta {\lambda }_{1}^{A}&=({\lambda }_{1-PostTx}^{A}-{\lambda }_{1-PreTx}^{A})\\&=\left(\stackrel{PostTx}{\overbrace{\underset{t=0}{\overset{t=+6mo}{\int }}V(t^{\prime})-V(0)dt^{\prime}}}-\stackrel{PreTx}{\overbrace{\underset{t=-6mo(historic)}{\overset{t=0}{\int }}V(t^{\prime})-V({t}_{historic})dt^{\prime}}}\right)\end{aligned}$$, the difference in volume, $\Delta {\lambda }_{2}^{A}=({\lambda }_{2-PostTx}^{A}-{\lambda }_{2-PreTx}^{A})=\left(\stackrel{PostTx}{\overbrace{V(t=6mo)}}-\stackrel{PreTx}{\overbrace{V(t=0)}}\right)$, and the change in linear growth rate, $\Delta {\lambda }_{3}^{A}=({\lambda }_{3-PostTx}^{A}-{\lambda }_{3-PreTx}^{A})=\left(\stackrel{PostTx}{\overbrace{\frac{d}{dt}{V(t)|}_{t=0}^{t=+6mo}}}-\stackrel{PreTx}{\overbrace{\frac{d}{dt}{V(t)|}_{t=-6mo}^{t=0}}}\right)$. (Note that in this example, the duration of evaluation is 6 months prior to treatment and 6 months after start of treatment.) **B** In combination therapies, a similar approach can be taken where each parameter is quantified $\Delta {\lambda }_{n}^{A+B}=\left({\lambda }_{n-PostTx}^{A+B}-{\lambda }_{n-PreTx}^{A+B}\right)$ for each patient. **C** Since each PID parameter is calculated for each individual patient, the *distributions* of these parameters can be statistically compared between treatment conditions, including individual treatments, “*A”* or *“B,”* and combinations *A* + *B* (red) with respect to historic or prospective control group receiving standard therapies

To operationalize this concept, continuous control systems theory needs to be described in terms of *discrete* or digital systems theory [46], as the continuous volumes are only sampled at specific time points during clinical MRI visits (Fig. 7B). For non-measurable or complex lesions, the modified Levin scores can also be digitized and tracked over time, added up to create an estimate of tumor behavior albeit with less granularity. There are two conceivable ways to utilize this concept for drug development or in a clinical trial, both of which require standardizing the pre-treatment and post-treatment evaluation period for adequate comparison. The first approach includes the full feedback model as described mathematically in Fig. 6A and graphically on Fig. 7A in order to characterize the *influence of PID parameters directly on the Cox hazard ratio*. With this more comprehensive approach, it may be theoretically possible to interrogate, in real time, the impact of the different drugs with respect to administered dose and frequency of dosing, and then add combinations to quantify the impact on growth patterns. The second, more practical approach for early trials would include *characterizing the change in PID variables before and after therapy*, then comparing to an adequate control or reference arm. As an example, standardizing the evaluation period to a 6-month pre-treatment period and 6 months on study for recurrent glioblastoma may allow for a judicious comparison between PID parameters by quantifying tumor burden (*proportional*), growth rates (*derivative*), and “area under the volume vs. time curve” (*integrative*). While this approach does not directly link change in tumor behavior with outcome vis-à-vis the hazard ratio, it nonetheless can be used to characterize “subclinical” therapeutic effects via PID parameters. To realize this, one can calculate the area under the volume vs. time curve *before* treatment “A” (${\lambda }_{1-PreTx}^{A}$) and *after* treatment “A” (${\lambda }_{1-PostTx}^{A}$) for a group of patients, then look at how therapy “A” has *changed* this parameter (Fig. 8A):

$$\begin{aligned}\Delta {\lambda }_{1}^{A}&=({\lambda }_{1-PostTx}^{A}-{\lambda }_{1-PreTx}^{A})\\&=\left(\stackrel{PostTx}{\overbrace{\underset{t=0}{\overset{t=+6mo}{\int }}V(t^{\prime})-V(0)dt^{\prime}}}-\stackrel{PreTx}{\overbrace{\underset{t=-6mo(historic)}{\overset{t=0}{\int }}V(t^{\prime})-V({t}_{historic})dt^{\prime}}}\right)\end{aligned}$$

This can similarly be applied to change in tumor burden at baseline and after the treatment period:

$$\begin{aligned}\Delta {\lambda }_{2}^{A}&=({\lambda }_{2-PostTx}^{A}-{\lambda }_{2-PreTx}^{A})\\&=\left(\stackrel{PostTx}{\overbrace{V(t=6mo)}}-\stackrel{PreTx}{\overbrace{V(t=0)}}\right)\end{aligned}$$

and changes in tumor growth rate over the same interval:

$$\begin{aligned}\Delta {\lambda }_{3}^{A}&=\left({\lambda }_{3-PostTx}^{A}-{\lambda }_{3-PreTx}^{A}\right)\\&=\left(\stackrel{PostTx}{\overbrace{\frac{d}{dt}{V(t)|}_{t=0}^{t=+6mo}}}-\stackrel{PreTx}{\overbrace{\frac{d}{dt}{V(t)|}_{t=-6mo}^{t=0}}}\right)\end{aligned}$$

Here, $\Delta \lambda_{n}^{A}$ is the change in the parameter *n* before and after treatment *A*. Note that the pre- and post-treatment evaluation period could be shorter or longer depending on the characteristics of the tumor (i.e., slower or faster growing tumor may require more sparse or frequent evaluation periods).

Statistically, the distribution of these parameters across all patients in a treatment arm can be compared to a theoretical value or values from a control group, with the hypothesis that the experimental treatment PID parameters are *lower* than the expected control value, or $\Delta {\lambda }_{n}^{A}<\Delta {\lambda }_{n}^{Control}$ (i.e., if the treatment is working, the area, volume, and growth rate should be *lower* in the post-treatment setting compared to the pre-treatment setting). Combination therapies can be treated the same way (Fig. 8B), with parameters can be recalculated for each patient in this new arm using the same pre- and post-treatment duration, resulting in $\Delta {\lambda }_{1}^{A+B},\Delta {\lambda }_{2}^{A+B},\Delta {\lambda }_{3}^{A+B}$ for the combination setting. The distribution of these parameters can then be used to determine the probability of a lower parameter value (better response) compared with the single agent setting (Fig. 8C), or $p(\Delta {\lambda }_{n}^{A+B}<\Delta {\lambda }_{n}^{A})$. This approach can easily be used to determine whether combinations are affecting growth patterns and whether these alterations are synergistic. Additionally, this approach addresses some of the fundamental issues surrounding the use of combination therapies in brain tumors including inefficiencies, expenses, and the long trial durations necessary to test sequential combinations through the identification of potential *subclinical* effects independent on their impact on survival.

It is important to note that with more complex modeling, the variance from individual parameters and measurements can be amplified when calculating the overall risk. Assuming that the errors in volume or size measurement are random and normally distributed, difference calculations will result in *twice* the variance of the original measurements. Similarly, the variance in the difference parameters used to estimate treatment effect before and after treatment, $\Delta {\lambda }_{n}^{A}$, will also exhibit approximately double the variability of the parameter in either the pre-treatment or post-treatment setting. For growth rate estimation, a linear slope estimator can be used to generalize the growth rate over the entire evaluation period, generally reducing the variability, while numerical integration approaches including the trapezoidal [50], midpoint [50], or Simpson’s rules [51] can help reduce the amplified variability in the integral terms from the difference calculations for both tumor size and time differences.

**Fig. 9:**
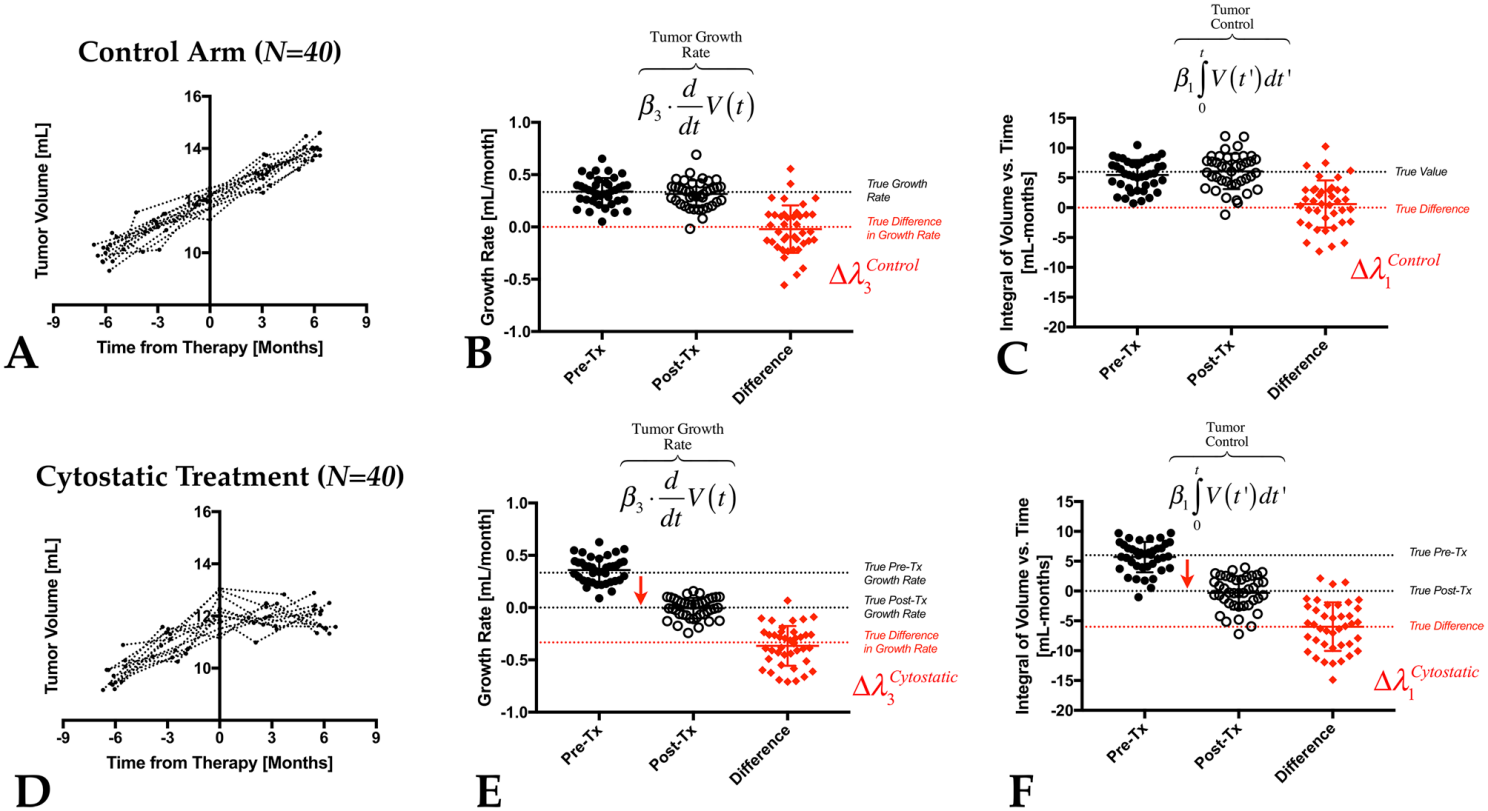
Simulated clinical trial data showing use of growth rates and tumor control estimations. A total of *N* = *80* slow-growing gliomas with baseline tumor size of 10 mL ± 0.5 (S.D.), 6 months prior to treatment, growing at a rate of 1 mL every 3 months were simulated. Perturbations in tumor volume were simulated using a normal distribution with variability of 0.25 mL (~ 250 voxels). The baseline tumor volume just prior to treatment was 12 mL ± 0.5 (S.D.) for all patients. The scan date was also simulated every 3 months ± 2 weeks(S.D.) to mimic the clinical variability often seen with regard to the date of the MRI exam. **A** Volumetric growth rate simulations in patients randomized to the control arm with no change in growth rate. **B** Tumor growth rates estimated through linear regression across the pre-treatment and post-treatment time points, as well as the difference in growth rates. **C** Tumor control measurements before and after treatment in the control arm showing no difference in area under the volume vs. time curves. **D** Volumetric growth rate simulations in patients randomized to a cytostatic agent that slows or stabilizes the growth of the tumors. **E** Tumor growth rates and **F** tumor control measurements in the cytostatic treatment arm showing quantifiable reductions even in the presence of noise

To demonstrate the operation of this approach and put this mental exercise into practice, 80 relatively slow-growing tumors with baseline tumor size of 10 mL were simulated 6 months prior to treatment, growing at a rate of 1 mL every 3 months (0.33 mL/month). Perturbations in tumor volume were simulated using a normal distribution with variability of 0.25 mL (~ 250 voxels), leading to an approximate pre-treatment baseline tumor volume of 12 mL ± 0.5 (S.D.). The scan date was simulated every 3 months ± 2 weeks (S.D.) to mimic the clinical variability often seen with regard to the date of the MRI exam. We then assumed a 1:1 randomization between a treatment with no effect (*N* = *40*), so the tumor grew at the same rate after treatment (Fig. 9A), and a treatment that stabilized or flattened the growth rate (Fig. 9D, N = *40*). In the simulated control arm (Fig. 9B), the linear growth rate estimated over the 6 months prior to treatment was 0.338 ± 0.130(S.D.) mL/month, and after the ineffective therapy, the growth rate averaged 0.317 ± 0.129(S.D.) mL/month, with a difference in growth rates of − 0.021 ± 0.227 (S.D.) mL/month not significantly different from zero (*P* = *0.5573*) (note that the standard deviation was almost twice in the difference measure). Similarly, the integral of the volume vs. time curve (Fig. 9C), or tumor control, averaged 5.466 ± 2.512 (s.d) and 6.080 ± 2.975 (s.d) mL/month, respectively, and the difference in tumor control for this ineffective treatment was 0.615 ± 3.96 (s.d) mL/months (*P* = *0.3322*). By simulating a cytostatic treatment that stabilizes or slows down tumor growth using the same noise parameters (Fig. 9E), we can see that the tumor growth rate after treatment averaged − 0.006 ± 0.098 (S.D.) mL/month, resulting in a growth rate of − 0.366 ± 0.191 (S.D.) mL/month, significantly lower than zero (*P* < *0.0001*). Similarly, tumor control rates showed a significant decrease after treatment (Fig. 9F), averaging − 0.286 ± 2.778 (S.D.) mL/months, resulting in a change in tumor control of − 5.983 ± 4.068 (S.D.) mL/months (*P* < *0.0001*). Together, these simulated data demonstrate how the aforementioned concepts can be operationalized to identify and evaluate ineffective and effective treatments for brain tumors.

Although specific strategies surrounding optimization of combination therapies are beyond the scope of this current manuscript, it is worth mentioning that the most common approach glioma therapies over the past decades has been to combine a new agent with an alkylating agent, either temozolomide or lomustine, solely because they have been considered standards for care of recurrent and or progressive gliomas over the past decades. Another approach, especially relevant to cell signaling inhibitors, would be to combine two drugs that might have complementary antitumor activity based on preclinical science. It would also be equally logical to try and interfere with a signaling pathway in two locations as opposed to seeking to inhibit a secondary pathway. Both approaches have merit, and both would benefit from a new and better-informed approach to evaluate whether the new combination is “effective.” In our present regulatory world “effective” means, at the very least the ability of the therapy to prevent tumor growth and, at its apogee, increased survival. While we cannot argue about this approach, we can argue from decades of experience that it is an inefficient, long-duration, and expensive approach. Furthermore, learning from the failed experimental trial may be limited. A better approach might be to design an experiment where one interrogates the impact of the first drug with respect to administered dose and frequency of dosing on tumor growth dynamics, and then the second drug is added to quantify the combination benefits and resulting alterations in growth trajectory. Although the details of such an approach remain to be determined, it is conceivable that the framework outlined above, evaluated over intervals of weeks to a couple of months, can aid and guide this quest. Importantly, a good understanding of the experimental drugs are required, since there needs to be a balance between acquiring enough data to make an informed decision about a treatment and limiting potential harm from keeping a patient on an ineffective therapy.

## Conclusion

There is an urgent need for drug development in brain tumors. Current approaches for radiographic response assessment are limited, particularly for complex or difficult to measure tumors, mixed grades, or if therapeutic effects are subtle. We propose a tiered strategy that increases confidence in identifying therapeutic effects, while also providing some creative ideas for how to overcome many of the limitations associated with challenging tumor types or scenarios. To be truly valuable in drug development, this approach to radiographic drug assessment must be coupled to a good understanding of the mechanisms of action of novel drugs being investigated with this methodology.

## Supplementary Information

Below is the link to the electronic supplementary material.

Supplementary file1 (PPTX 23893 KB)

Supplementary file2 (DOCX 34 KB)

Supplementary file3 (DOCX 48 KB)

Supplementary file4 (DOCX 34 KB)
