# Supplementary material for: Radiographic Response Assessment Strategies for Early-Phase Brain Trials in Complex Tumor Types and Drug Combinations: from Digital “Flipbooks” to Control Systems Theory
Source: Neurotherapeutics. 2022 Apr 22;19(6):1855–68. doi: 10.1007/s13311-022-01241-8 (PMC9723080; doi:10.1007/s13311-022-01241-8)
Supplement: Supplementary file 1 — Supplementary file1 (PPTX 23893 KB) [file 13311_2022_1241_MOESM1_ESM.pptx]

## Slide 1
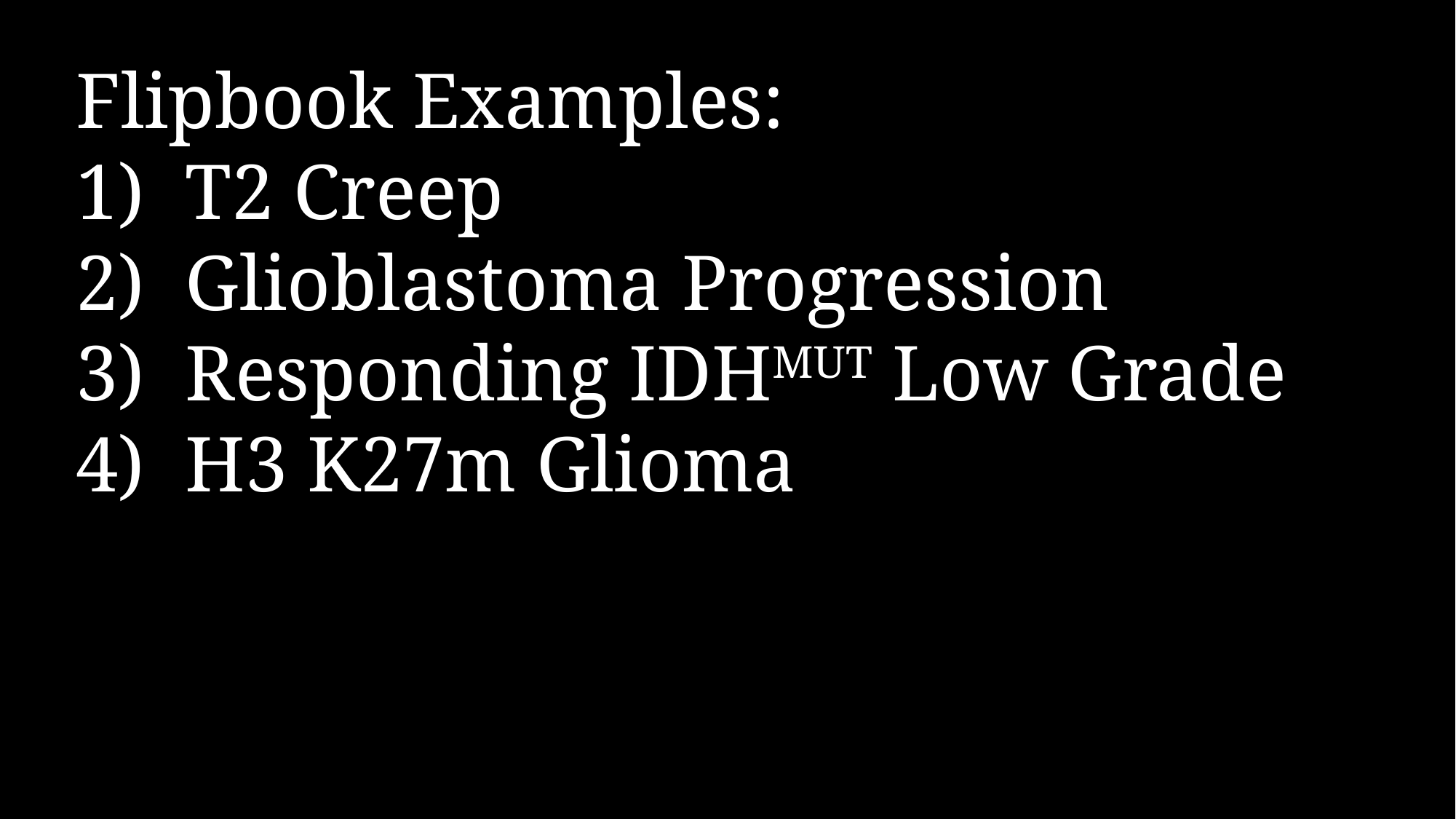

Flipbook Examples:
T2 Creep
Glioblastoma Progression
Responding IDHMUT Low Grade
H3 K27m Glioma

## Slide 2
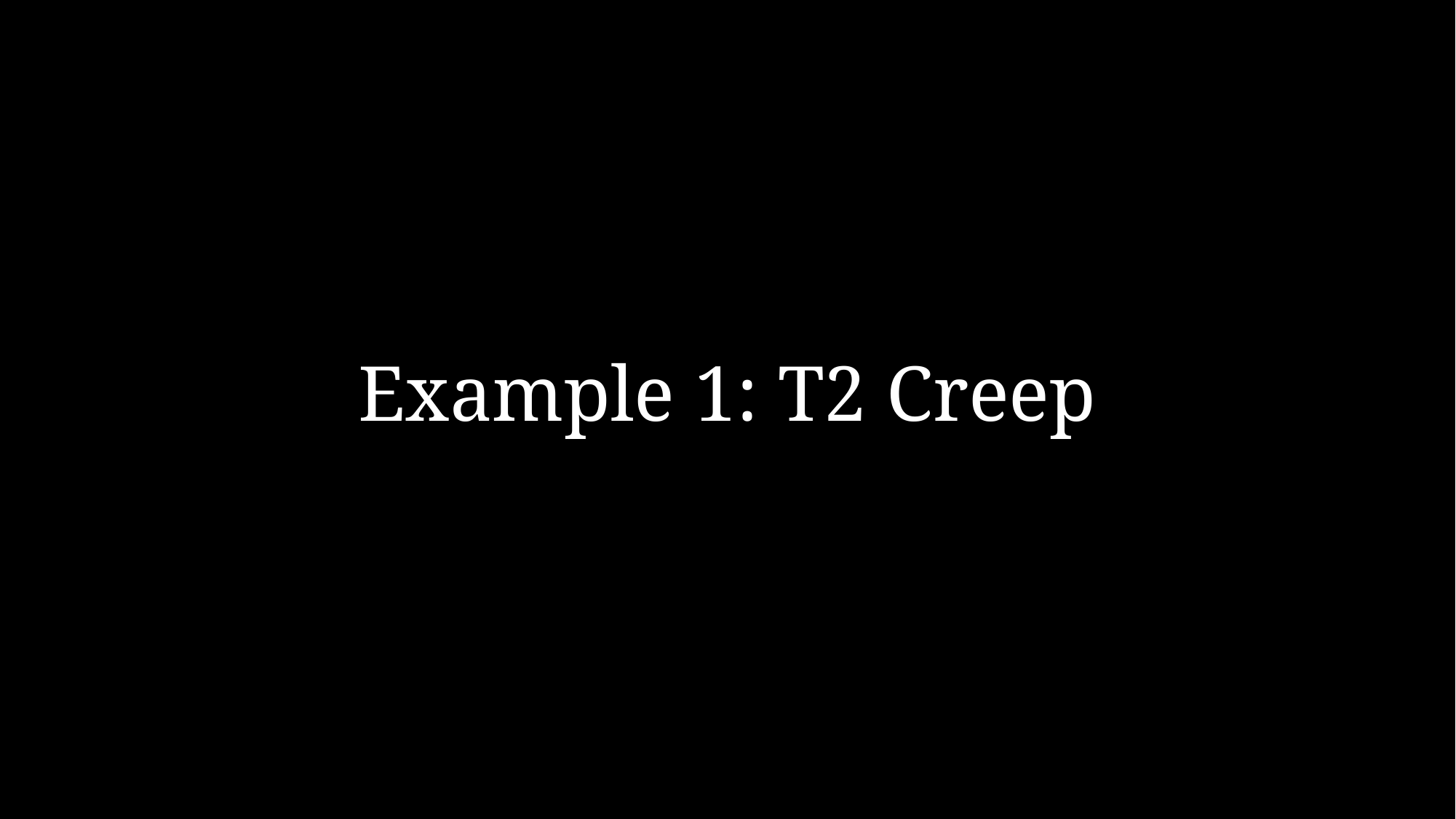

Example 1: T2 Creep

## Slide 3
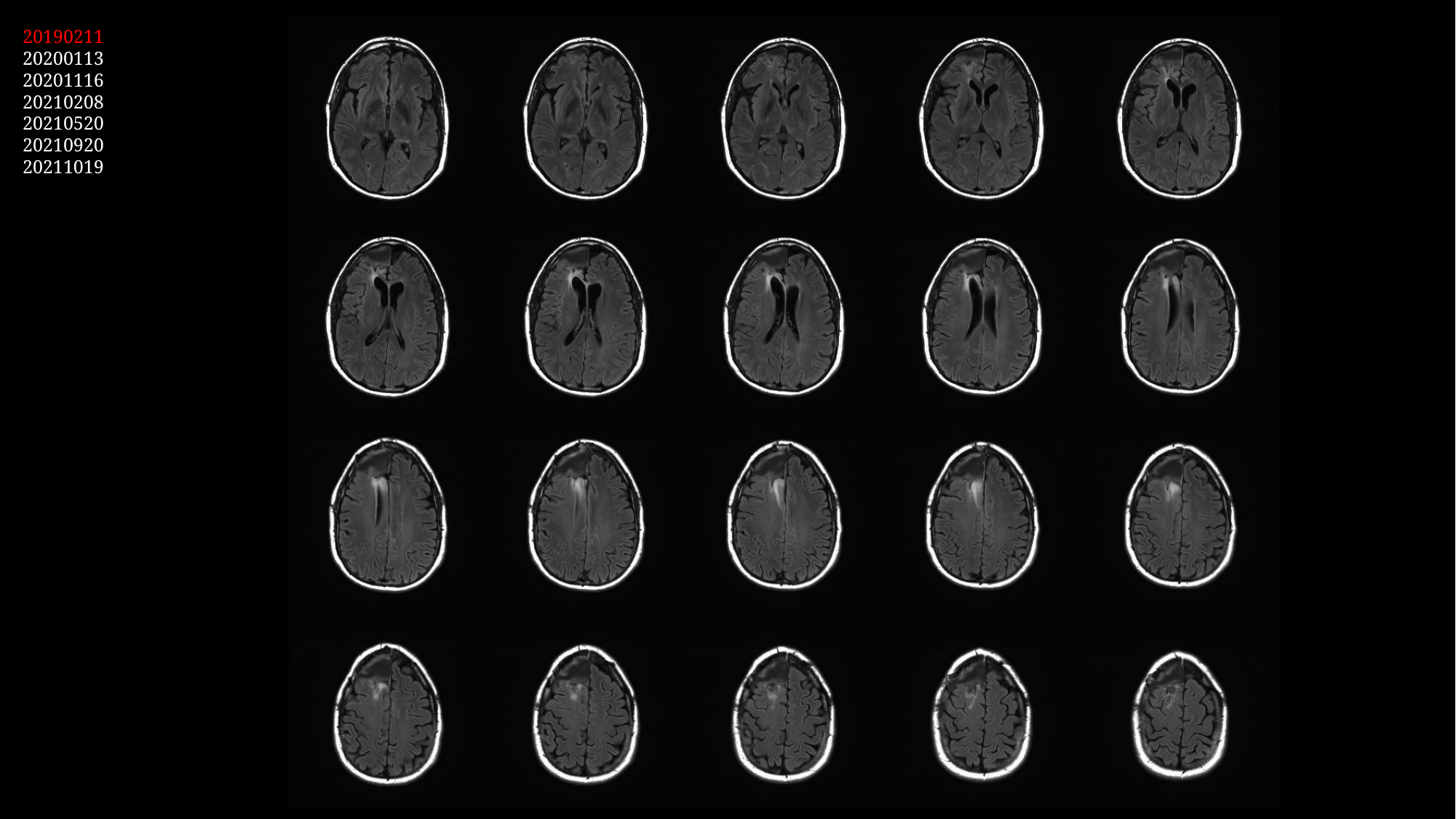

20190211
20200113
20201116
20210208
20210520
20210920
20211019

## Slide 4
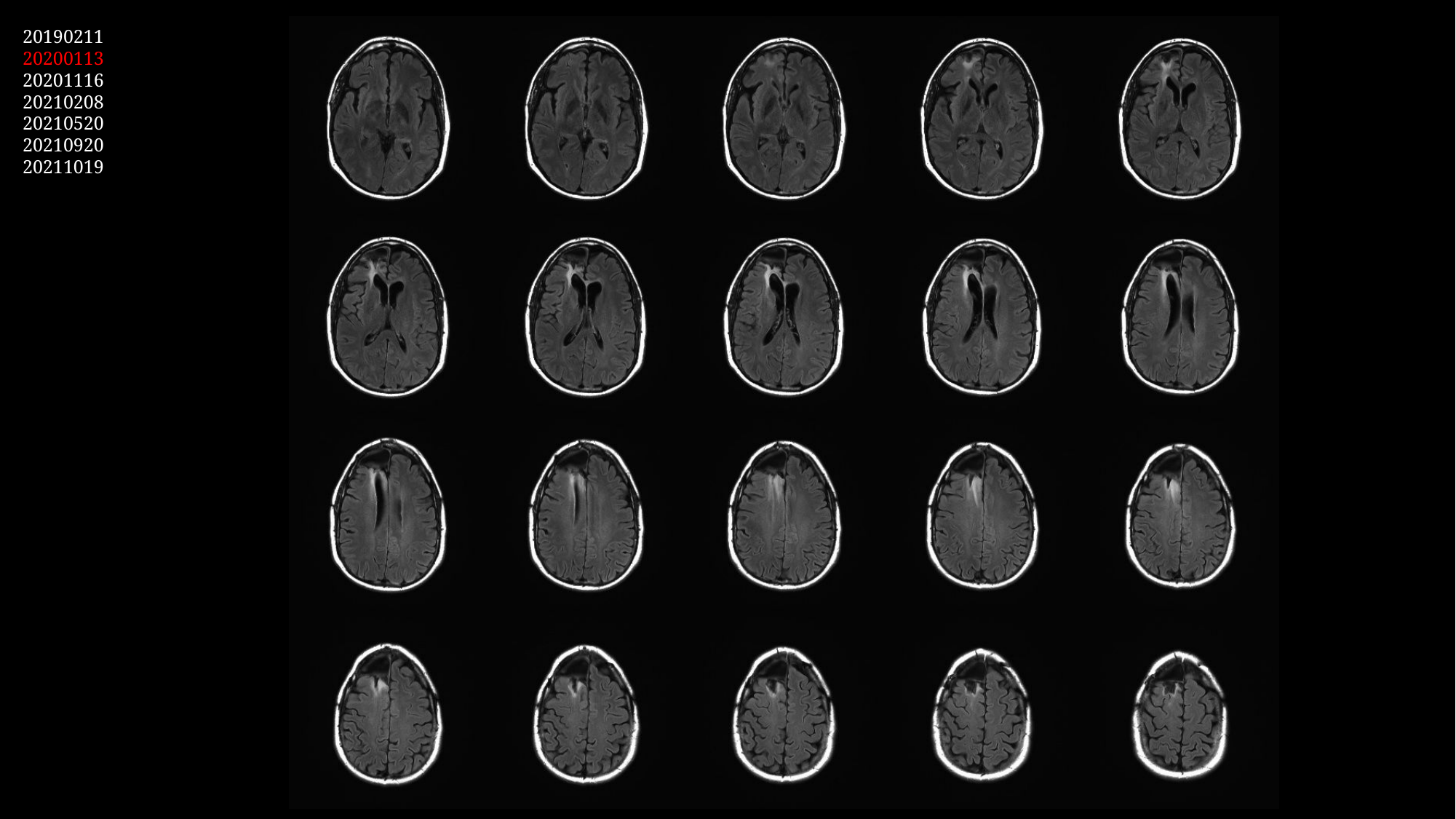

20190211
20200113
20201116
20210208
20210520
20210920
20211019

## Slide 5
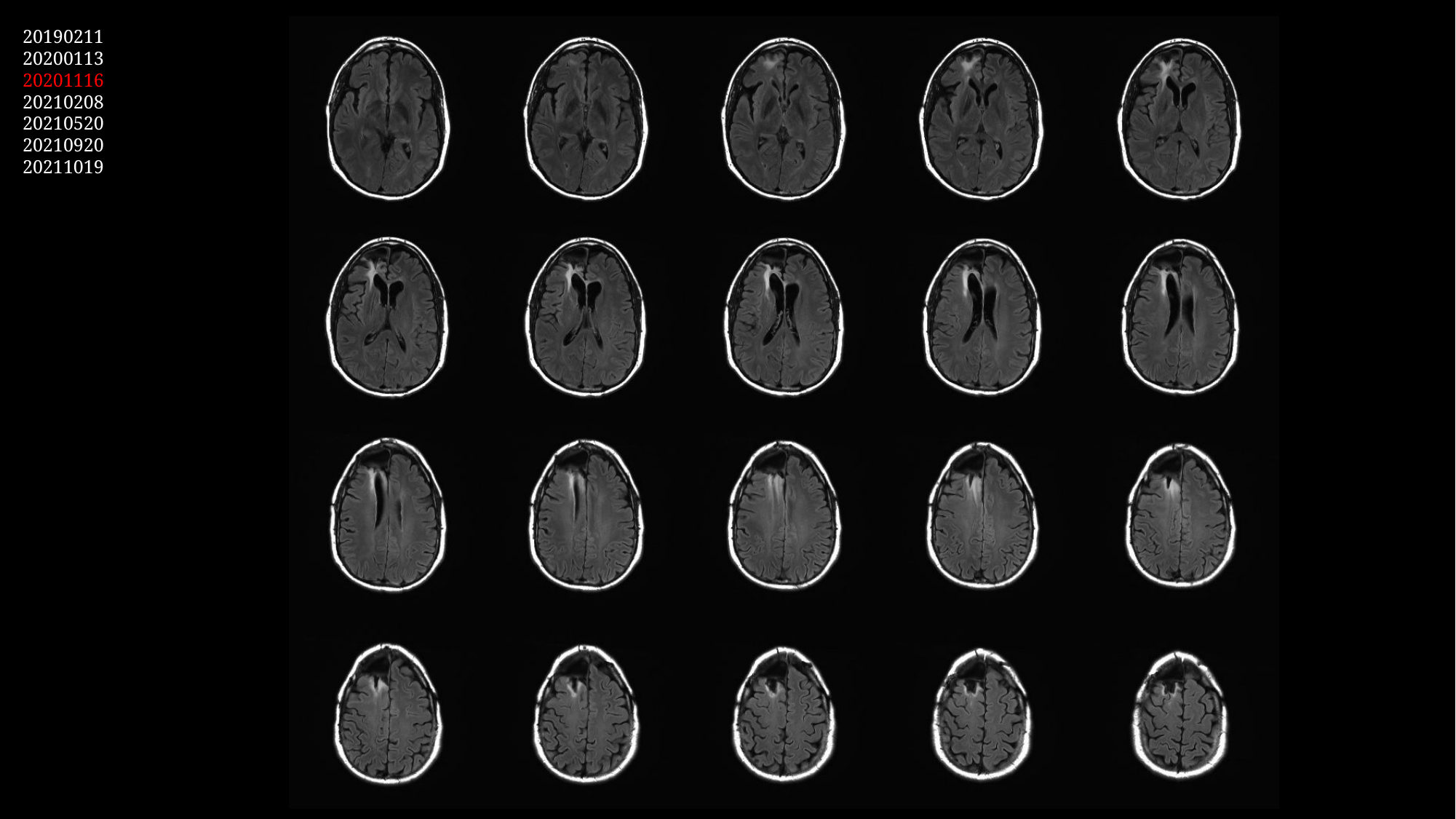

20190211
20200113
20201116
20210208
20210520
20210920
20211019

## Slide 6
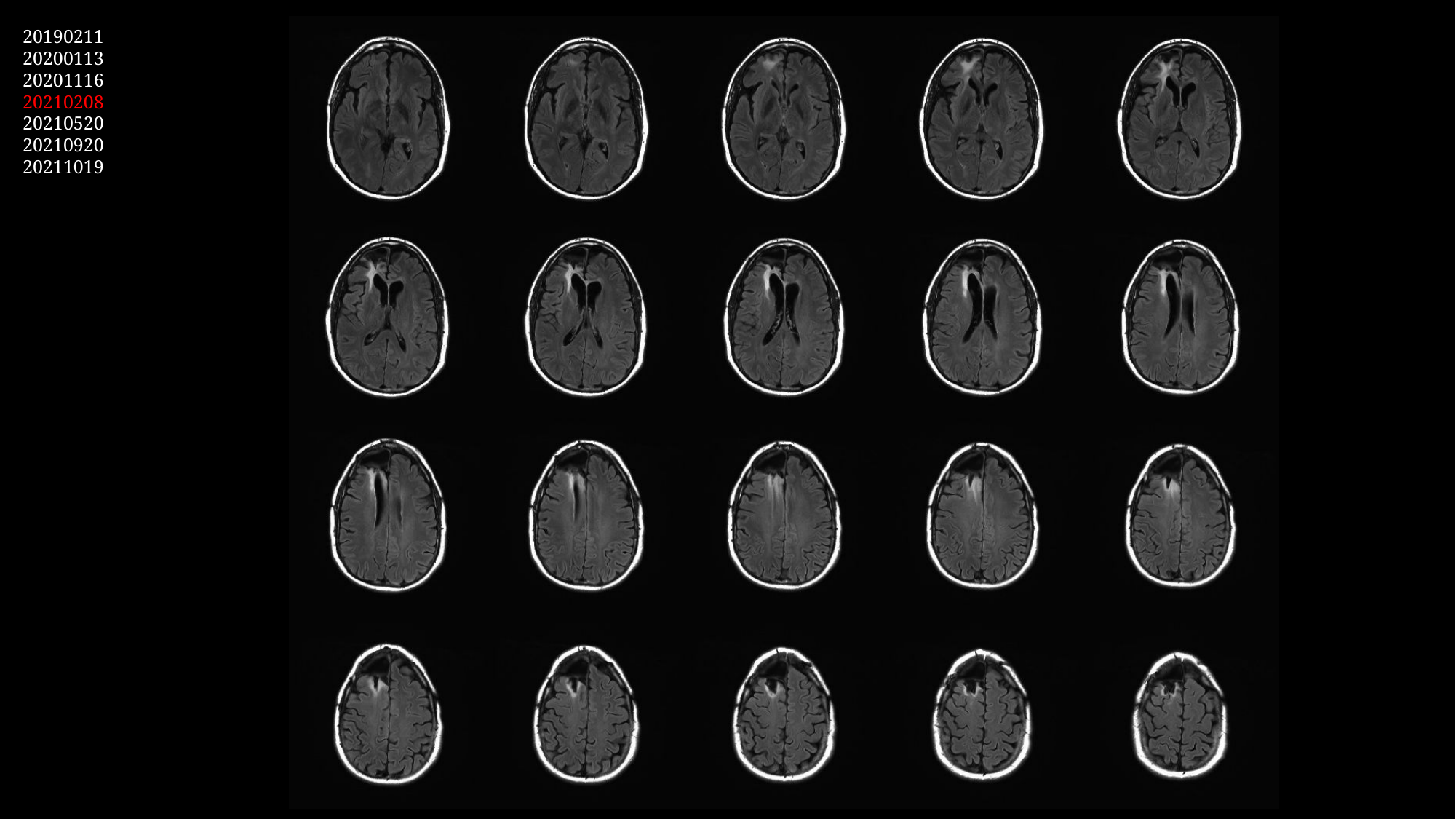

20190211
20200113
20201116
20210208
20210520
20210920
20211019

## Slide 7
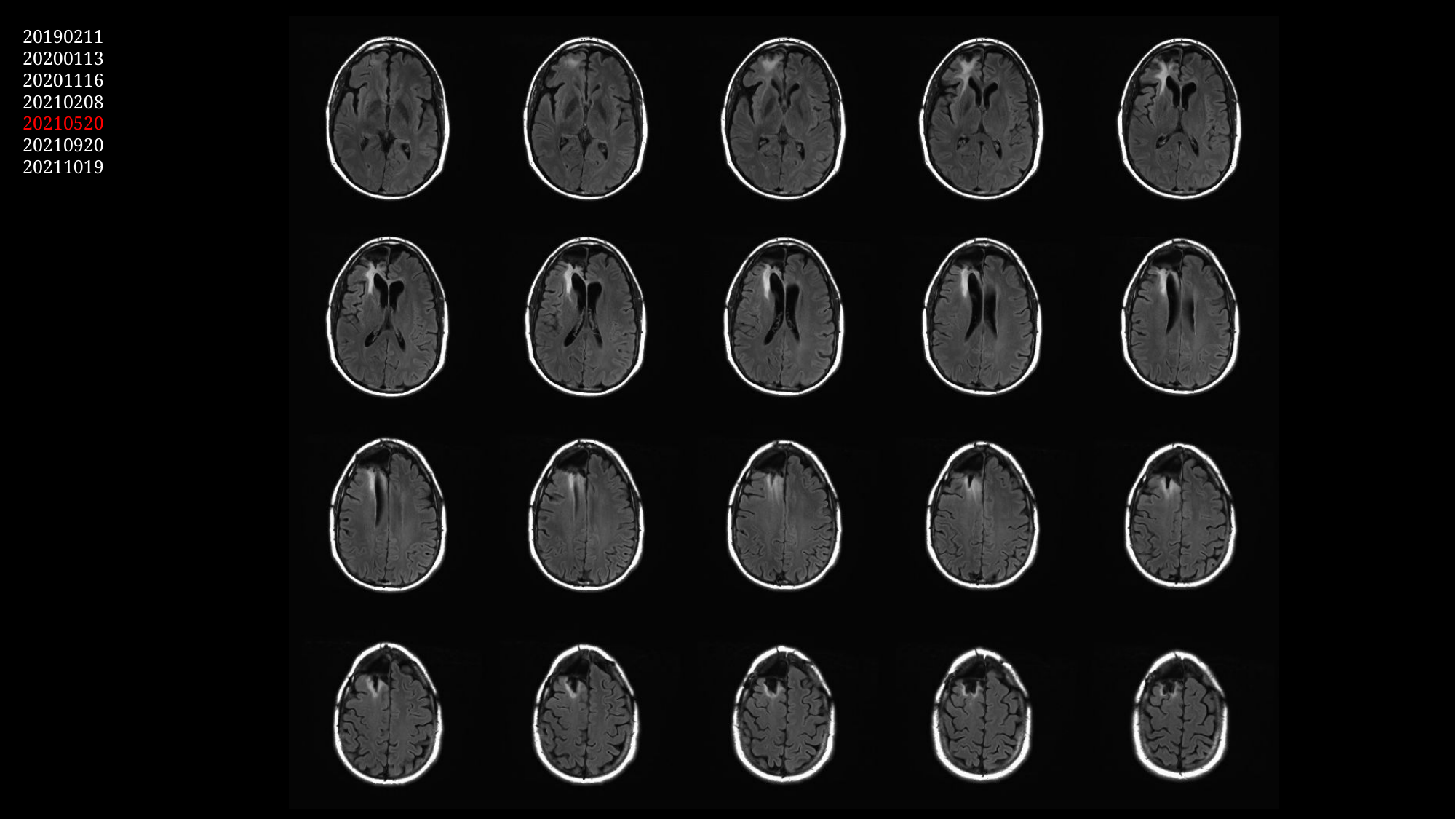

20190211
20200113
20201116
20210208
20210520
20210920
20211019

## Slide 8
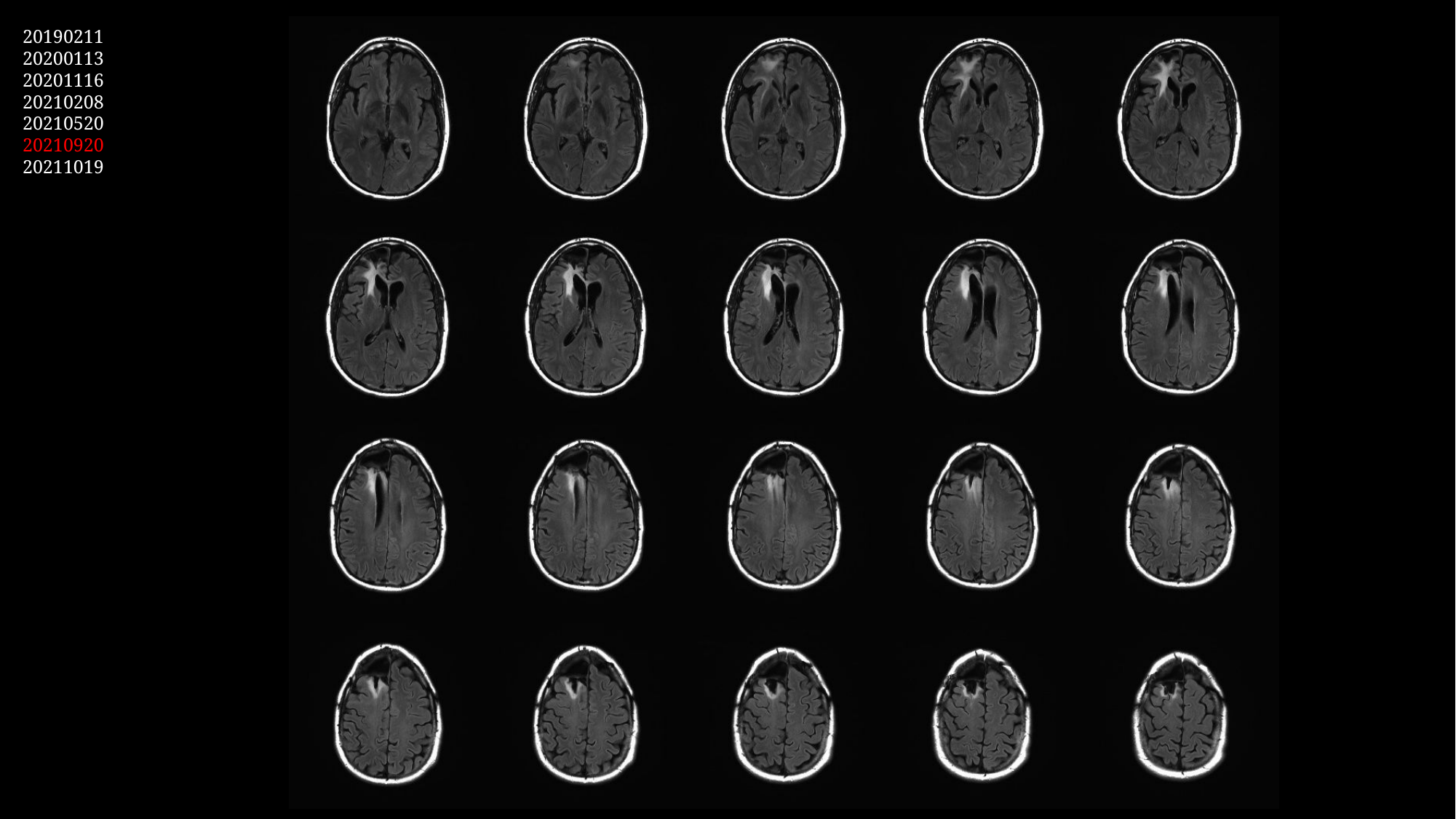

20190211
20200113
20201116
20210208
20210520
20210920
20211019

## Slide 9
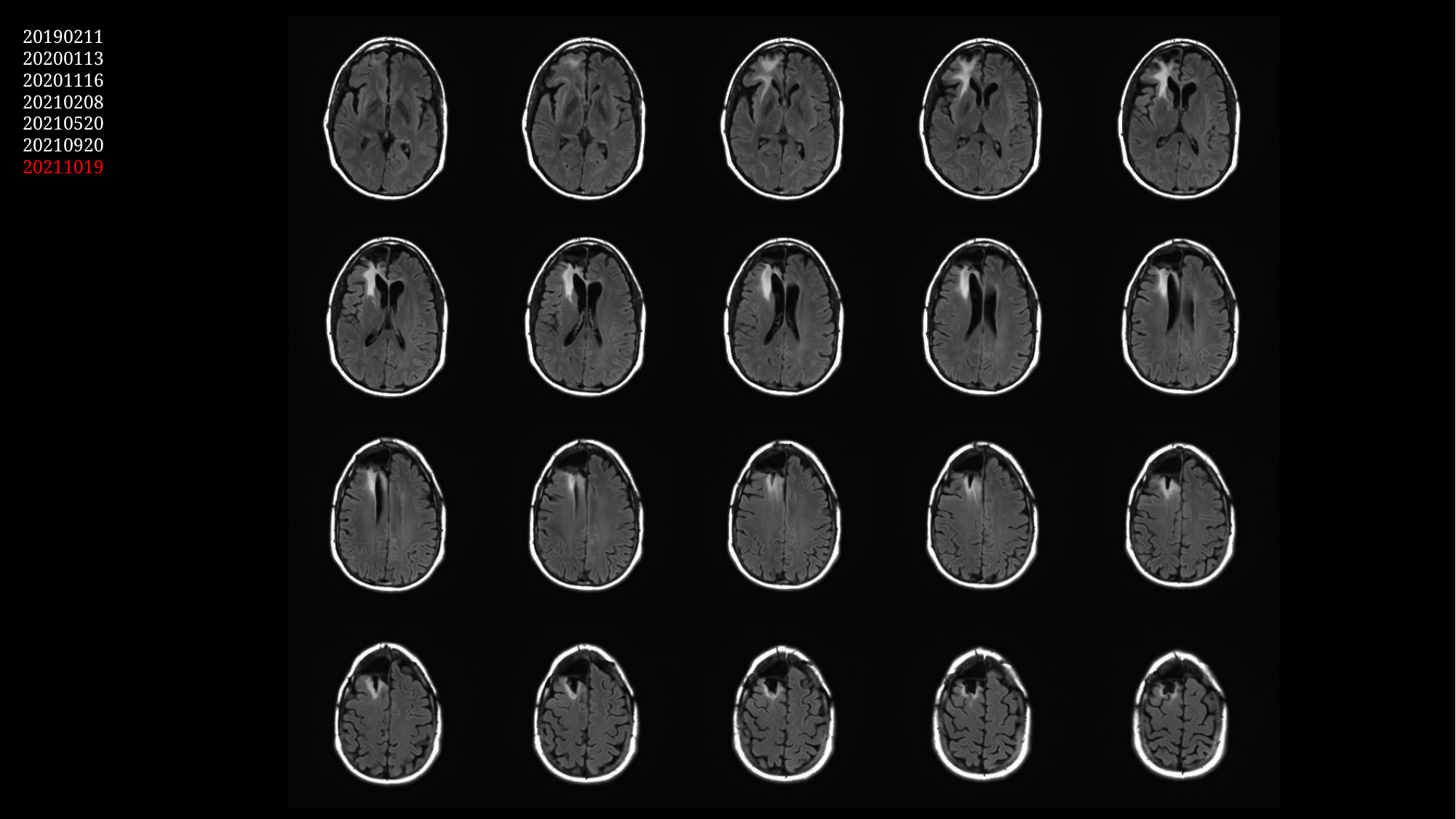

20190211
20200113
20201116
20210208
20210520
20210920
20211019

## Slide 10
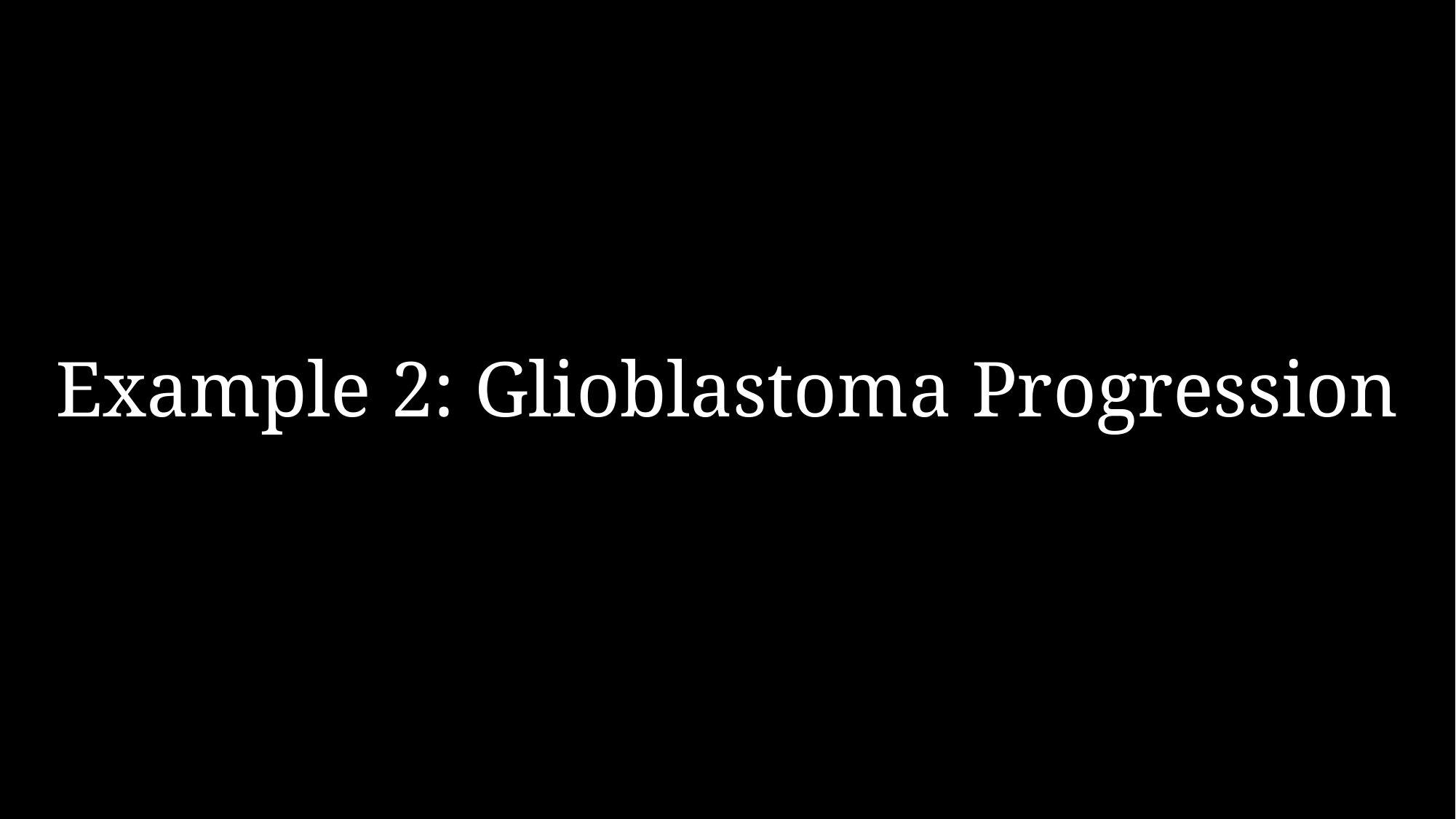

Example 2: Glioblastoma Progression

## Slide 11
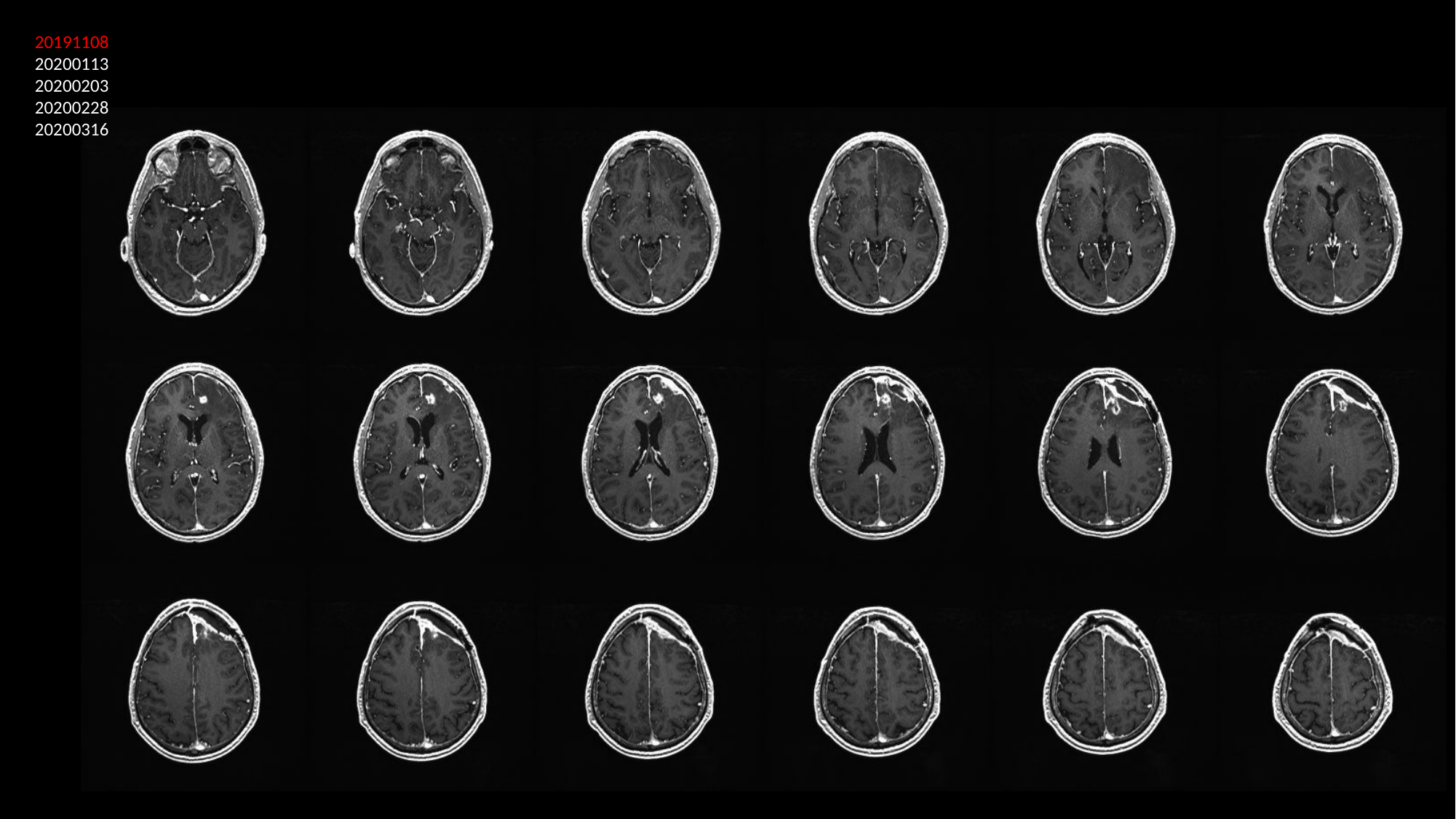

20191108
20200113
20200203
20200228
20200316

## Slide 12
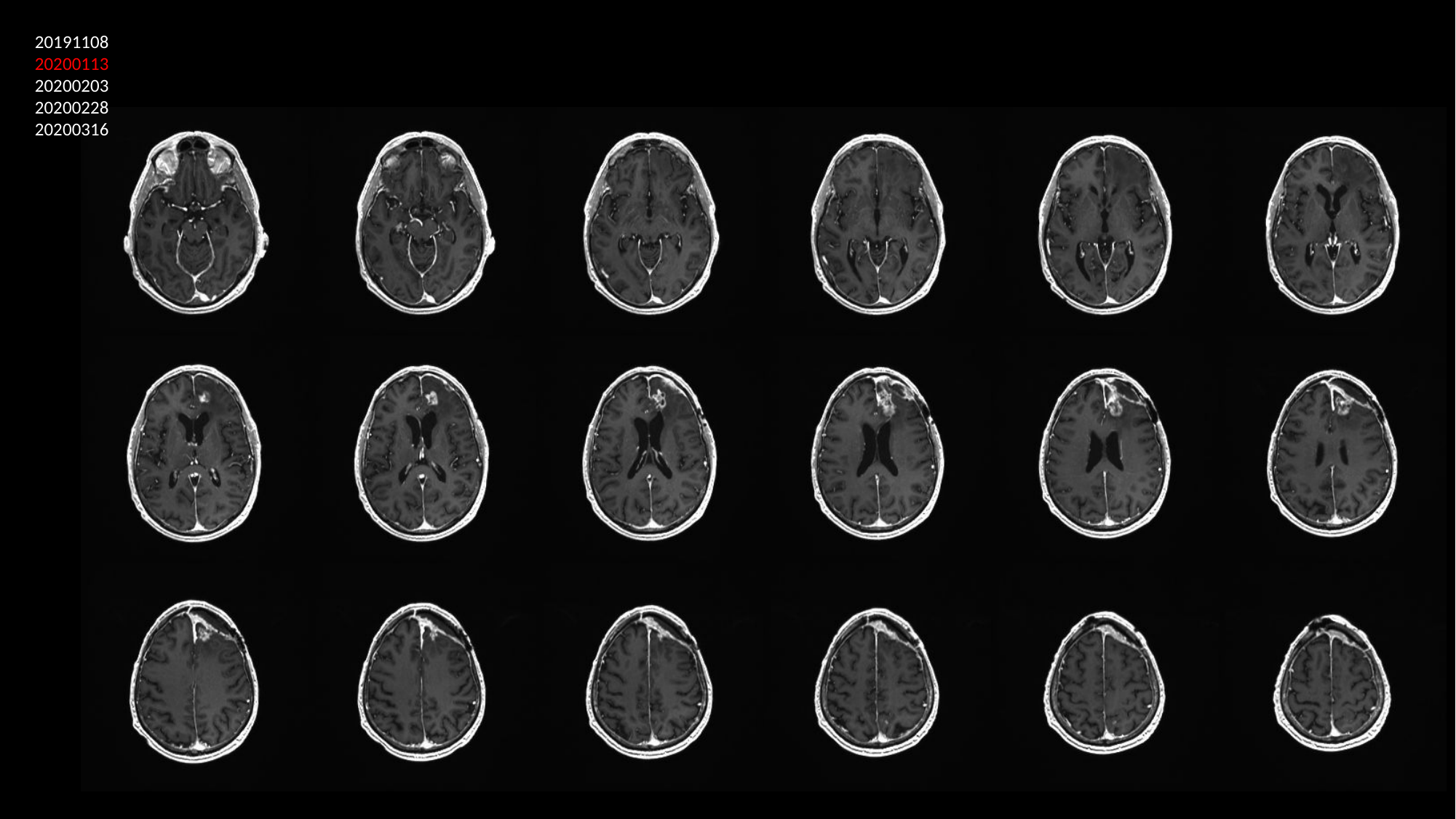

20191108
20200113
20200203
20200228
20200316

## Slide 13
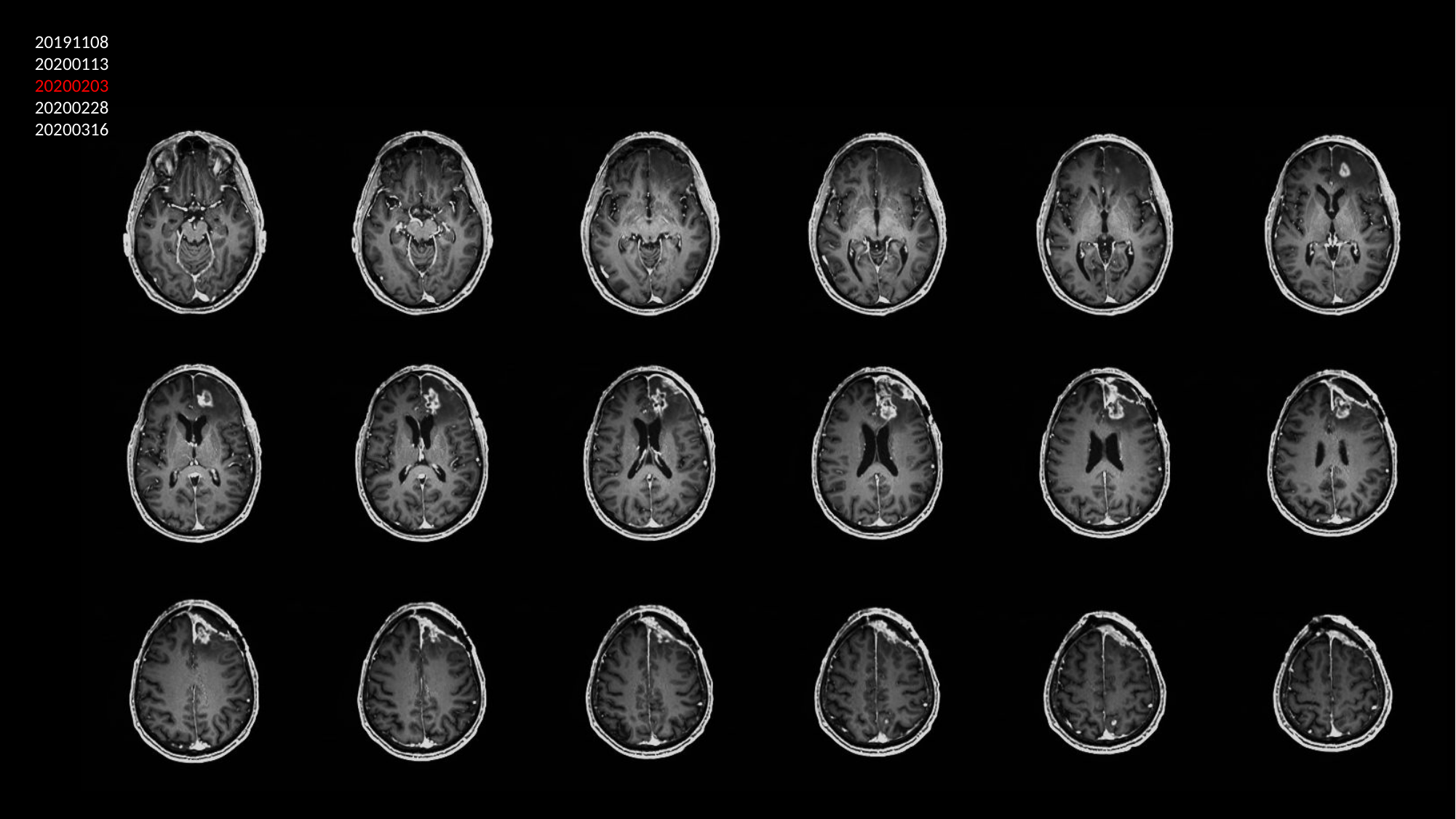

20191108
20200113
20200203
20200228
20200316

## Slide 14
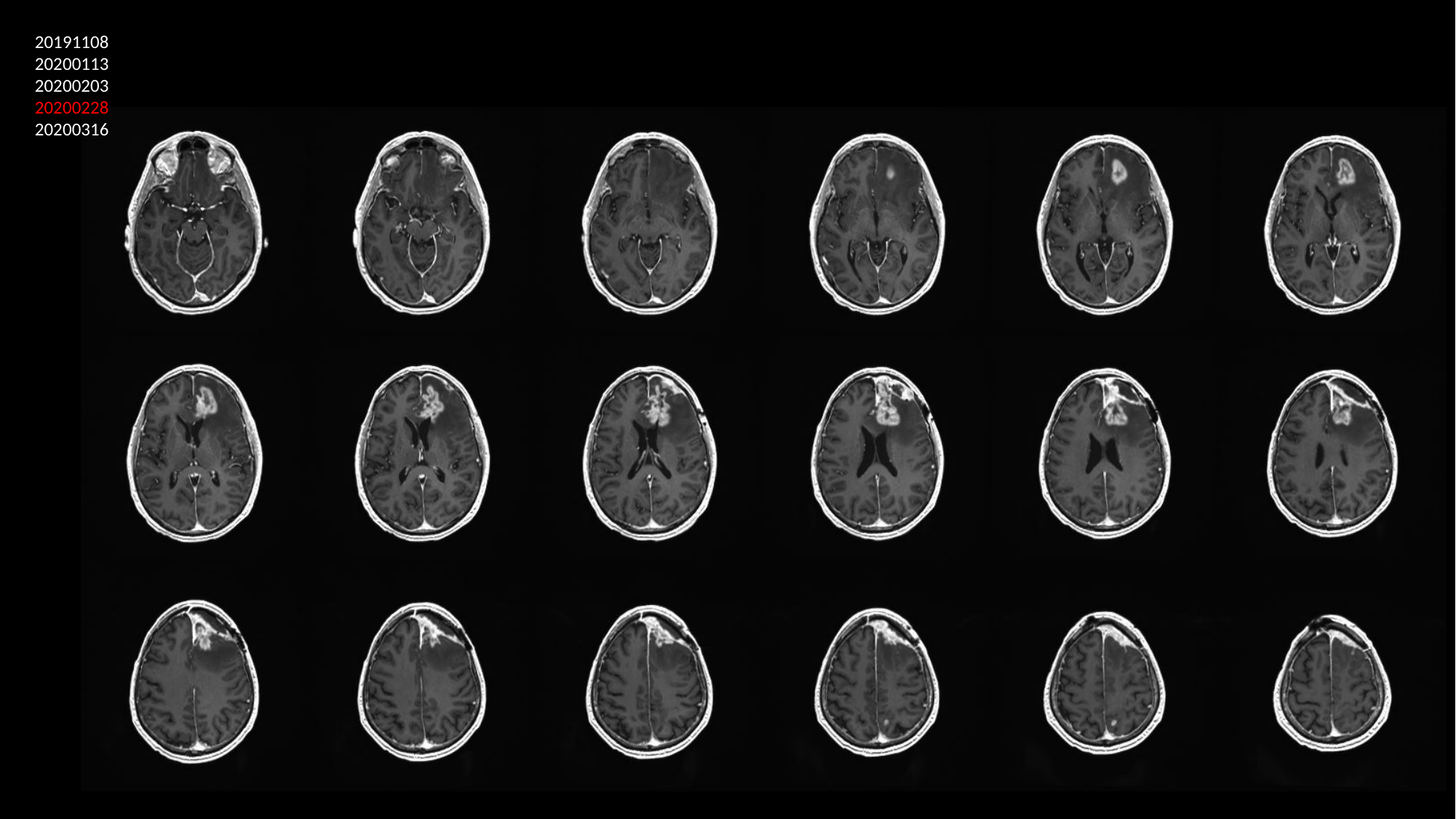

20191108
20200113
20200203
20200228
20200316

## Slide 15
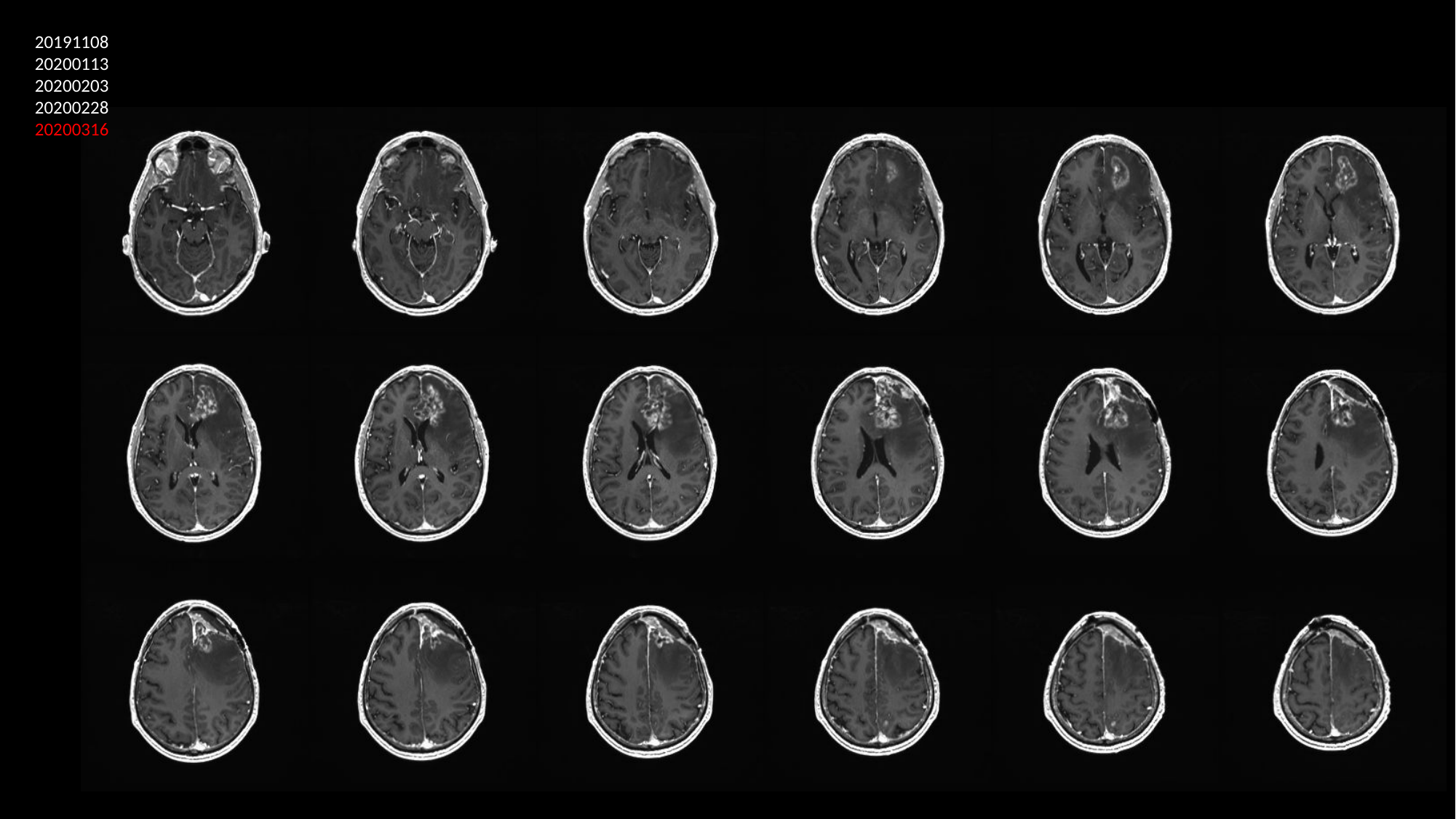

20191108
20200113
20200203
20200228
20200316

## Slide 16
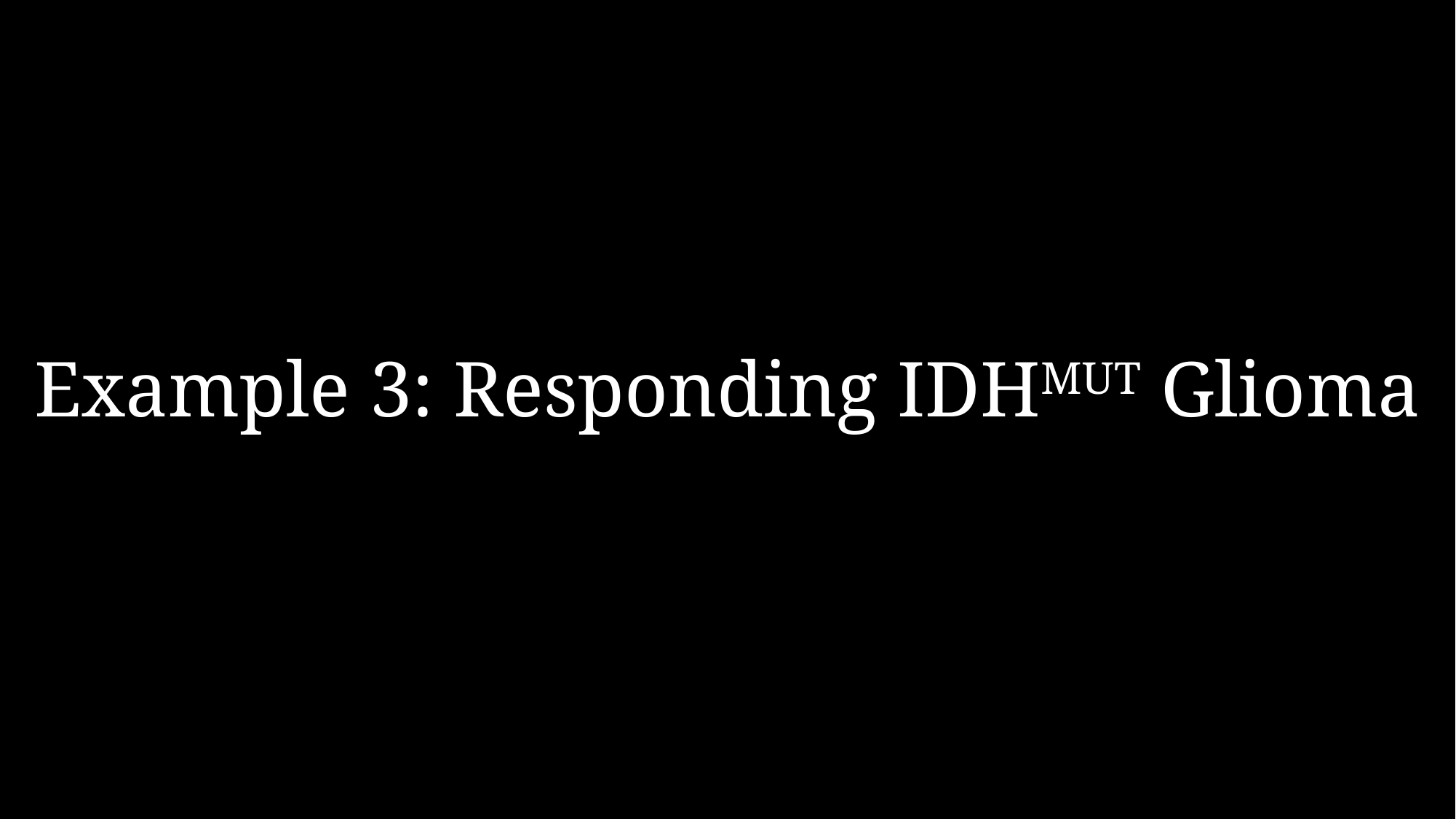

Example 3: Responding IDHMUT Glioma

## Slide 17
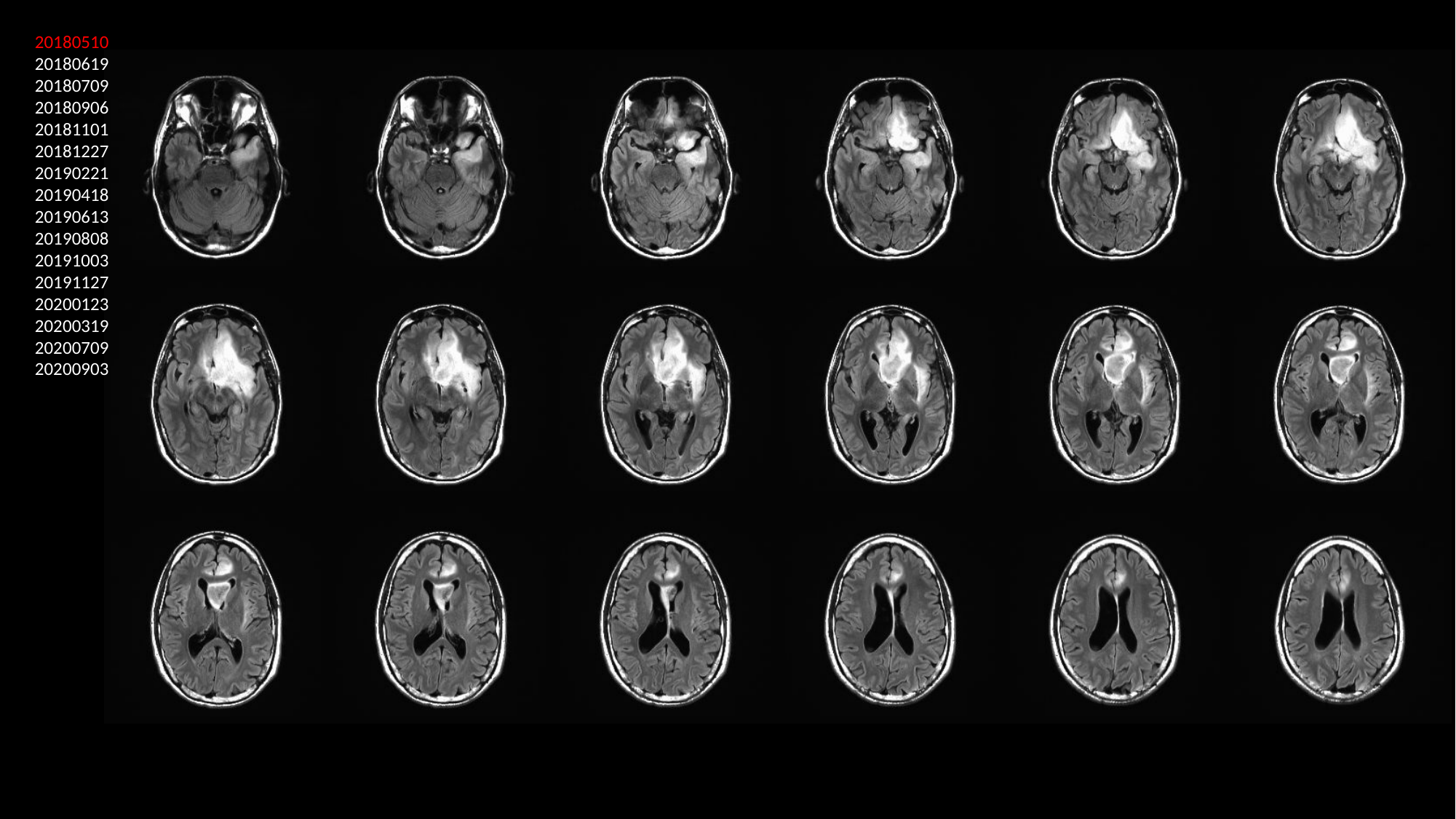

20180510
20180619
20180709
20180906
20181101
20181227
20190221
20190418
20190613
20190808
20191003
20191127
20200123
20200319
20200709
20200903

## Slide 18
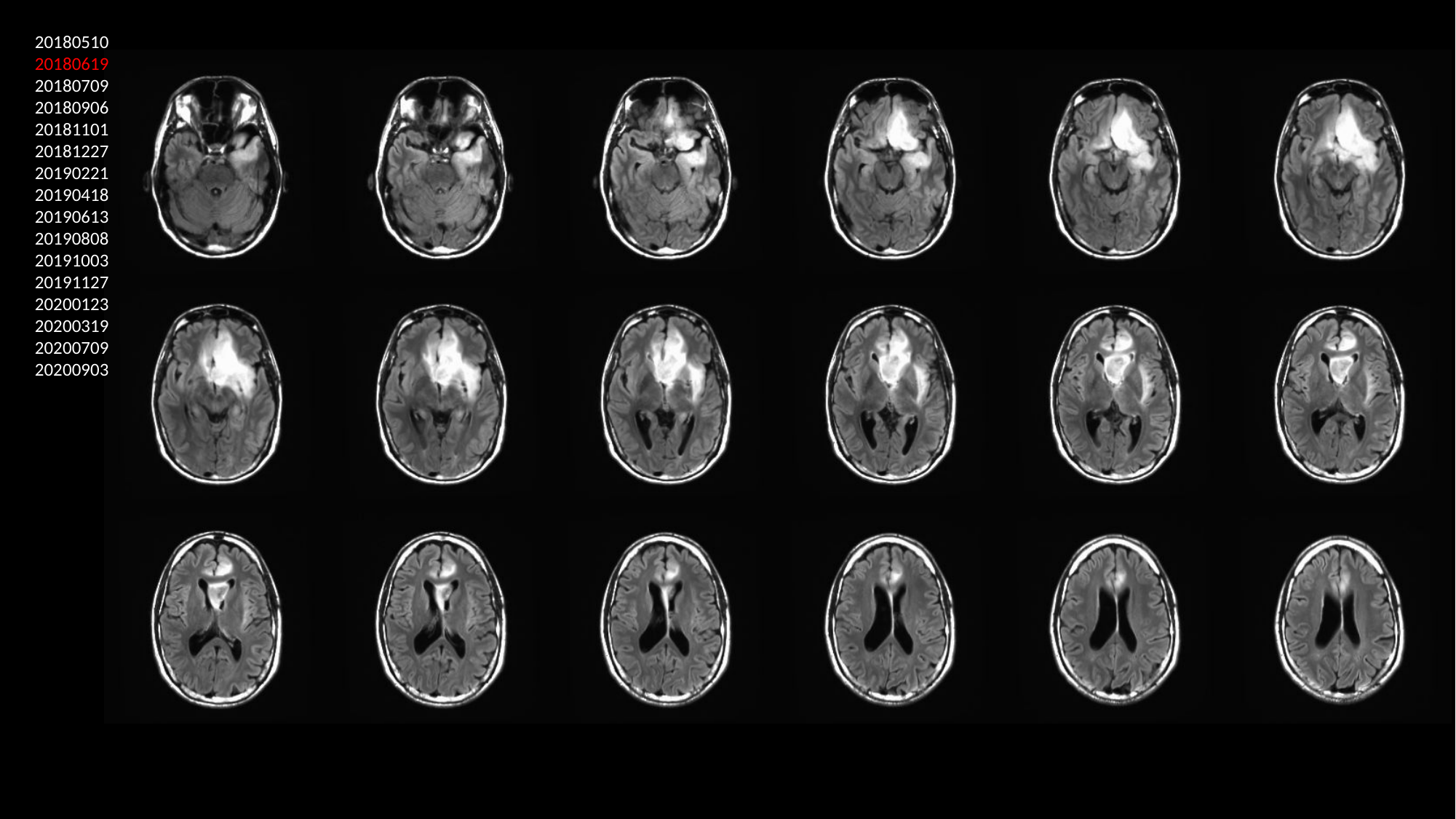

20180510
20180619
20180709
20180906
20181101
20181227
20190221
20190418
20190613
20190808
20191003
20191127
20200123
20200319
20200709
20200903

## Slide 19
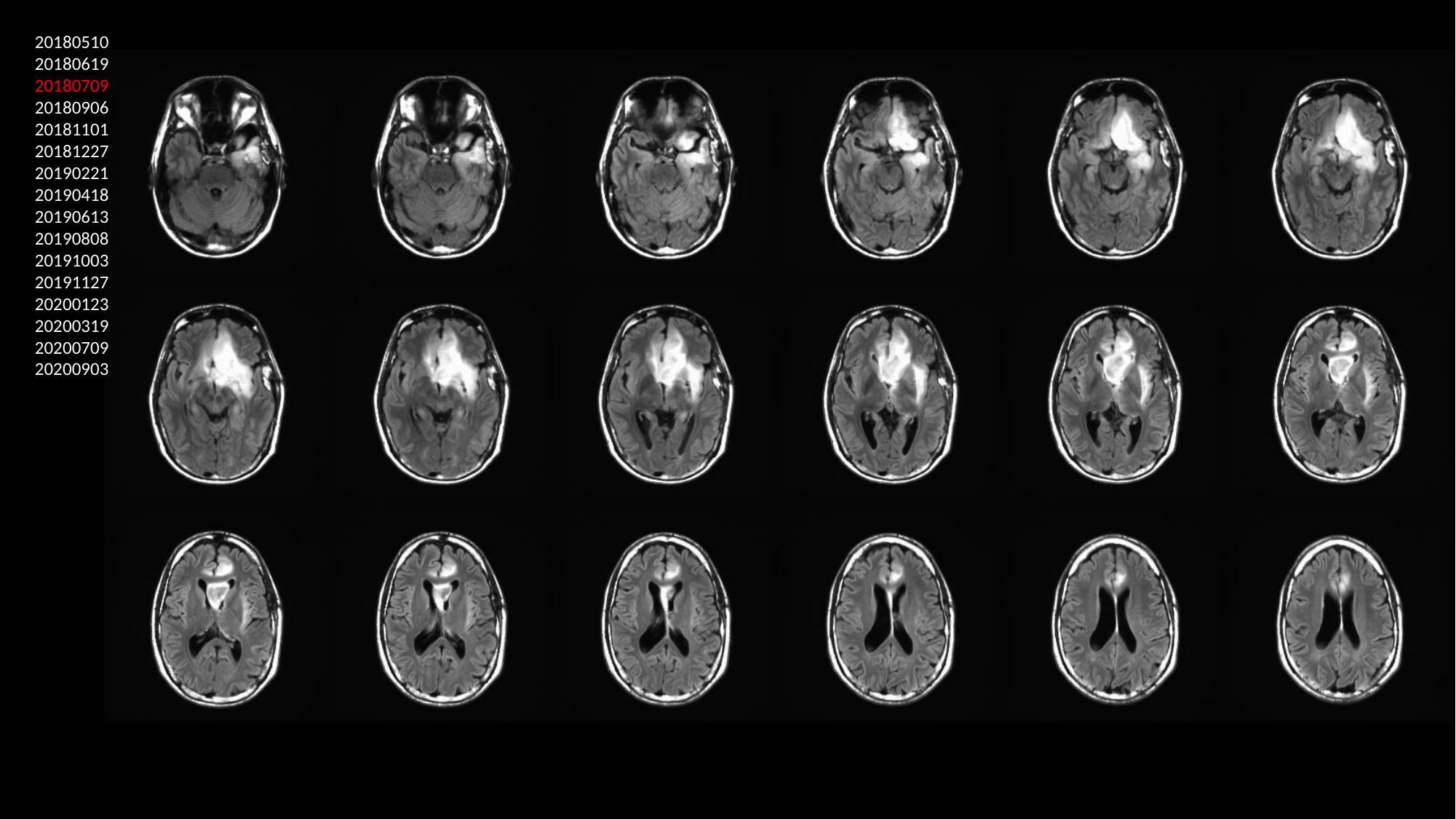

20180510
20180619
20180709
20180906
20181101
20181227
20190221
20190418
20190613
20190808
20191003
20191127
20200123
20200319
20200709
20200903

## Slide 20
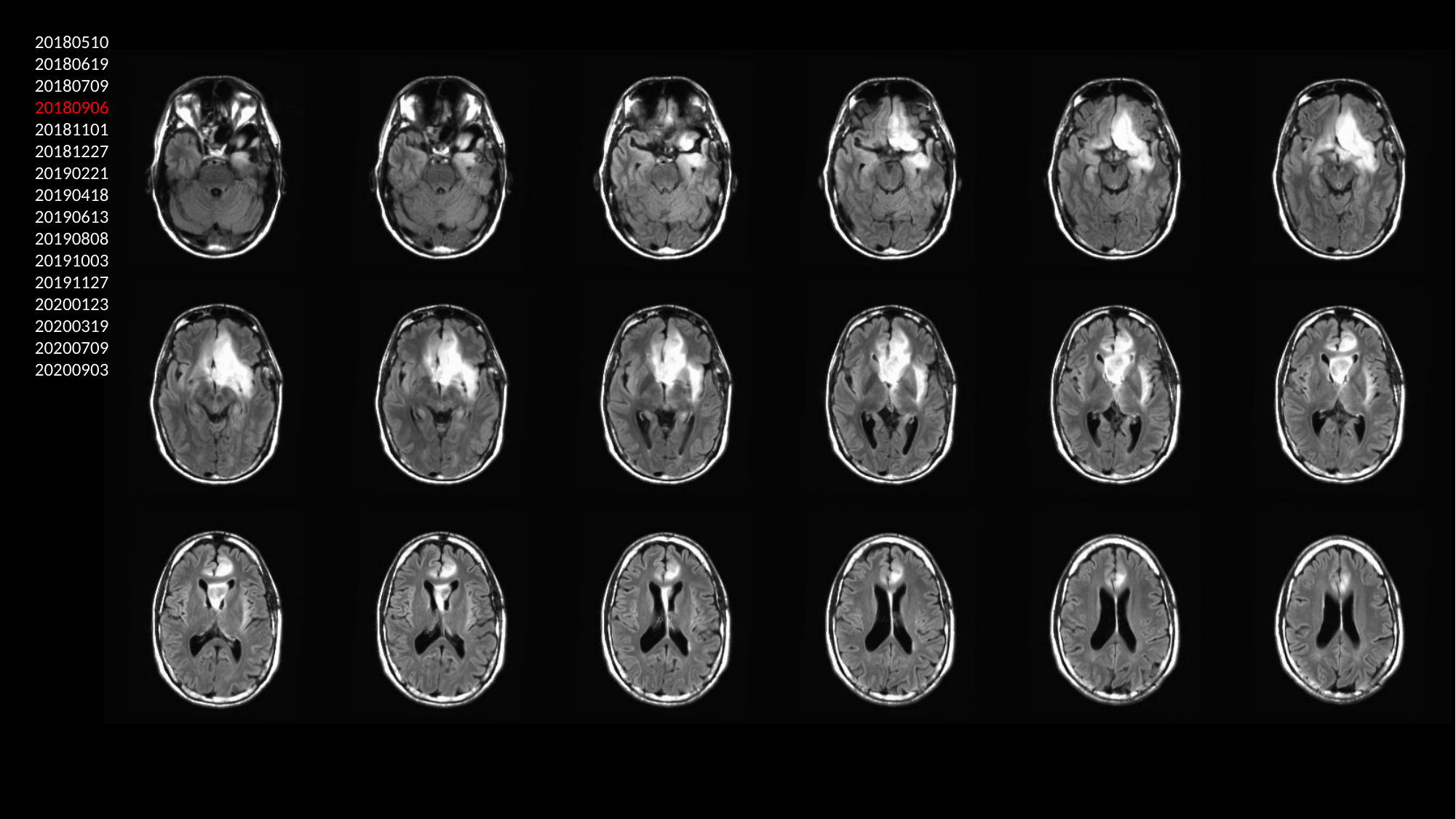

20180510
20180619
20180709
20180906
20181101
20181227
20190221
20190418
20190613
20190808
20191003
20191127
20200123
20200319
20200709
20200903

## Slide 21
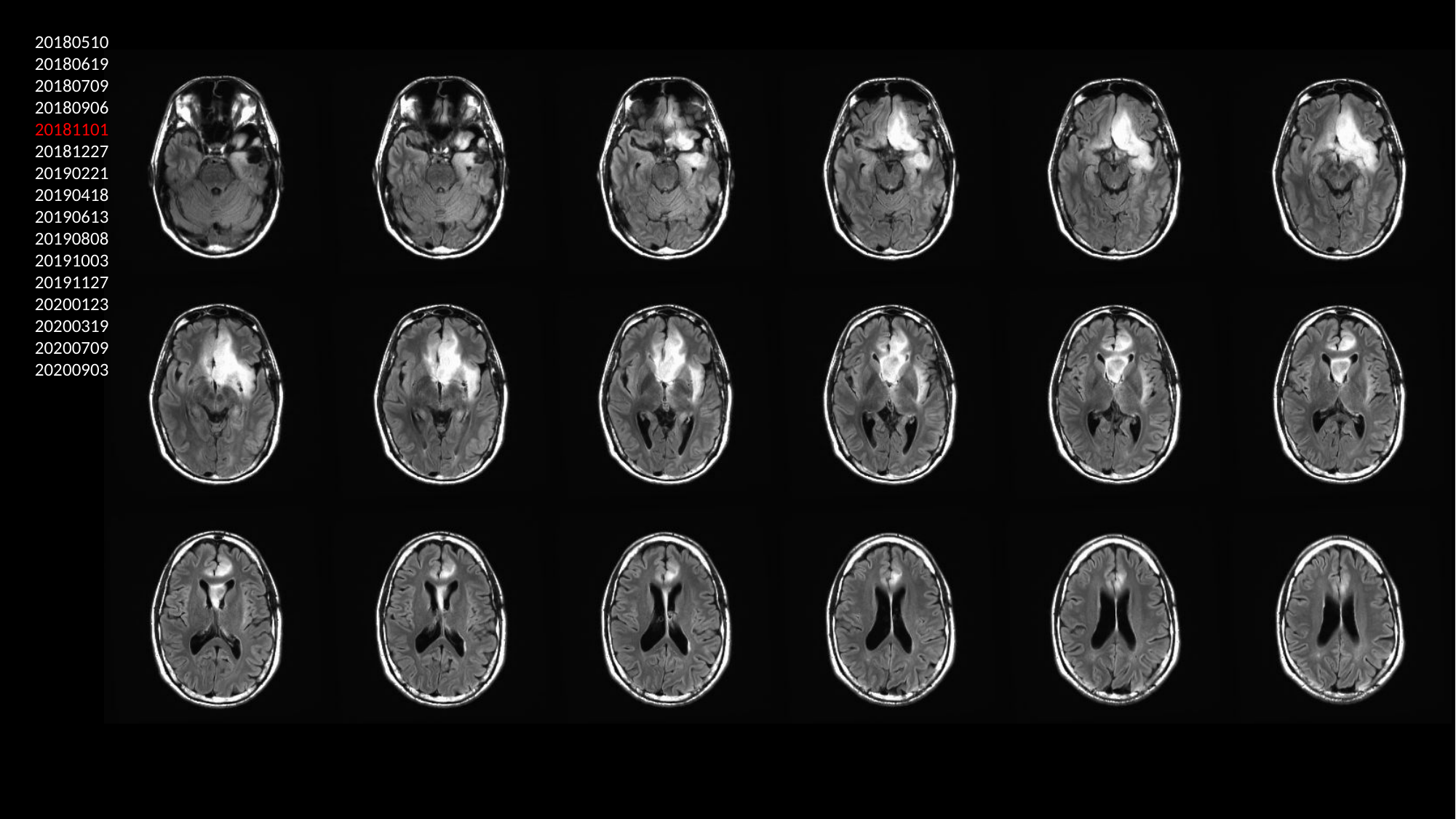

20180510
20180619
20180709
20180906
20181101
20181227
20190221
20190418
20190613
20190808
20191003
20191127
20200123
20200319
20200709
20200903

## Slide 22
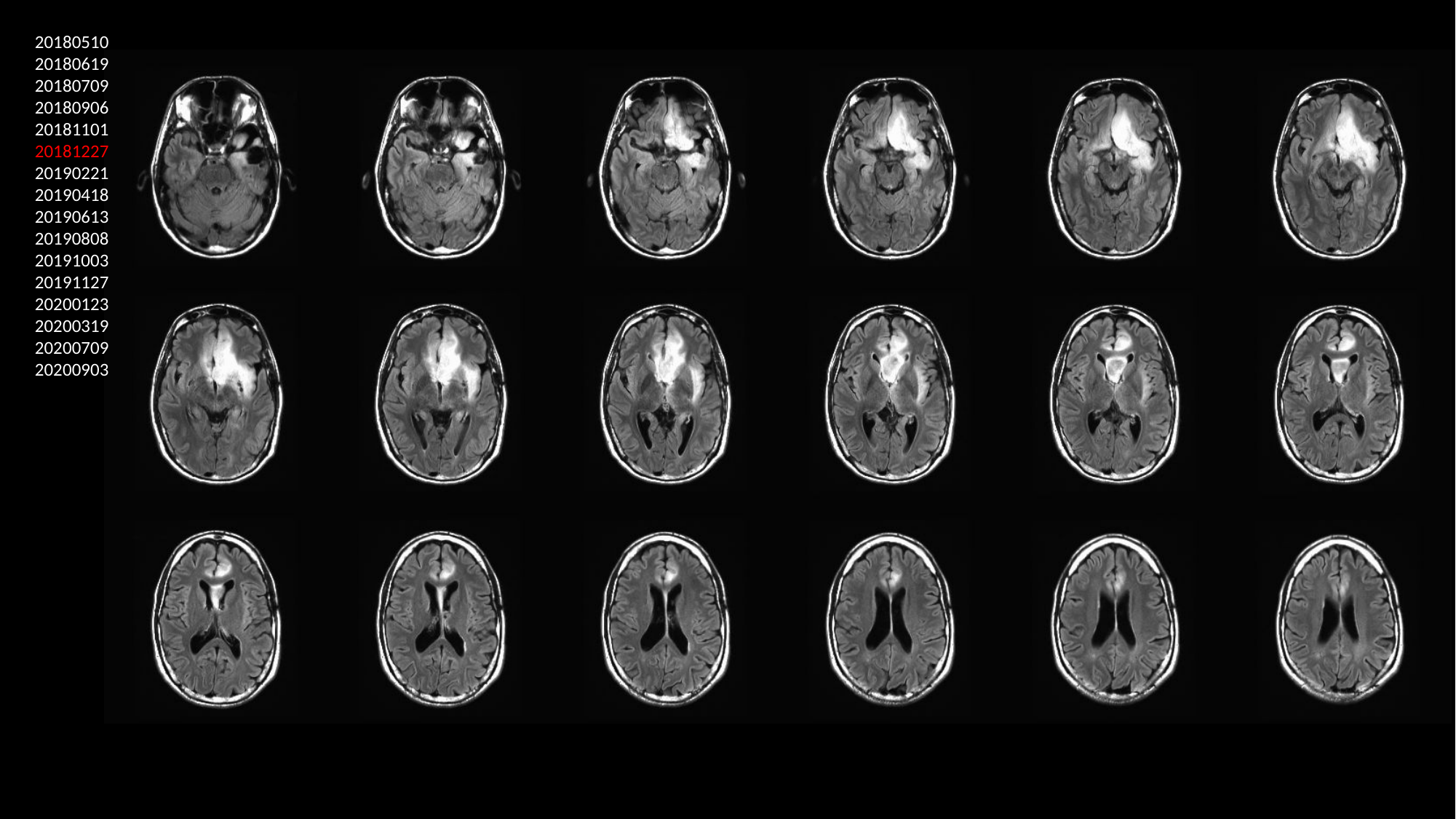

20180510
20180619
20180709
20180906
20181101
20181227
20190221
20190418
20190613
20190808
20191003
20191127
20200123
20200319
20200709
20200903

## Slide 23
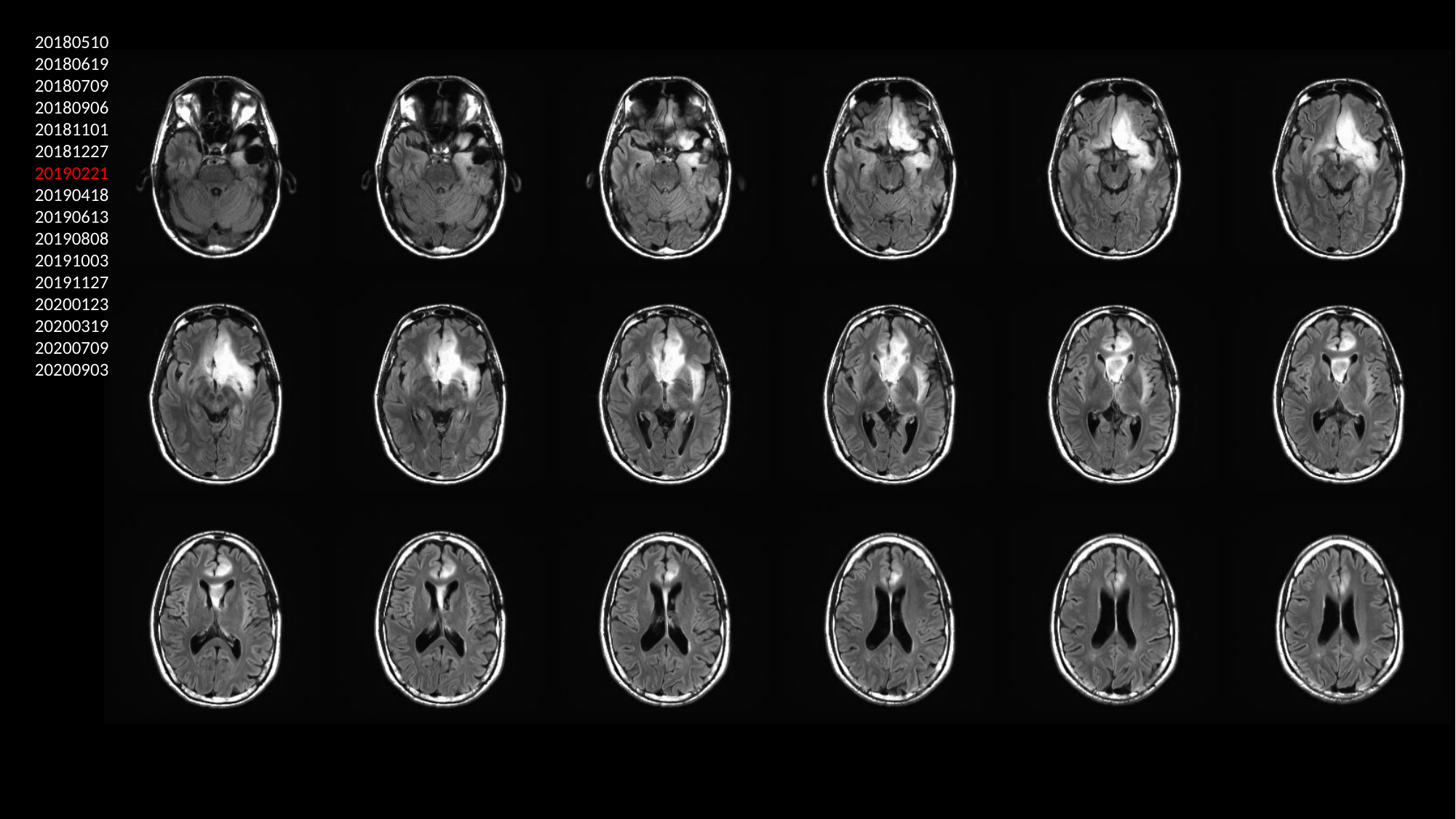

20180510
20180619
20180709
20180906
20181101
20181227
20190221
20190418
20190613
20190808
20191003
20191127
20200123
20200319
20200709
20200903

## Slide 24
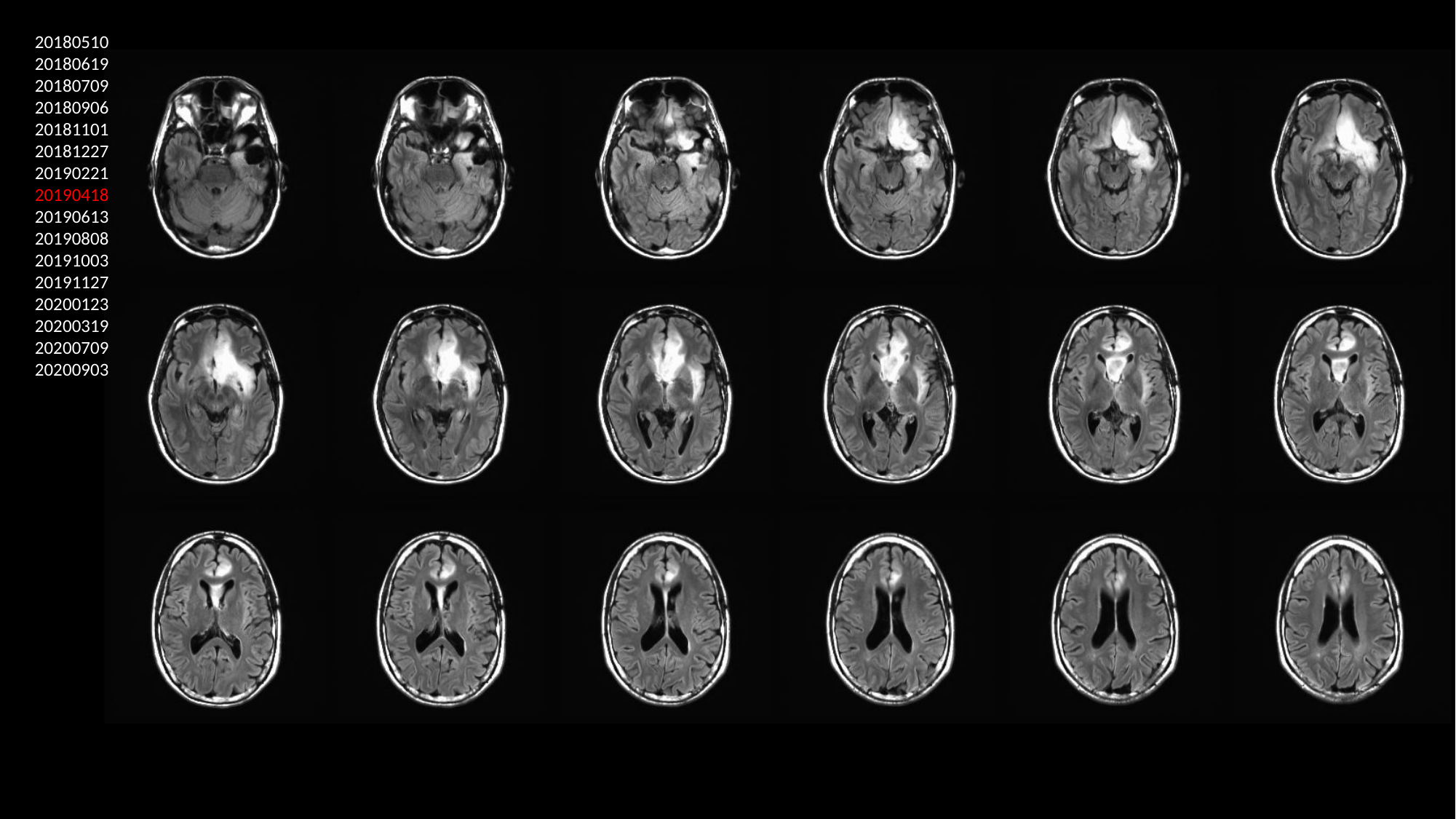

20180510
20180619
20180709
20180906
20181101
20181227
20190221
20190418
20190613
20190808
20191003
20191127
20200123
20200319
20200709
20200903

## Slide 25
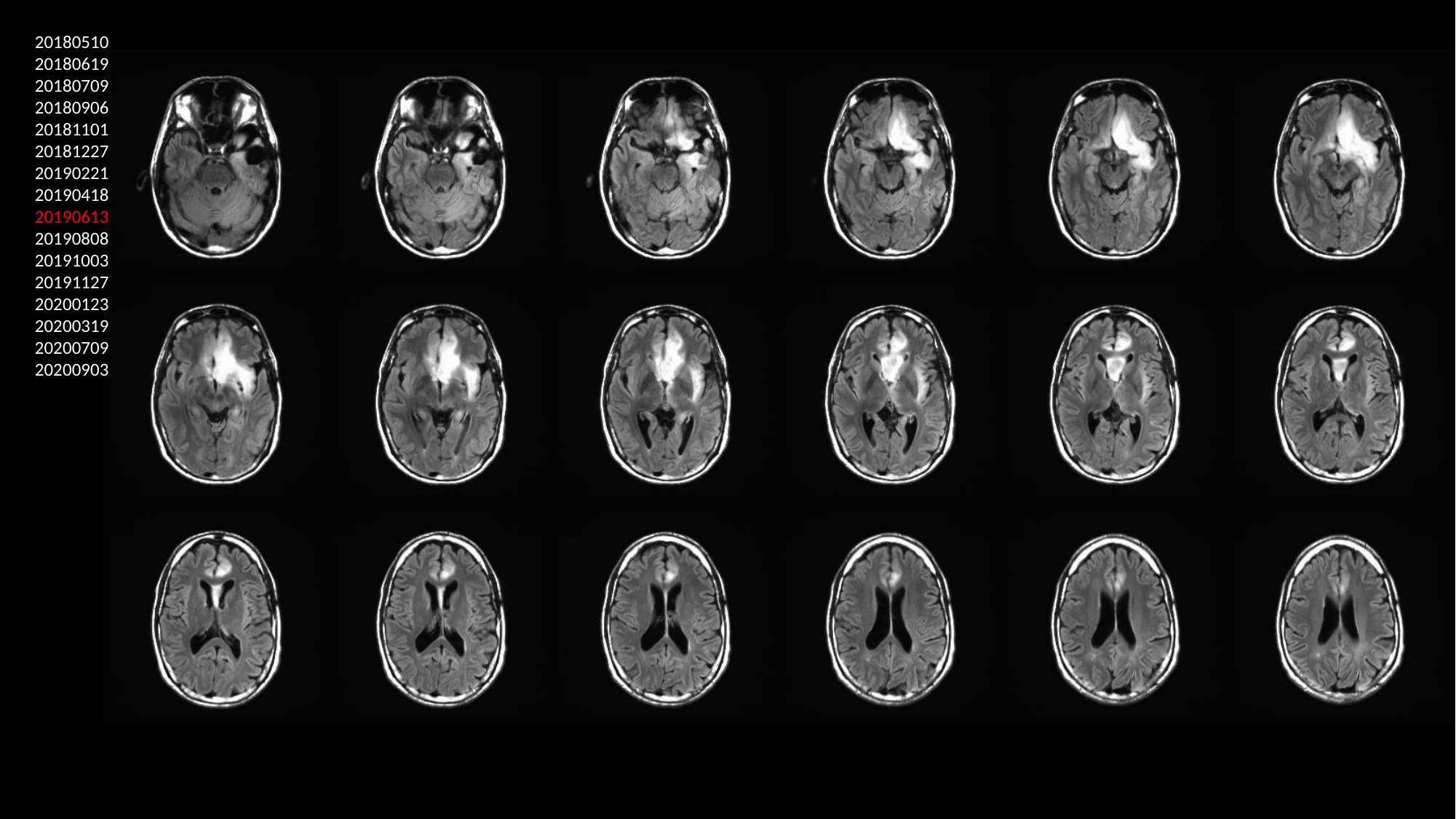

20180510
20180619
20180709
20180906
20181101
20181227
20190221
20190418
20190613
20190808
20191003
20191127
20200123
20200319
20200709
20200903

## Slide 26
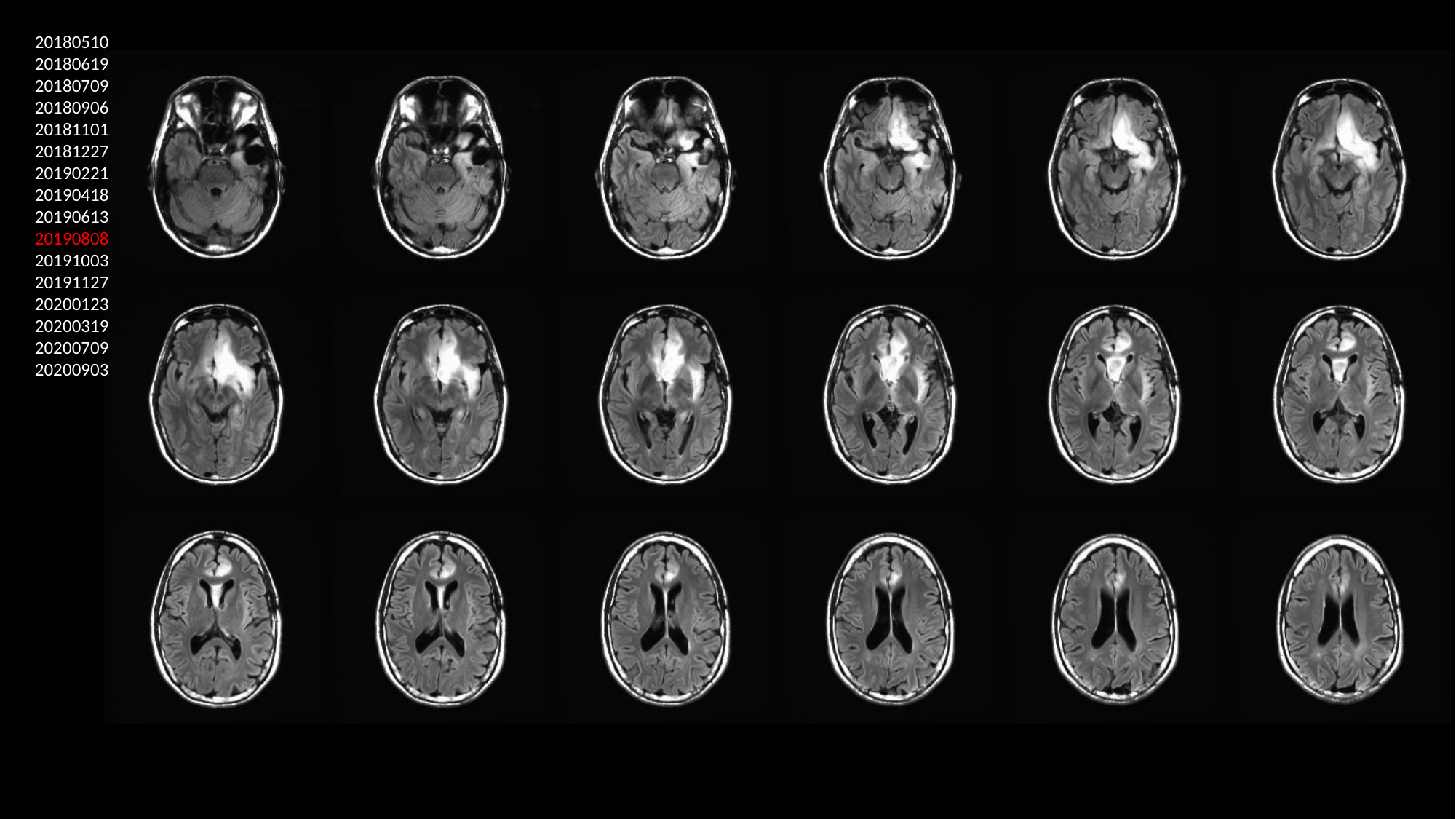

20180510
20180619
20180709
20180906
20181101
20181227
20190221
20190418
20190613
20190808
20191003
20191127
20200123
20200319
20200709
20200903

## Slide 27
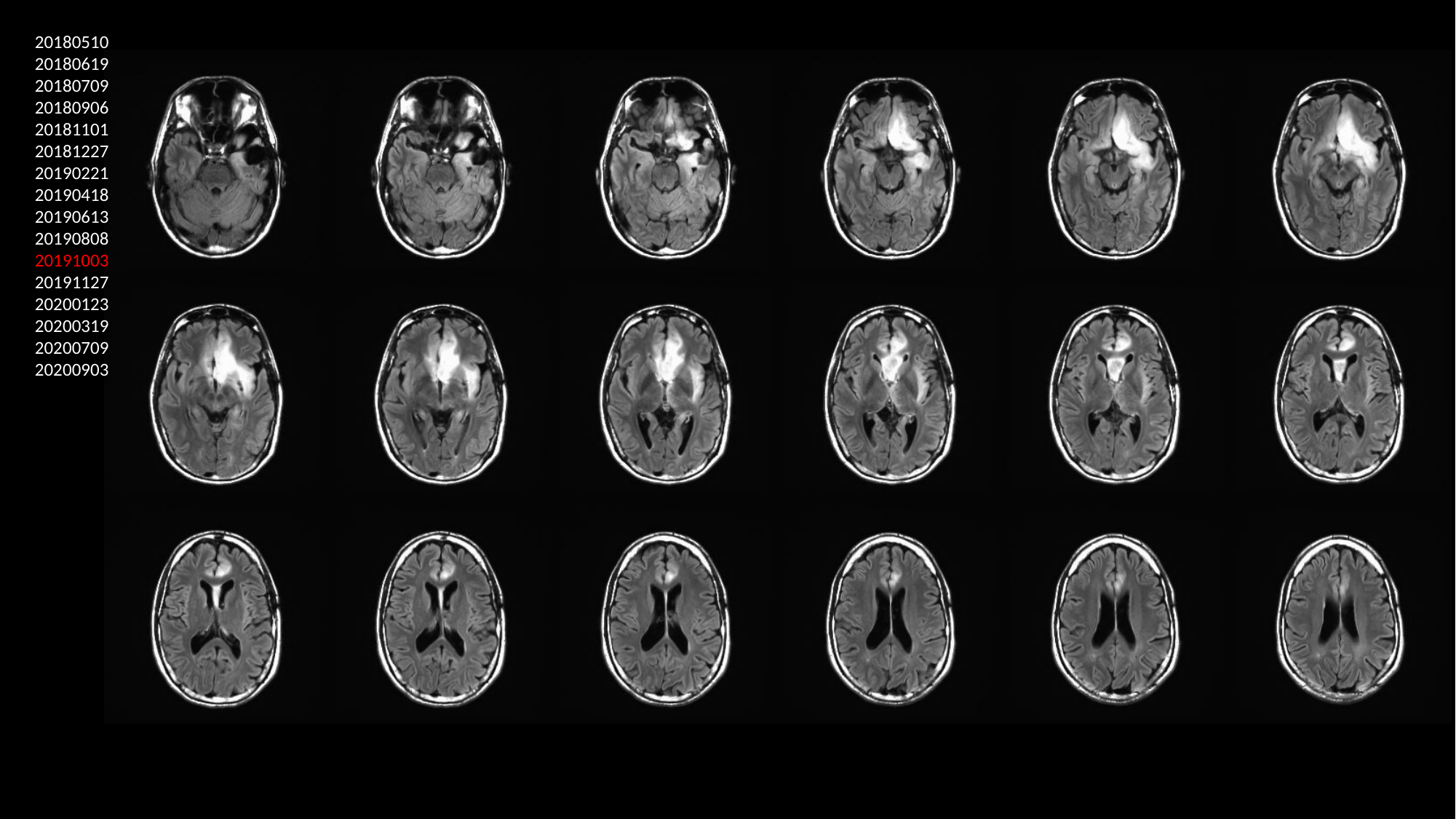

20180510
20180619
20180709
20180906
20181101
20181227
20190221
20190418
20190613
20190808
20191003
20191127
20200123
20200319
20200709
20200903

## Slide 28
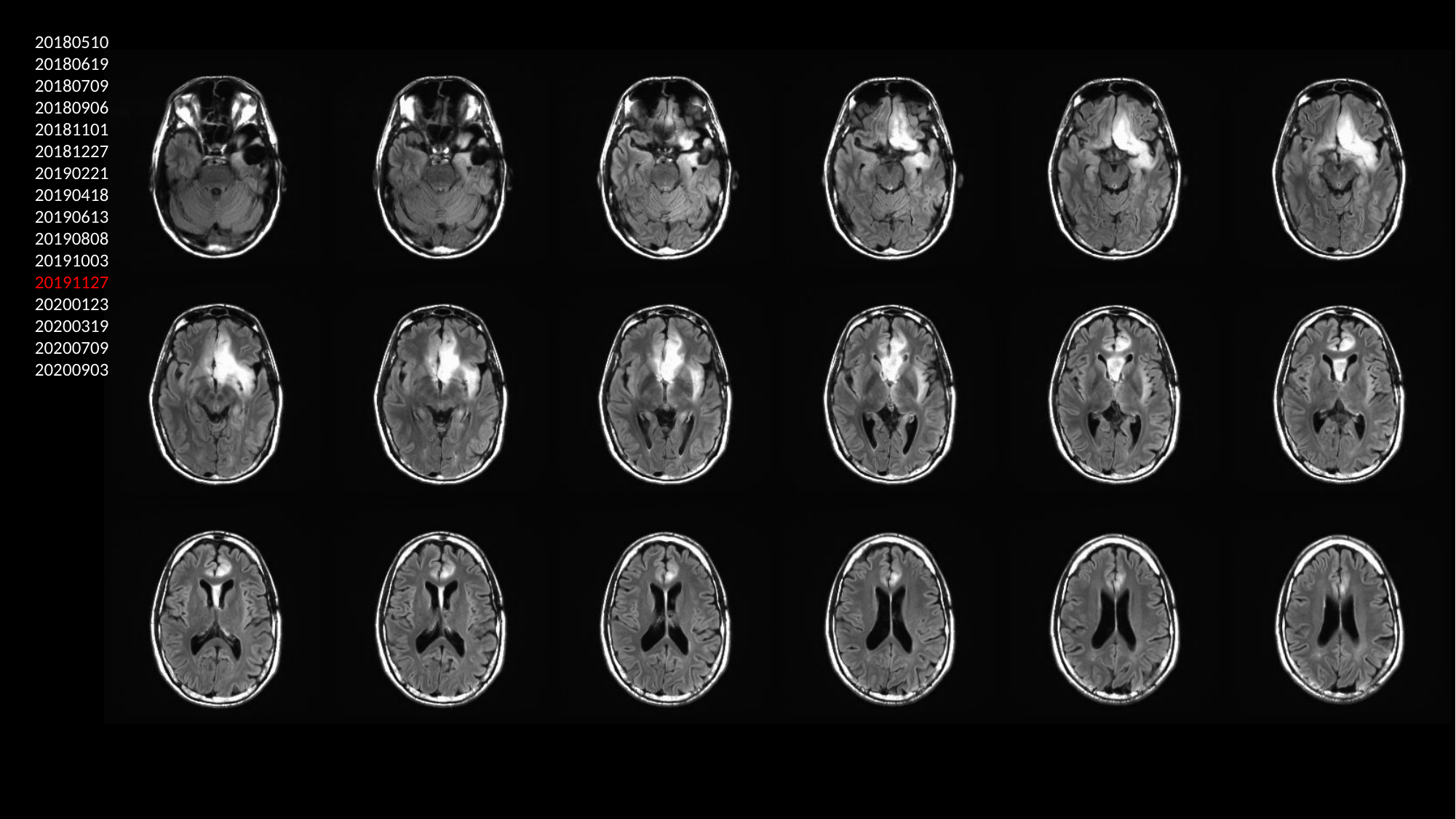

20180510
20180619
20180709
20180906
20181101
20181227
20190221
20190418
20190613
20190808
20191003
20191127
20200123
20200319
20200709
20200903

## Slide 29
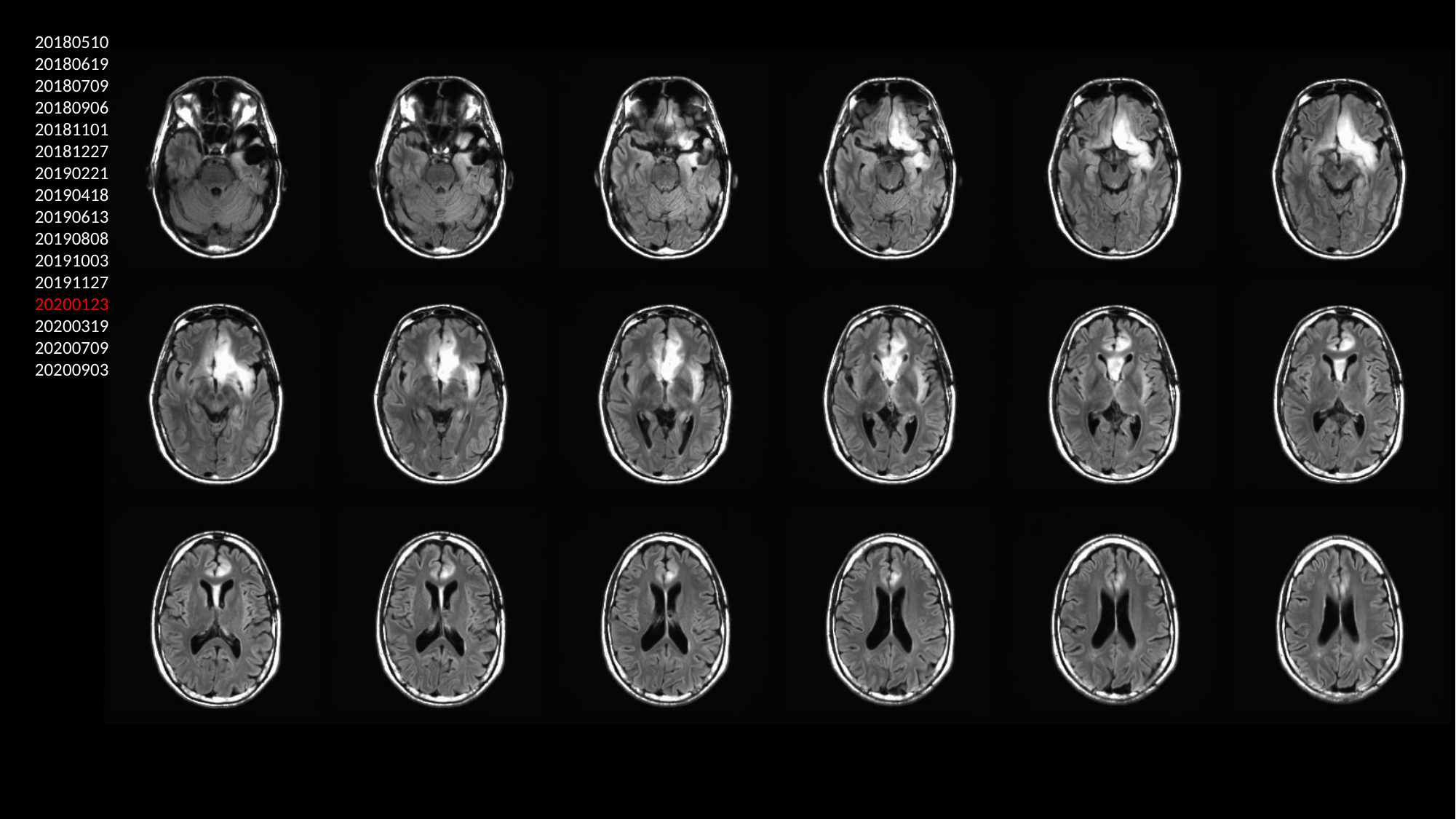

20180510
20180619
20180709
20180906
20181101
20181227
20190221
20190418
20190613
20190808
20191003
20191127
20200123
20200319
20200709
20200903

## Slide 30
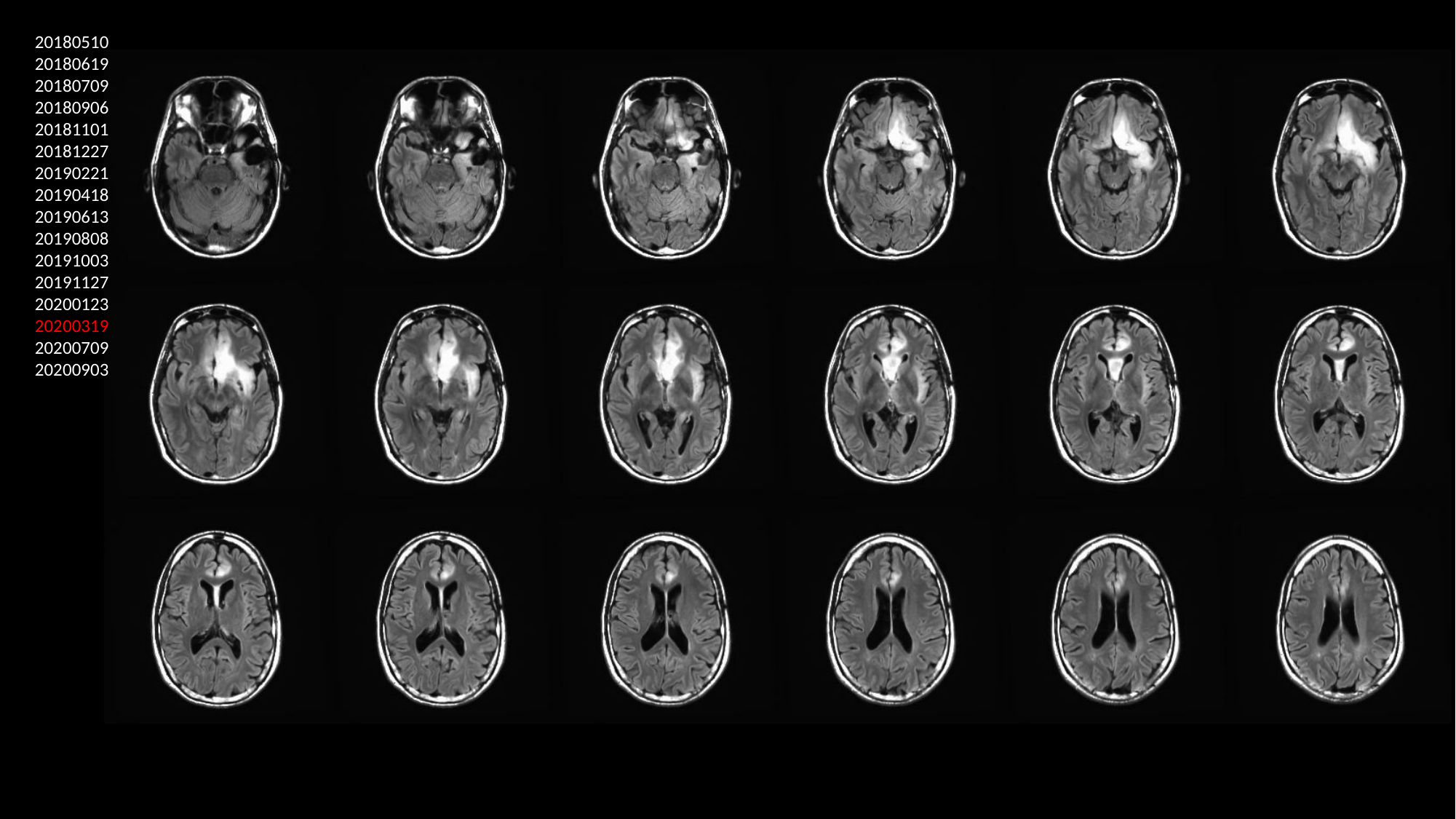

20180510
20180619
20180709
20180906
20181101
20181227
20190221
20190418
20190613
20190808
20191003
20191127
20200123
20200319
20200709
20200903

## Slide 31
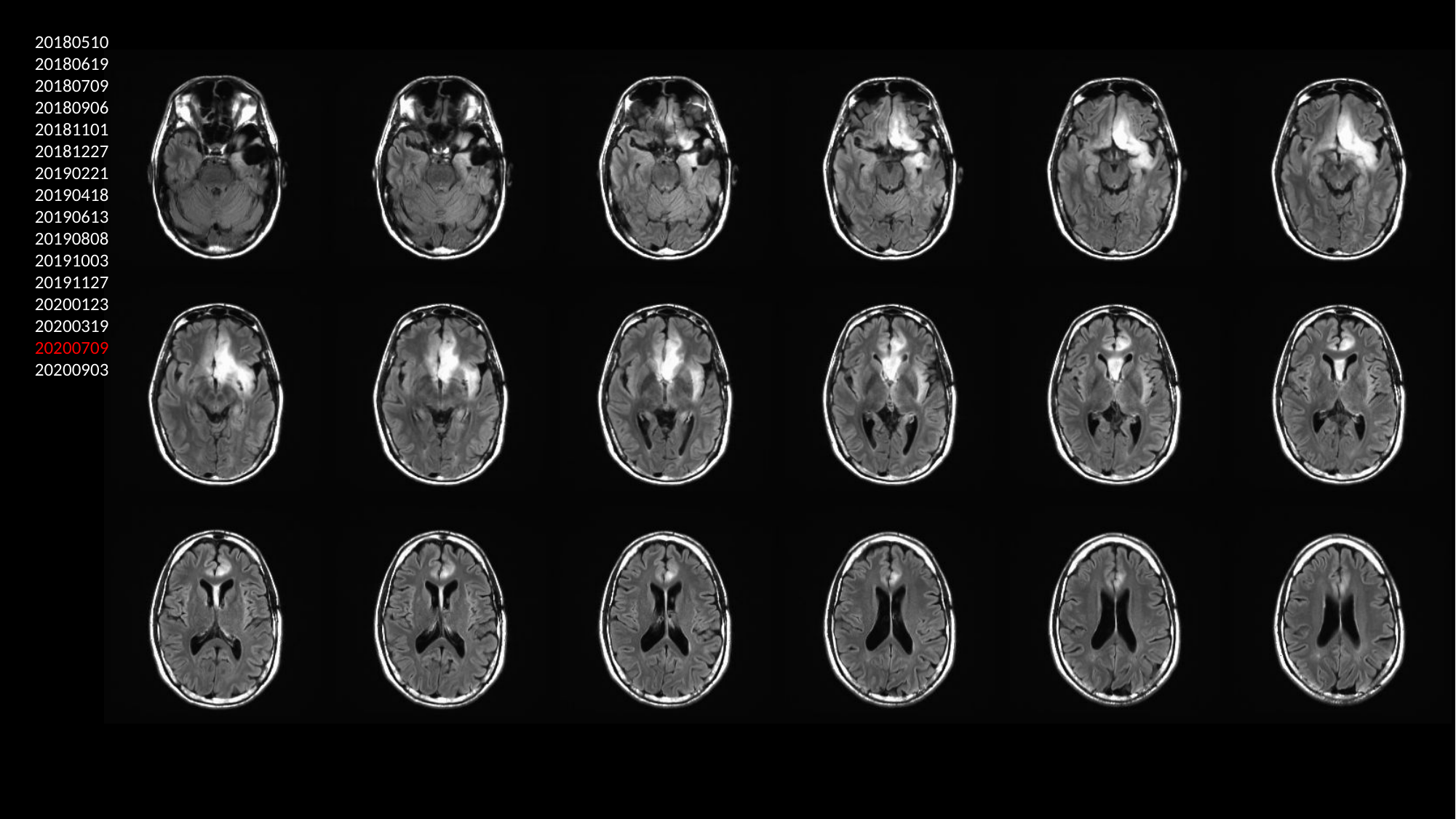

20180510
20180619
20180709
20180906
20181101
20181227
20190221
20190418
20190613
20190808
20191003
20191127
20200123
20200319
20200709
20200903

## Slide 32
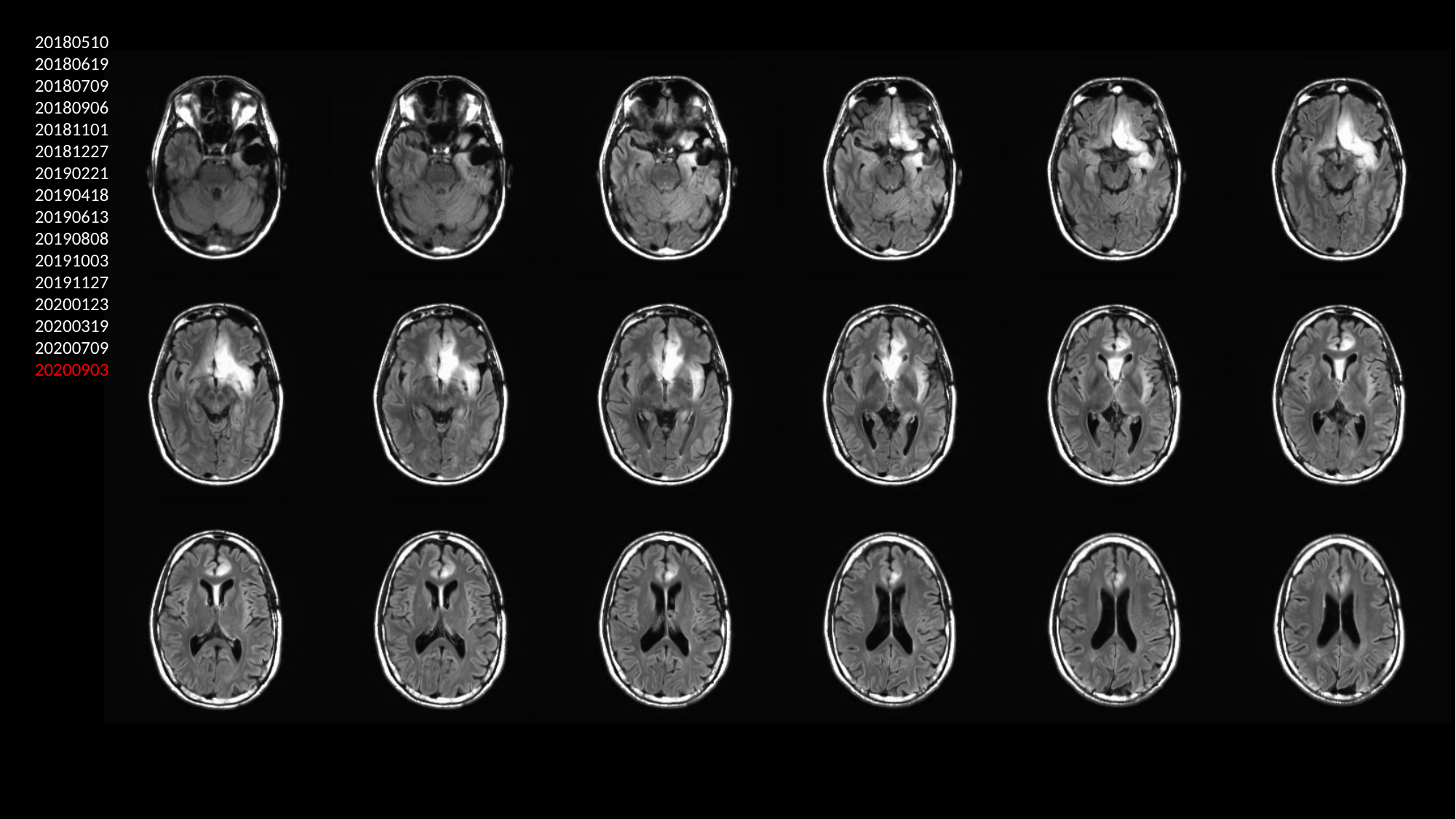

20180510
20180619
20180709
20180906
20181101
20181227
20190221
20190418
20190613
20190808
20191003
20191127
20200123
20200319
20200709
20200903

## Slide 33
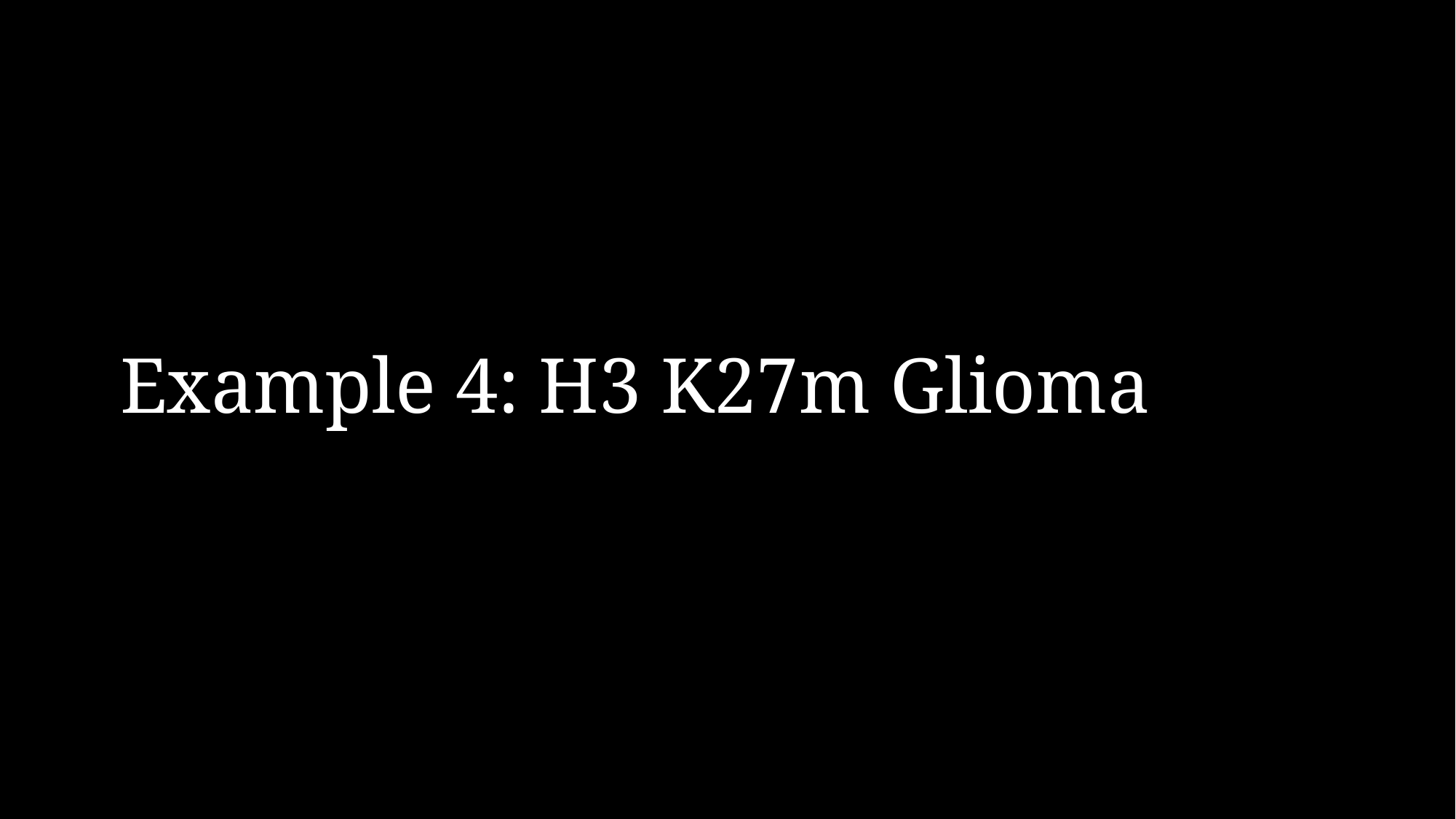

Example 4: H3 K27m Glioma

## Slide 34
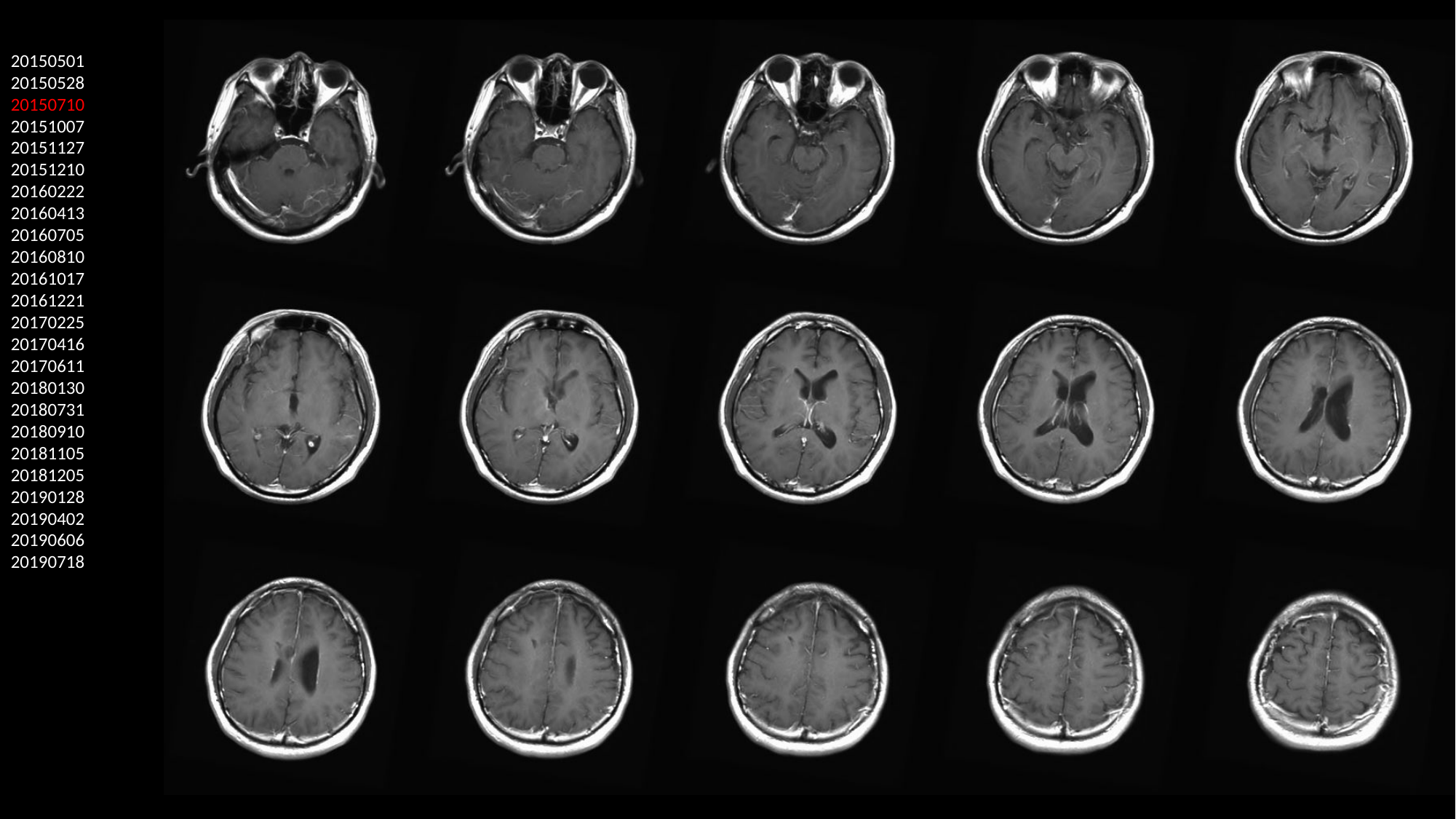

20150501
20150528
20150710
20151007
20151127
20151210
20160222
20160413
20160705
20160810
20161017
20161221
20170225
20170416
20170611
20180130
20180731
20180910
20181105
20181205
20190128
20190402
20190606
20190718

## Slide 35
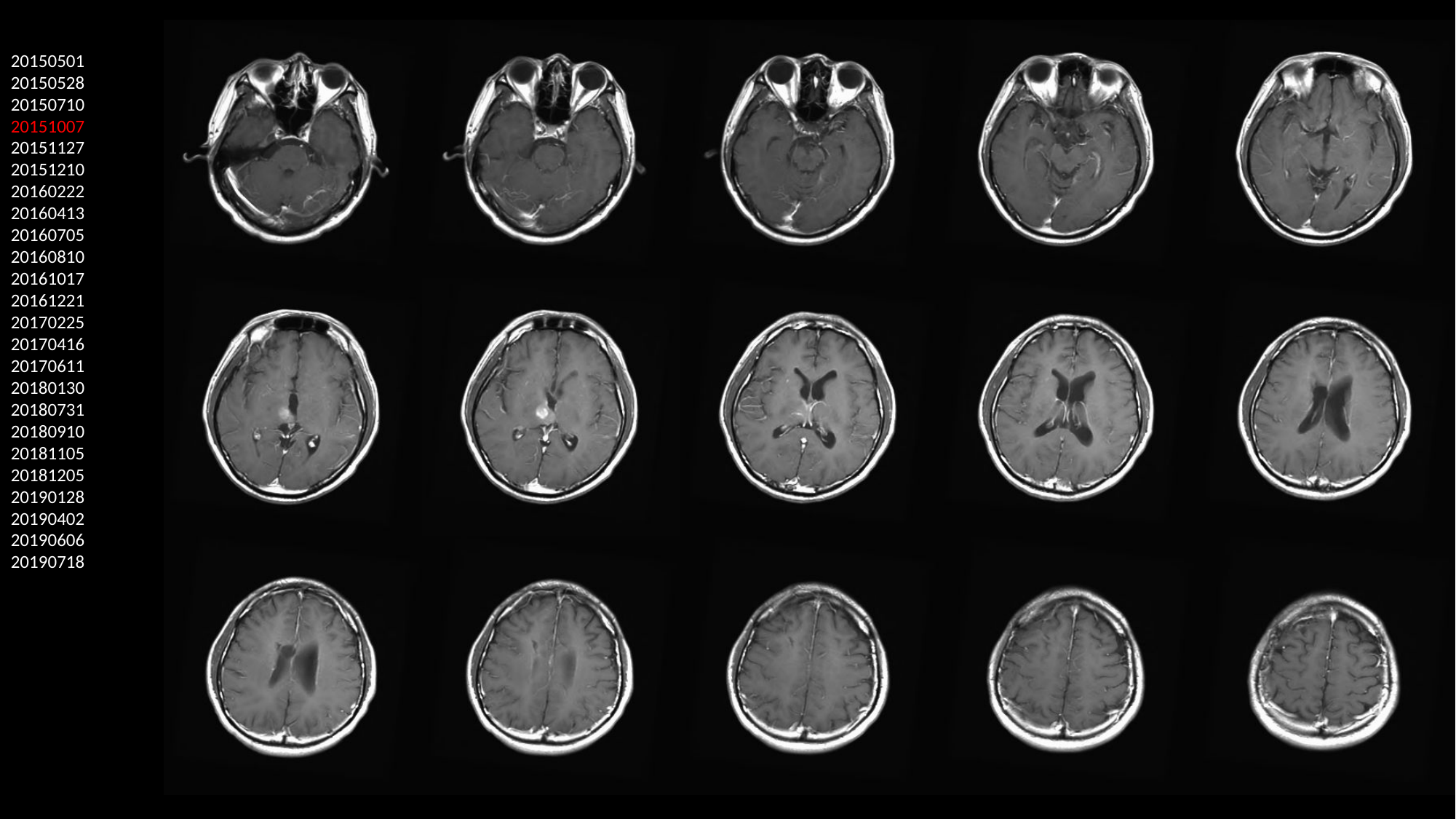

20150501
20150528
20150710
20151007
20151127
20151210
20160222
20160413
20160705
20160810
20161017
20161221
20170225
20170416
20170611
20180130
20180731
20180910
20181105
20181205
20190128
20190402
20190606
20190718

## Slide 36
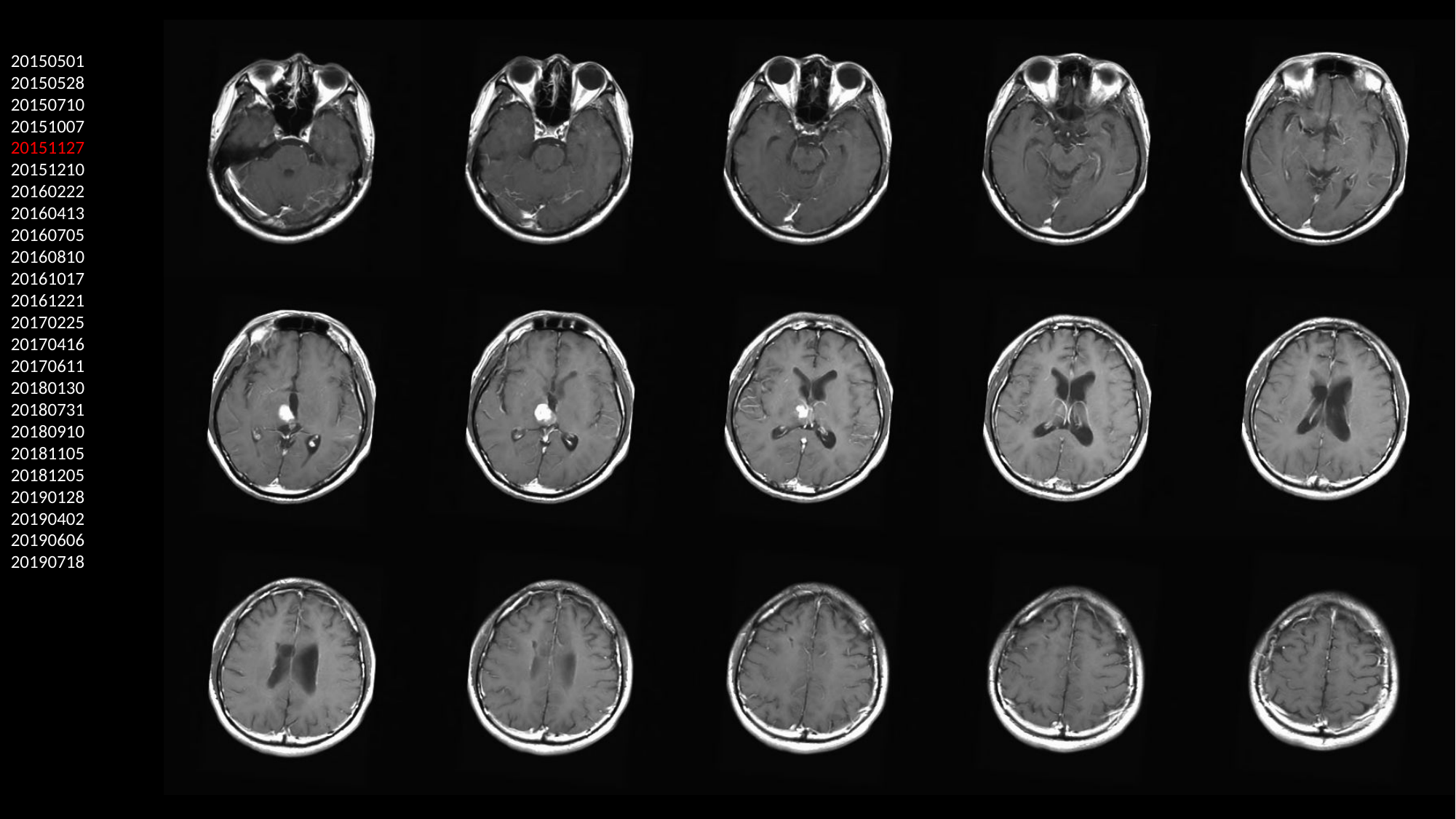

20150501
20150528
20150710
20151007
20151127
20151210
20160222
20160413
20160705
20160810
20161017
20161221
20170225
20170416
20170611
20180130
20180731
20180910
20181105
20181205
20190128
20190402
20190606
20190718

## Slide 37
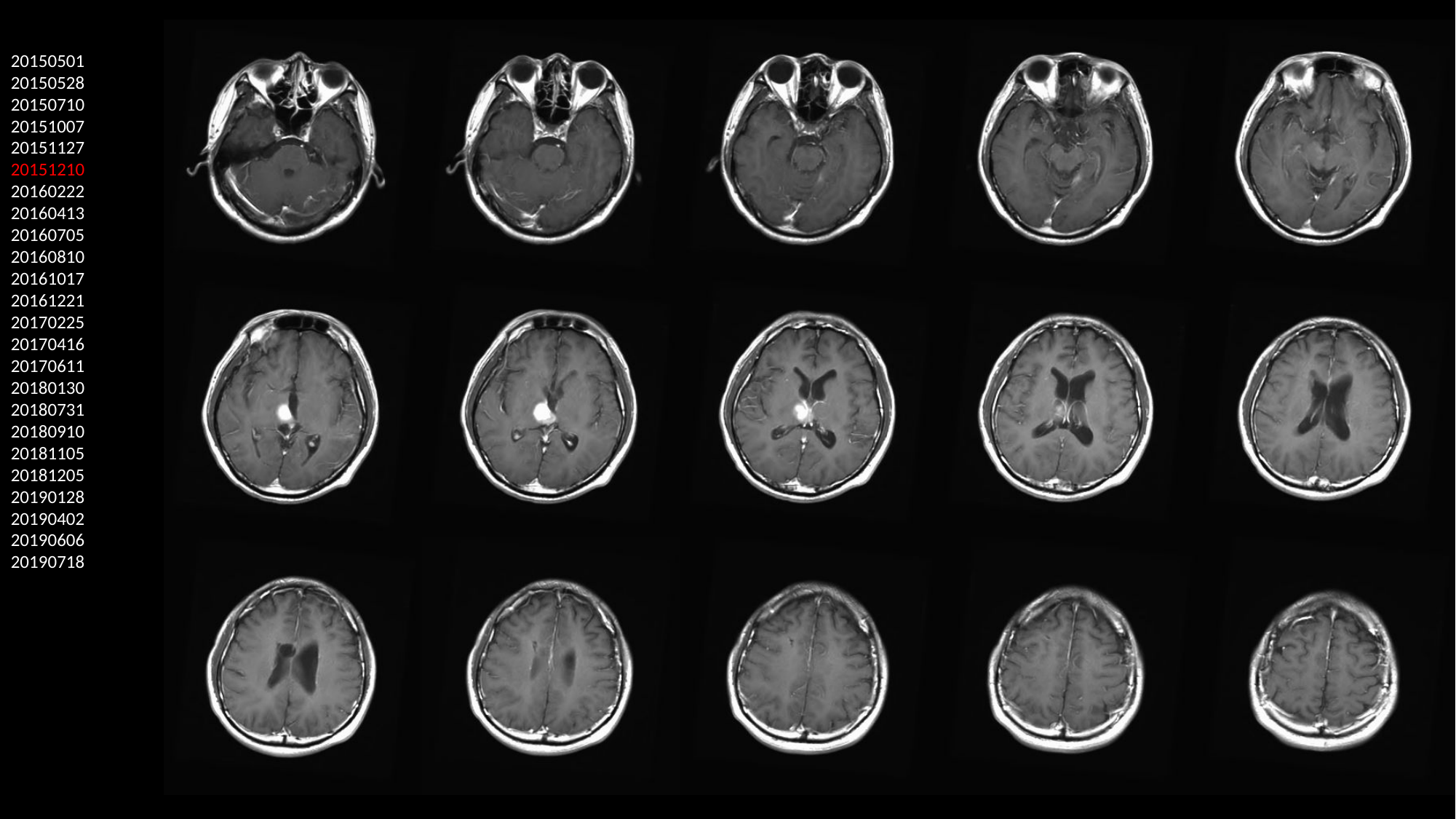

20150501
20150528
20150710
20151007
20151127
20151210
20160222
20160413
20160705
20160810
20161017
20161221
20170225
20170416
20170611
20180130
20180731
20180910
20181105
20181205
20190128
20190402
20190606
20190718

## Slide 38
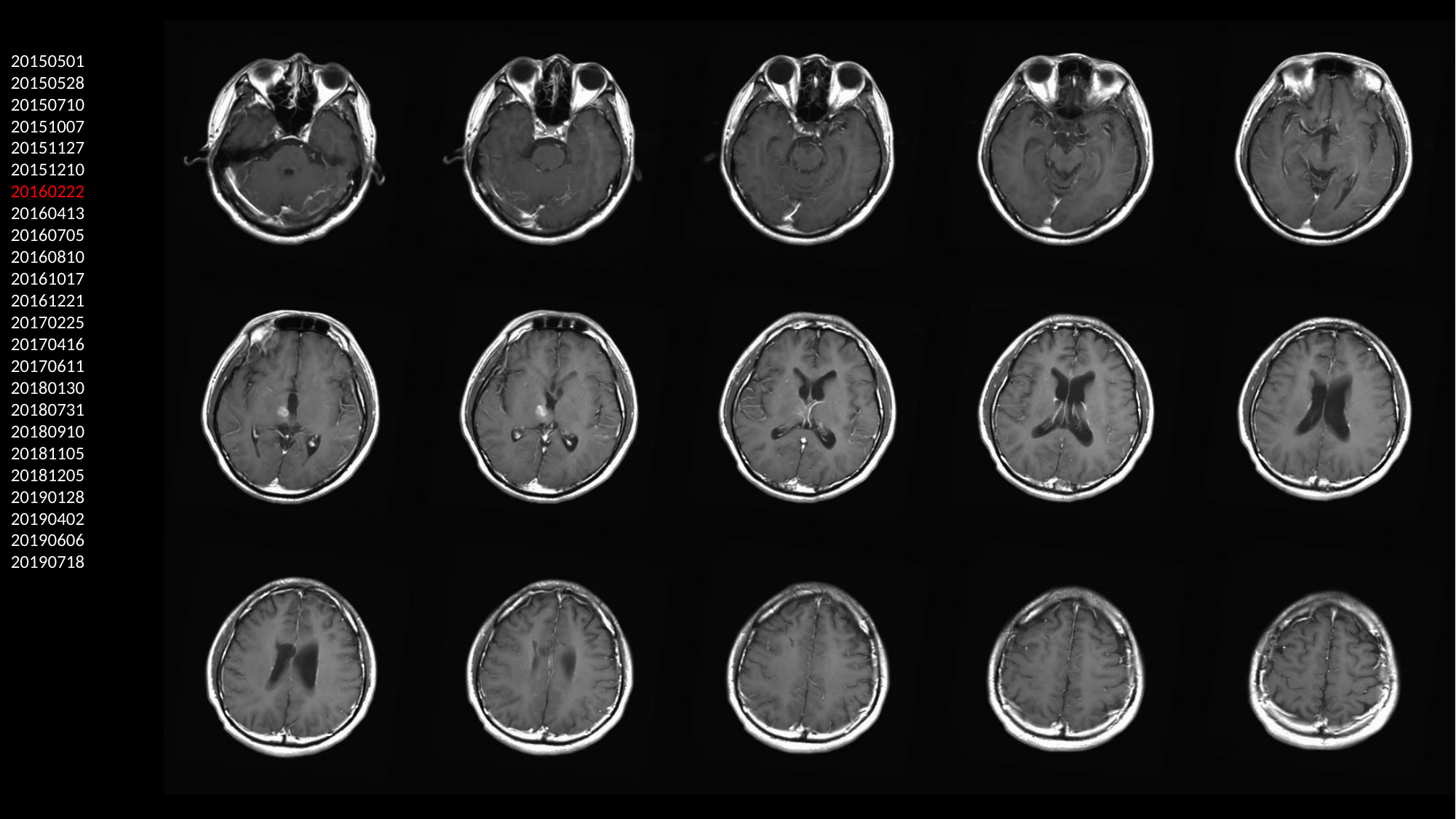

20150501
20150528
20150710
20151007
20151127
20151210
20160222
20160413
20160705
20160810
20161017
20161221
20170225
20170416
20170611
20180130
20180731
20180910
20181105
20181205
20190128
20190402
20190606
20190718

## Slide 39
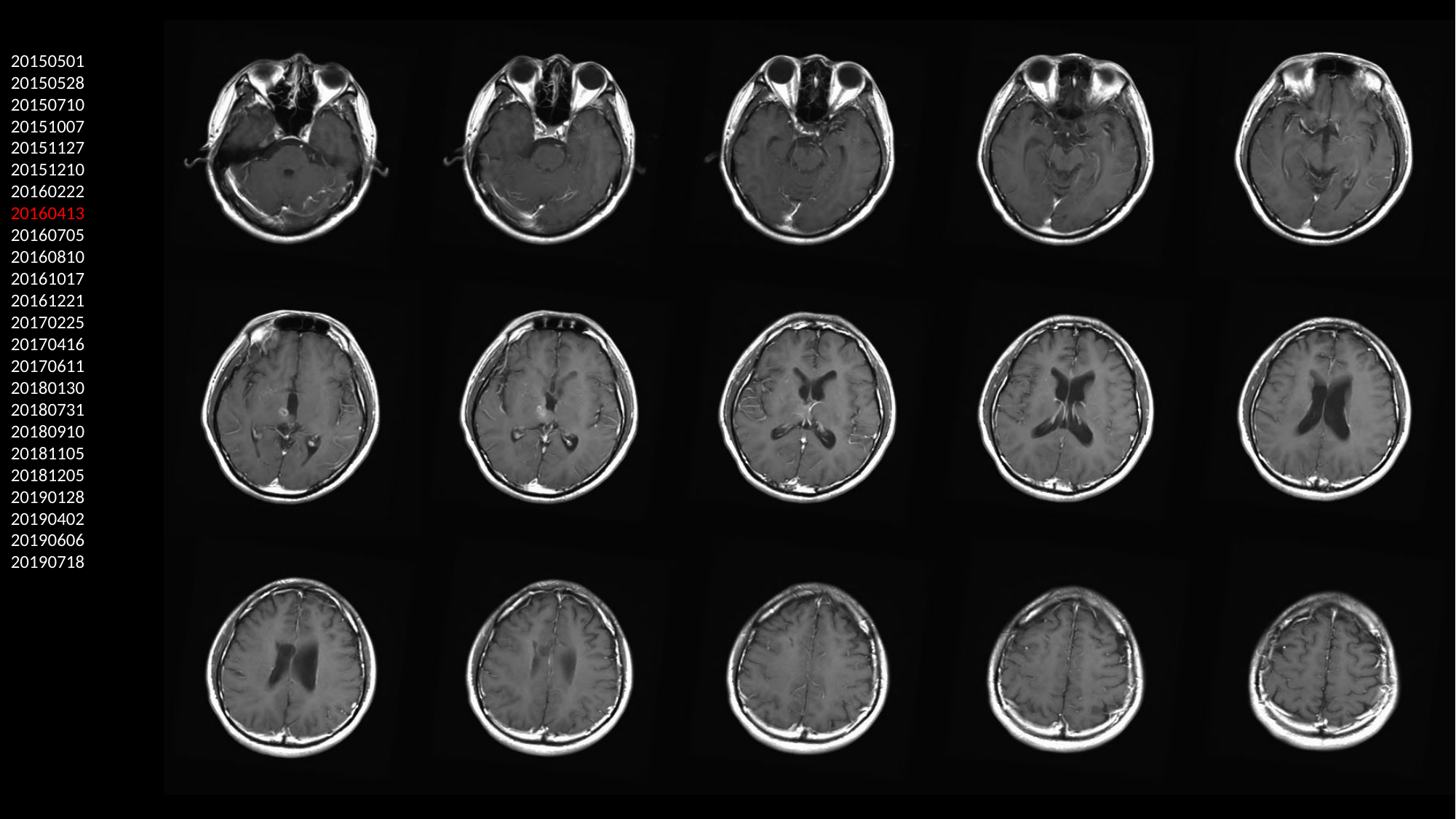

20150501
20150528
20150710
20151007
20151127
20151210
20160222
20160413
20160705
20160810
20161017
20161221
20170225
20170416
20170611
20180130
20180731
20180910
20181105
20181205
20190128
20190402
20190606
20190718

## Slide 40
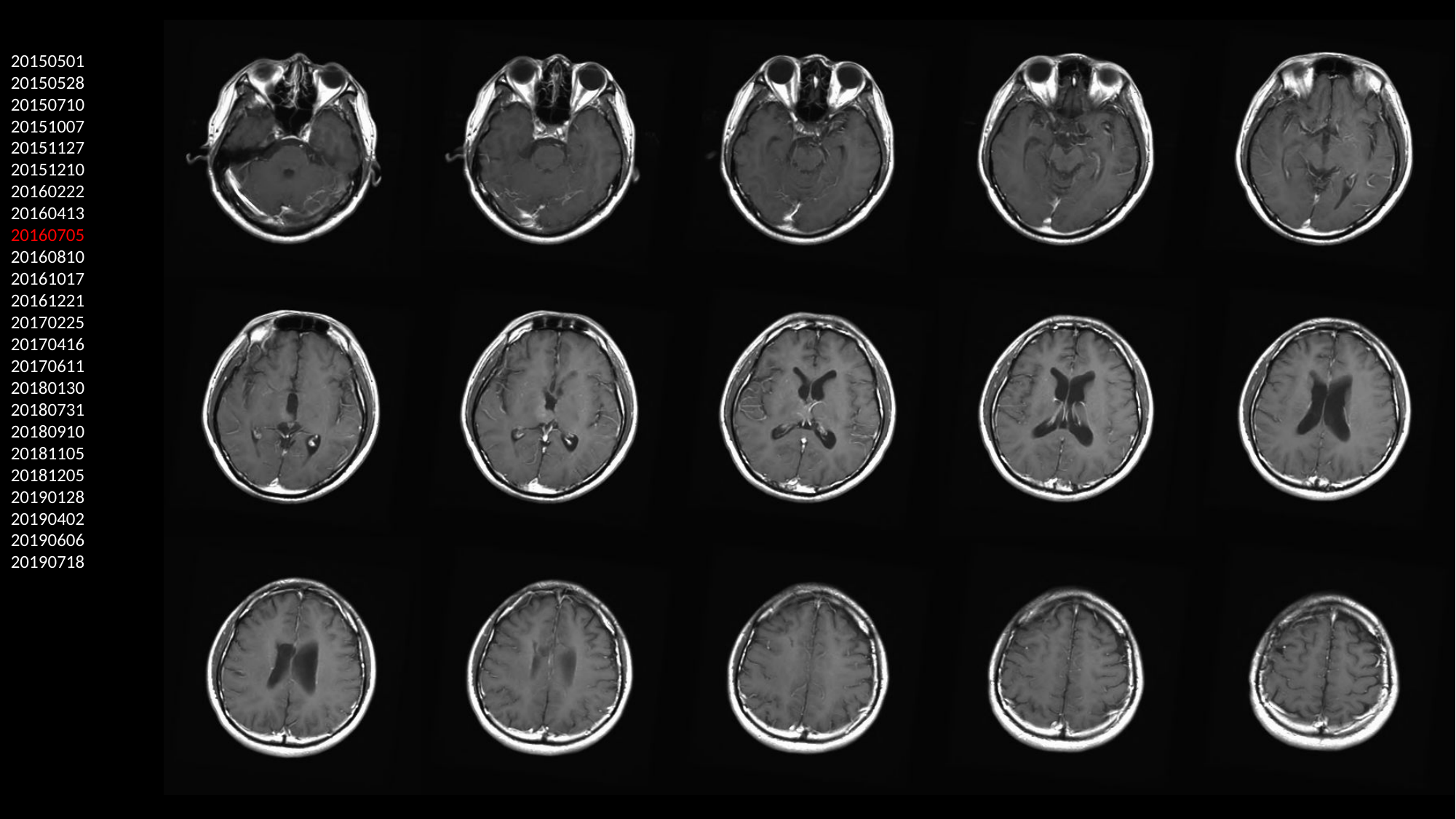

20150501
20150528
20150710
20151007
20151127
20151210
20160222
20160413
20160705
20160810
20161017
20161221
20170225
20170416
20170611
20180130
20180731
20180910
20181105
20181205
20190128
20190402
20190606
20190718

## Slide 41
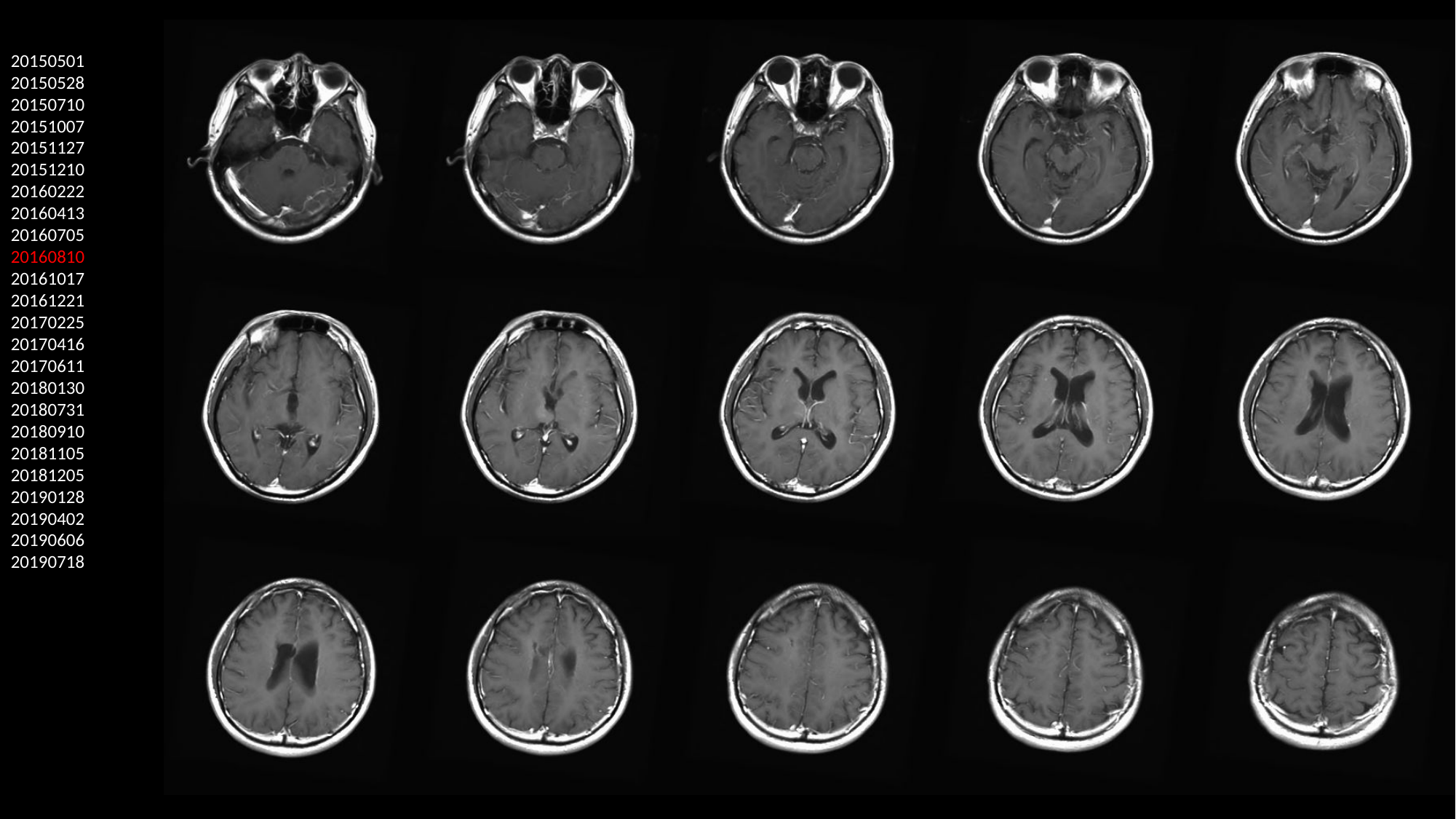

20150501
20150528
20150710
20151007
20151127
20151210
20160222
20160413
20160705
20160810
20161017
20161221
20170225
20170416
20170611
20180130
20180731
20180910
20181105
20181205
20190128
20190402
20190606
20190718

## Slide 42
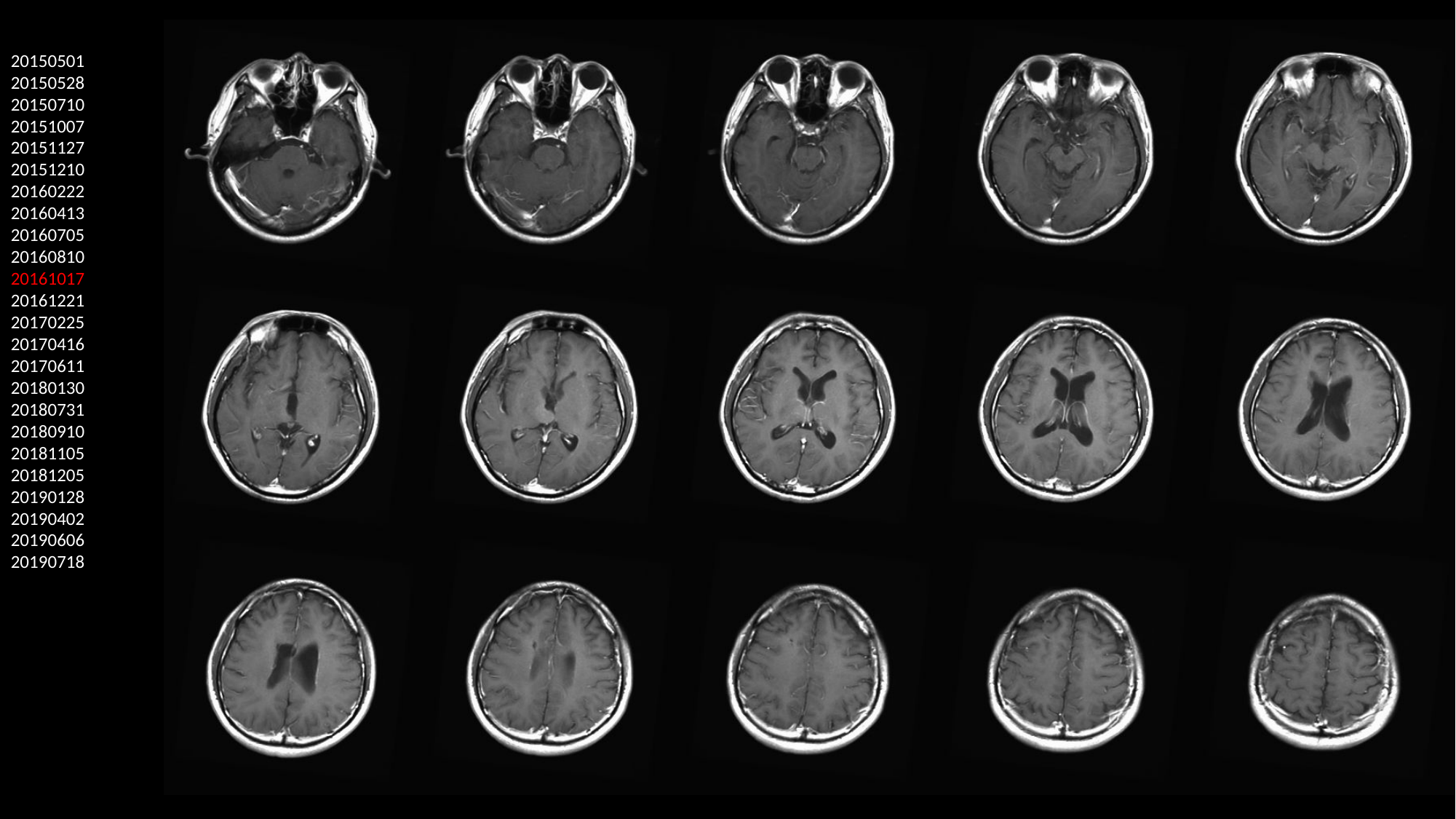

20150501
20150528
20150710
20151007
20151127
20151210
20160222
20160413
20160705
20160810
20161017
20161221
20170225
20170416
20170611
20180130
20180731
20180910
20181105
20181205
20190128
20190402
20190606
20190718

## Slide 43
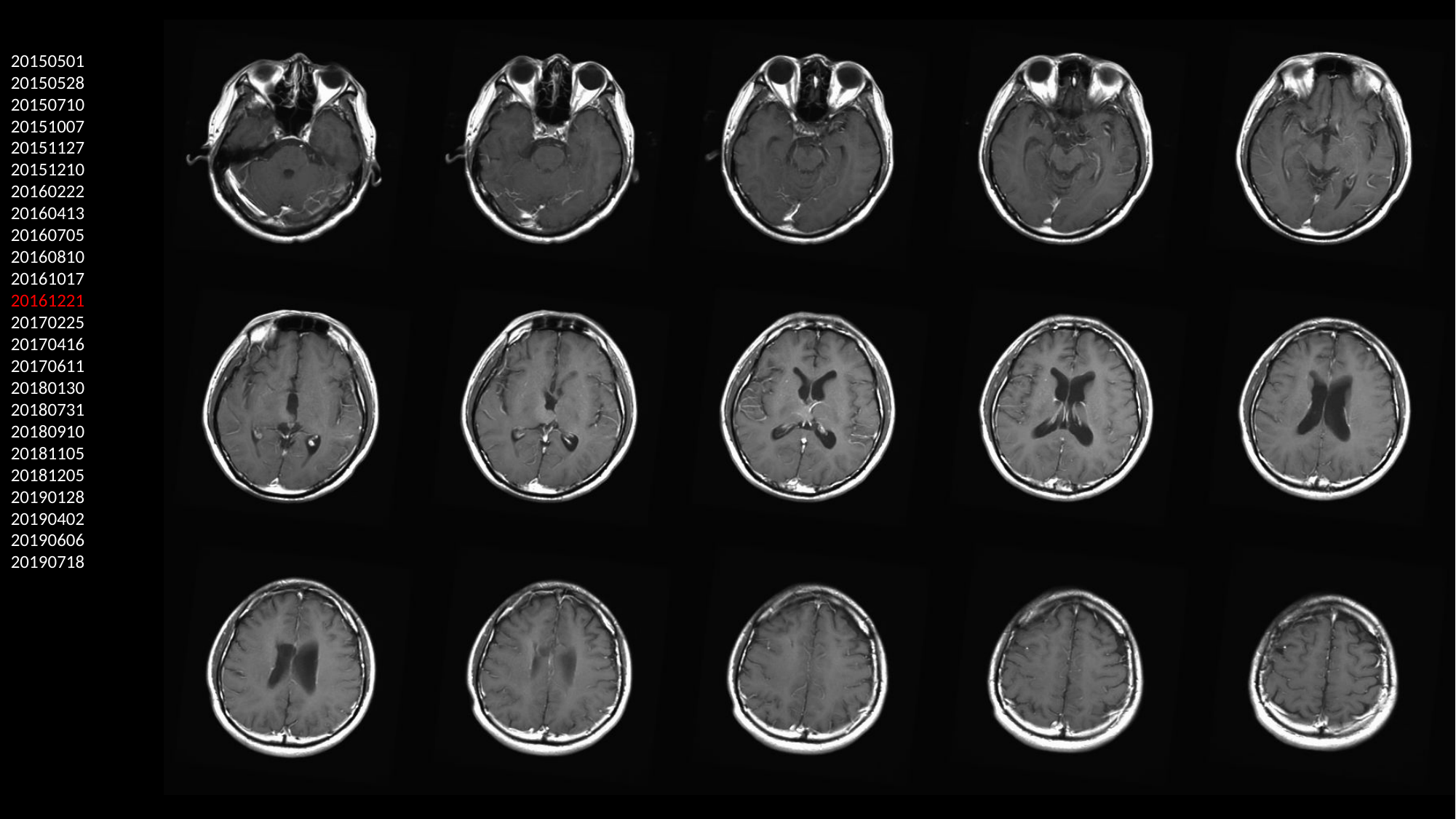

20150501
20150528
20150710
20151007
20151127
20151210
20160222
20160413
20160705
20160810
20161017
20161221
20170225
20170416
20170611
20180130
20180731
20180910
20181105
20181205
20190128
20190402
20190606
20190718

## Slide 44
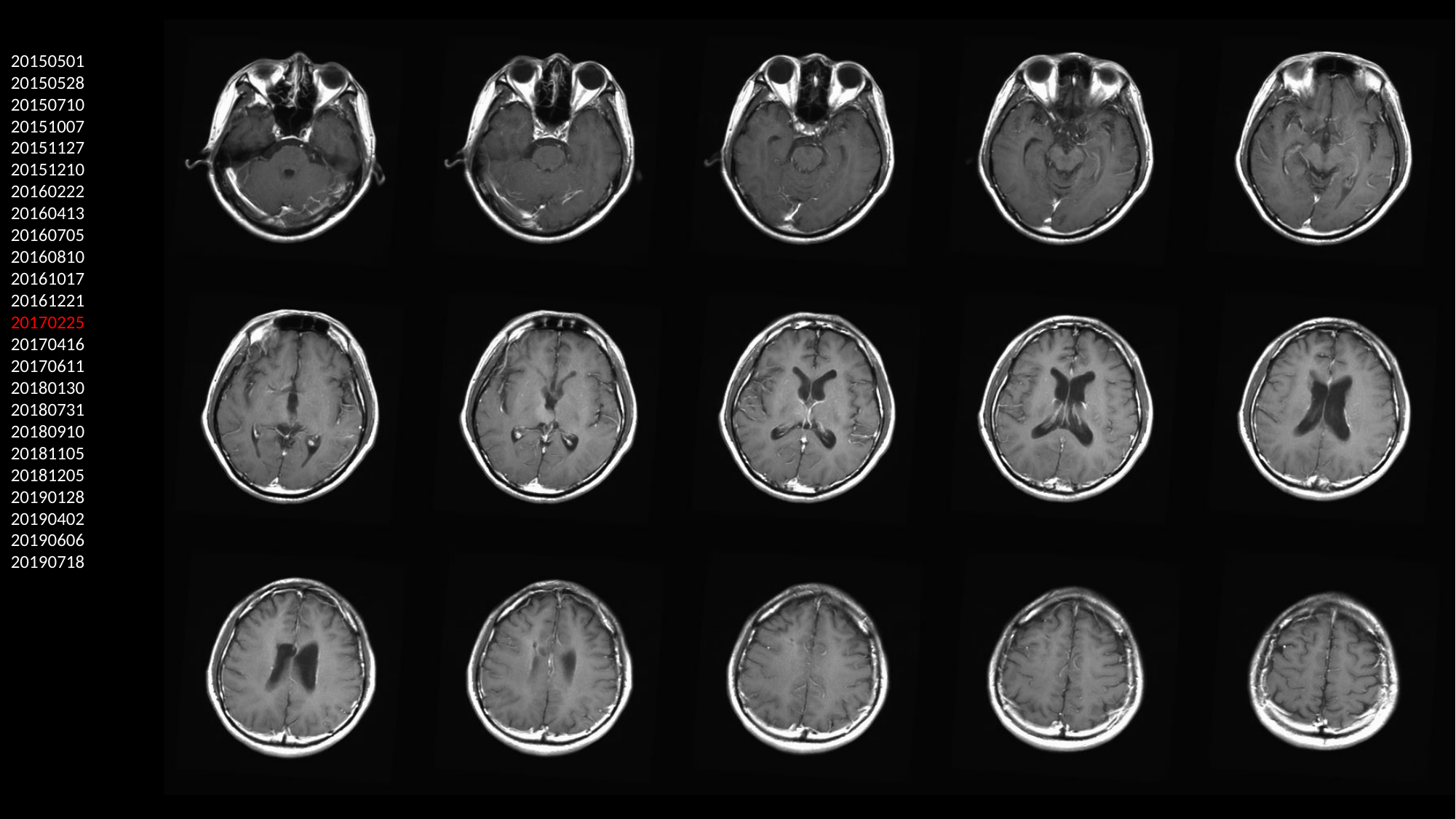

20150501
20150528
20150710
20151007
20151127
20151210
20160222
20160413
20160705
20160810
20161017
20161221
20170225
20170416
20170611
20180130
20180731
20180910
20181105
20181205
20190128
20190402
20190606
20190718

## Slide 45
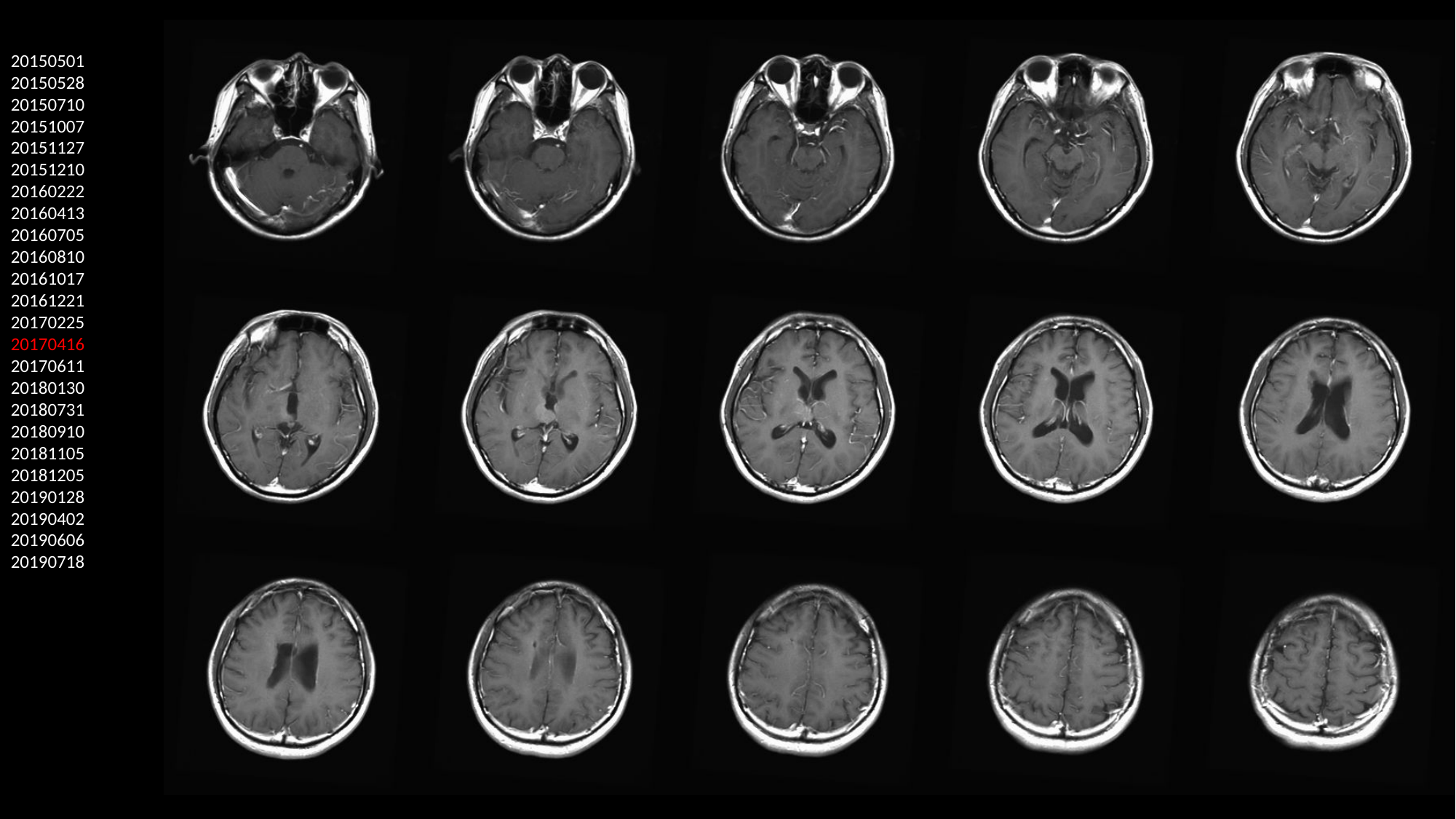

20150501
20150528
20150710
20151007
20151127
20151210
20160222
20160413
20160705
20160810
20161017
20161221
20170225
20170416
20170611
20180130
20180731
20180910
20181105
20181205
20190128
20190402
20190606
20190718

## Slide 46
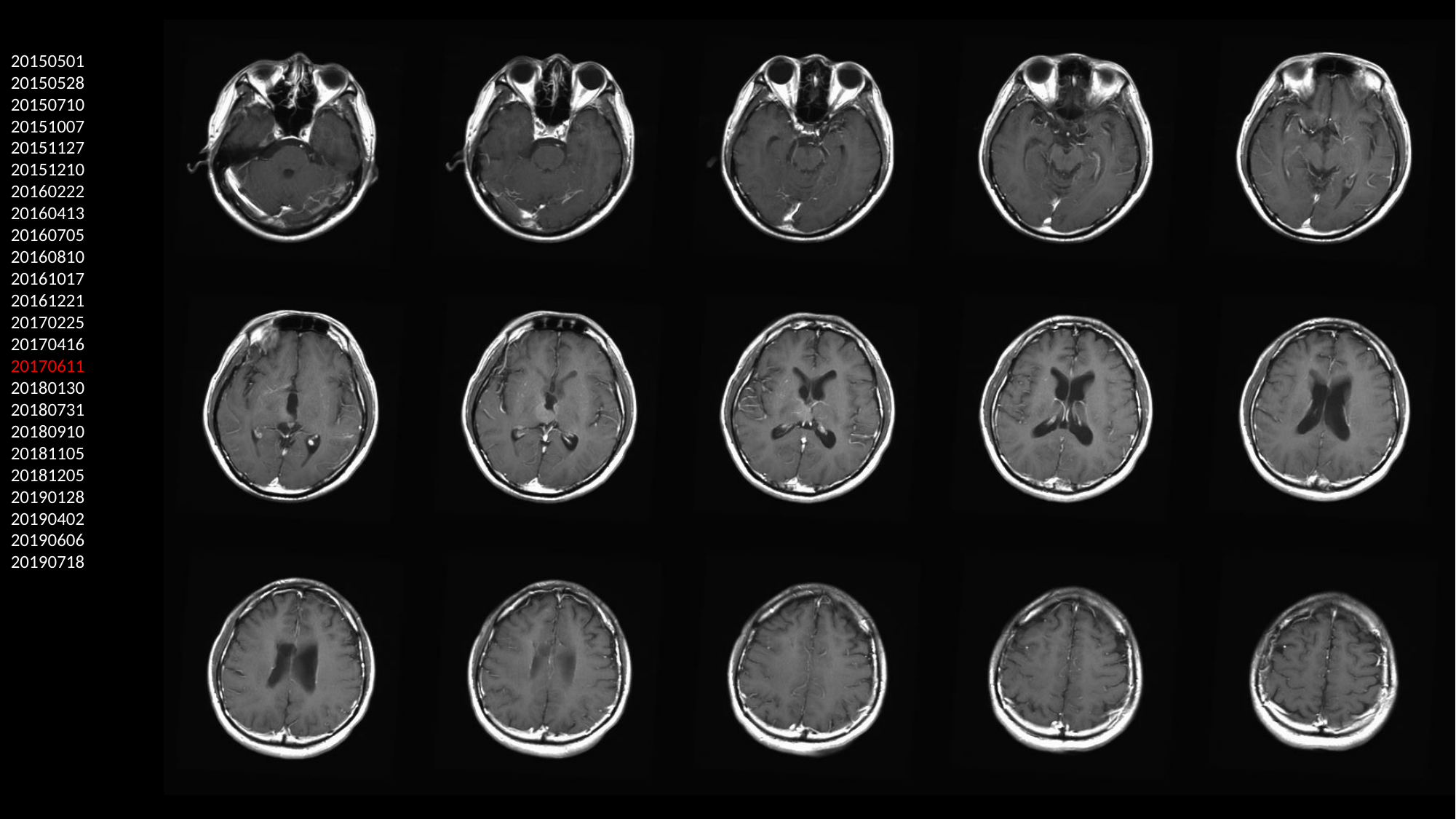

20150501
20150528
20150710
20151007
20151127
20151210
20160222
20160413
20160705
20160810
20161017
20161221
20170225
20170416
20170611
20180130
20180731
20180910
20181105
20181205
20190128
20190402
20190606
20190718

## Slide 47
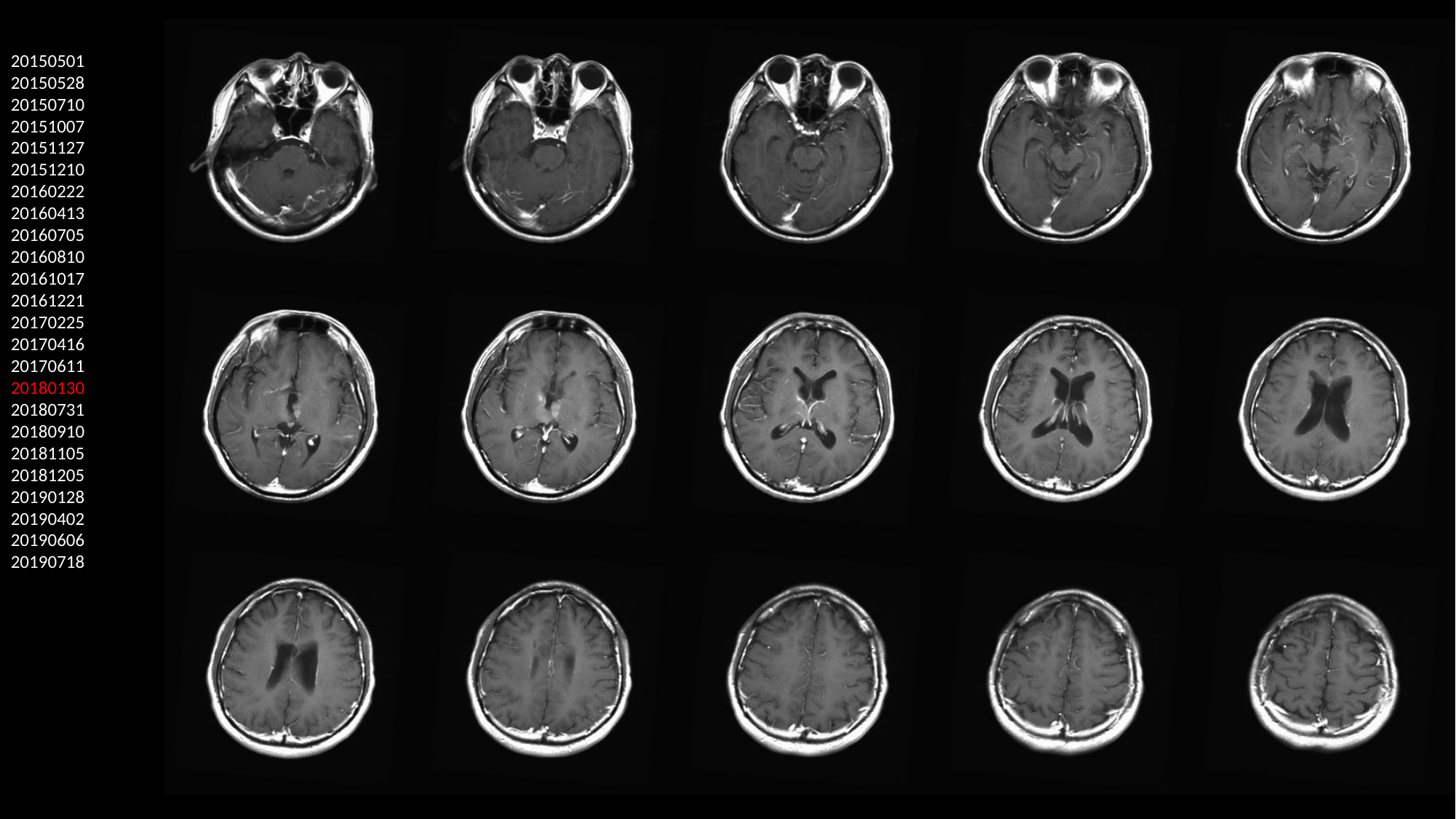

20150501
20150528
20150710
20151007
20151127
20151210
20160222
20160413
20160705
20160810
20161017
20161221
20170225
20170416
20170611
20180130
20180731
20180910
20181105
20181205
20190128
20190402
20190606
20190718

## Slide 48
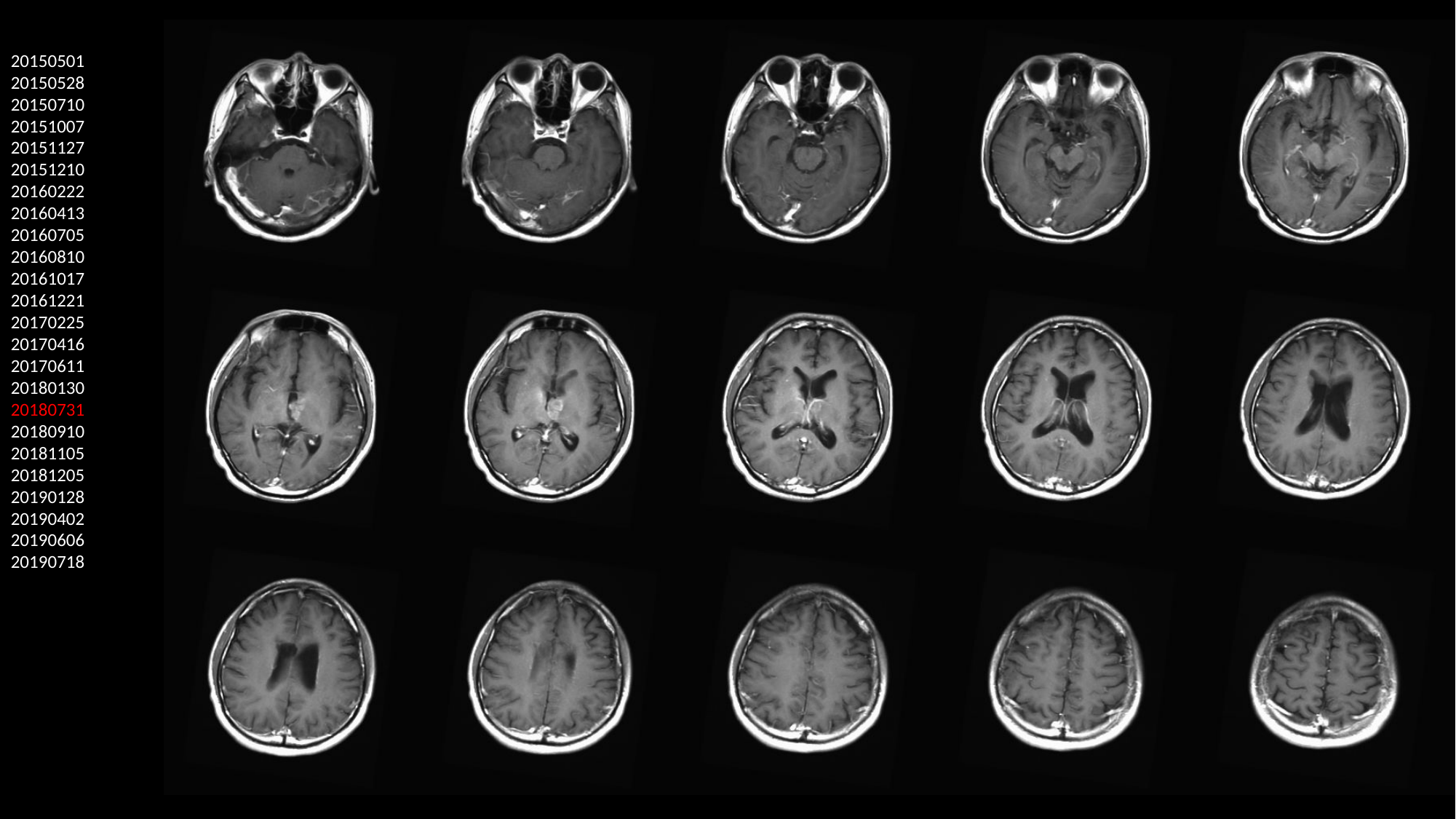

20150501
20150528
20150710
20151007
20151127
20151210
20160222
20160413
20160705
20160810
20161017
20161221
20170225
20170416
20170611
20180130
20180731
20180910
20181105
20181205
20190128
20190402
20190606
20190718

## Slide 49
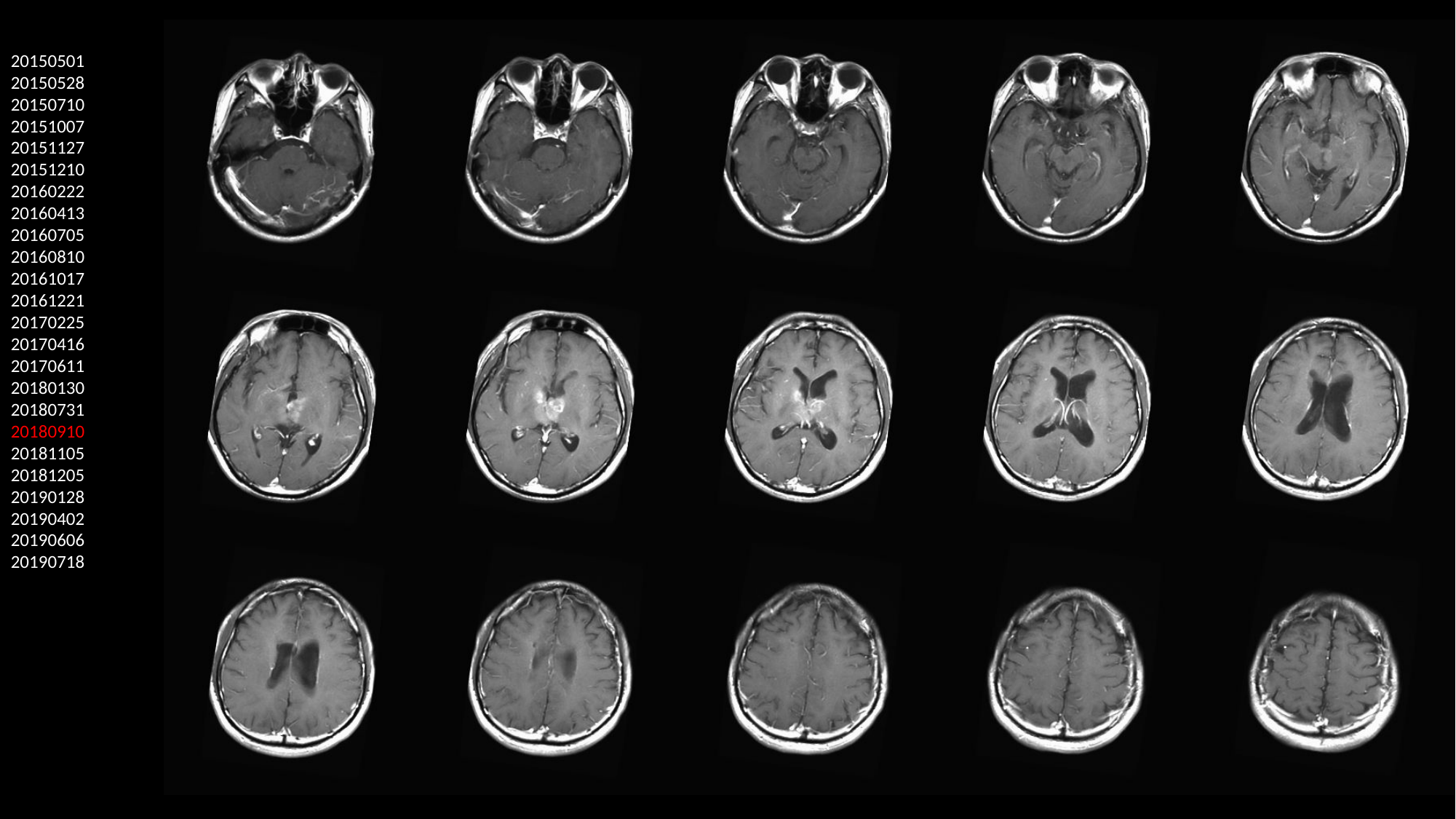

20150501
20150528
20150710
20151007
20151127
20151210
20160222
20160413
20160705
20160810
20161017
20161221
20170225
20170416
20170611
20180130
20180731
20180910
20181105
20181205
20190128
20190402
20190606
20190718

## Slide 50
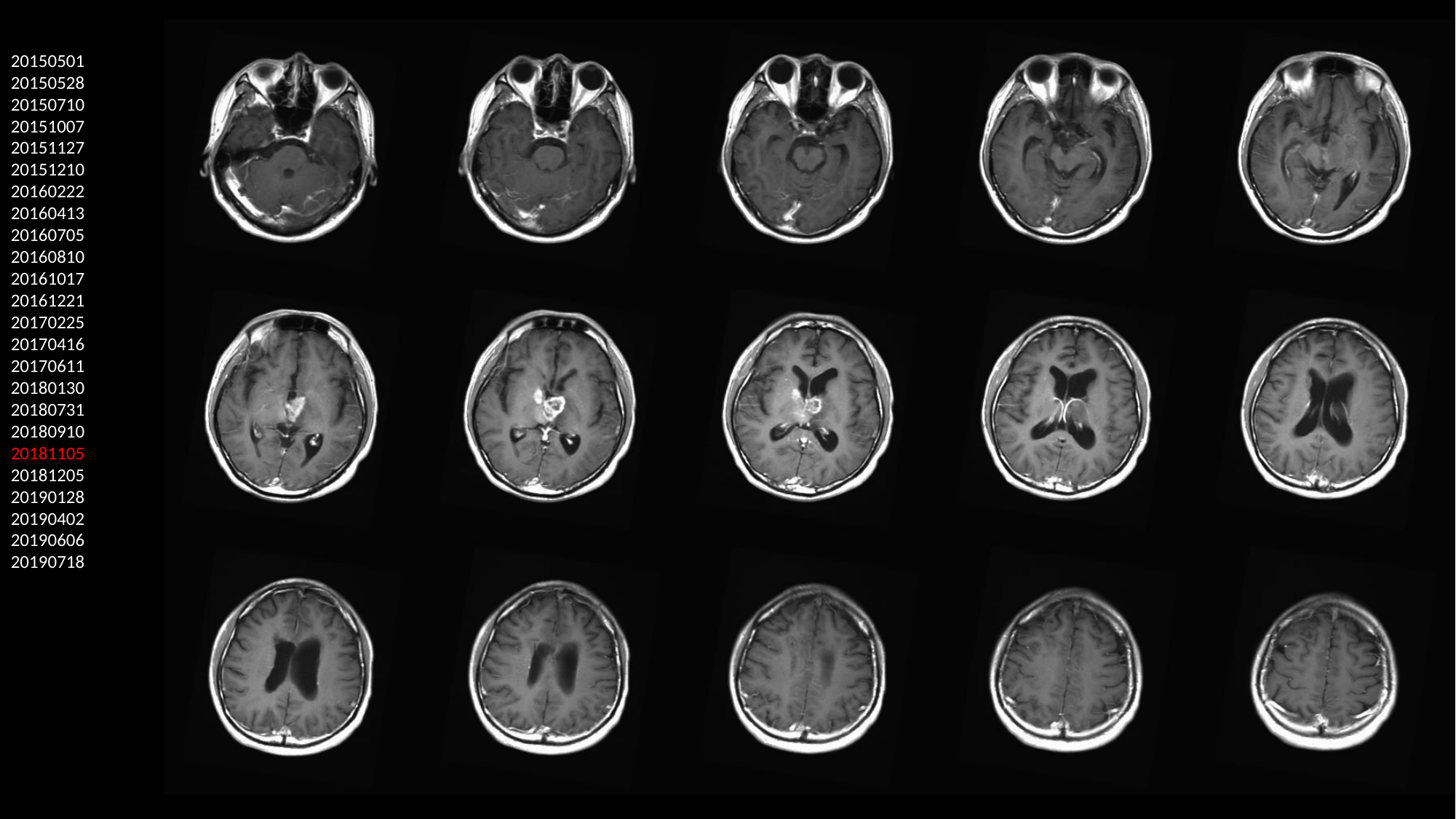

20150501
20150528
20150710
20151007
20151127
20151210
20160222
20160413
20160705
20160810
20161017
20161221
20170225
20170416
20170611
20180130
20180731
20180910
20181105
20181205
20190128
20190402
20190606
20190718

## Slide 51
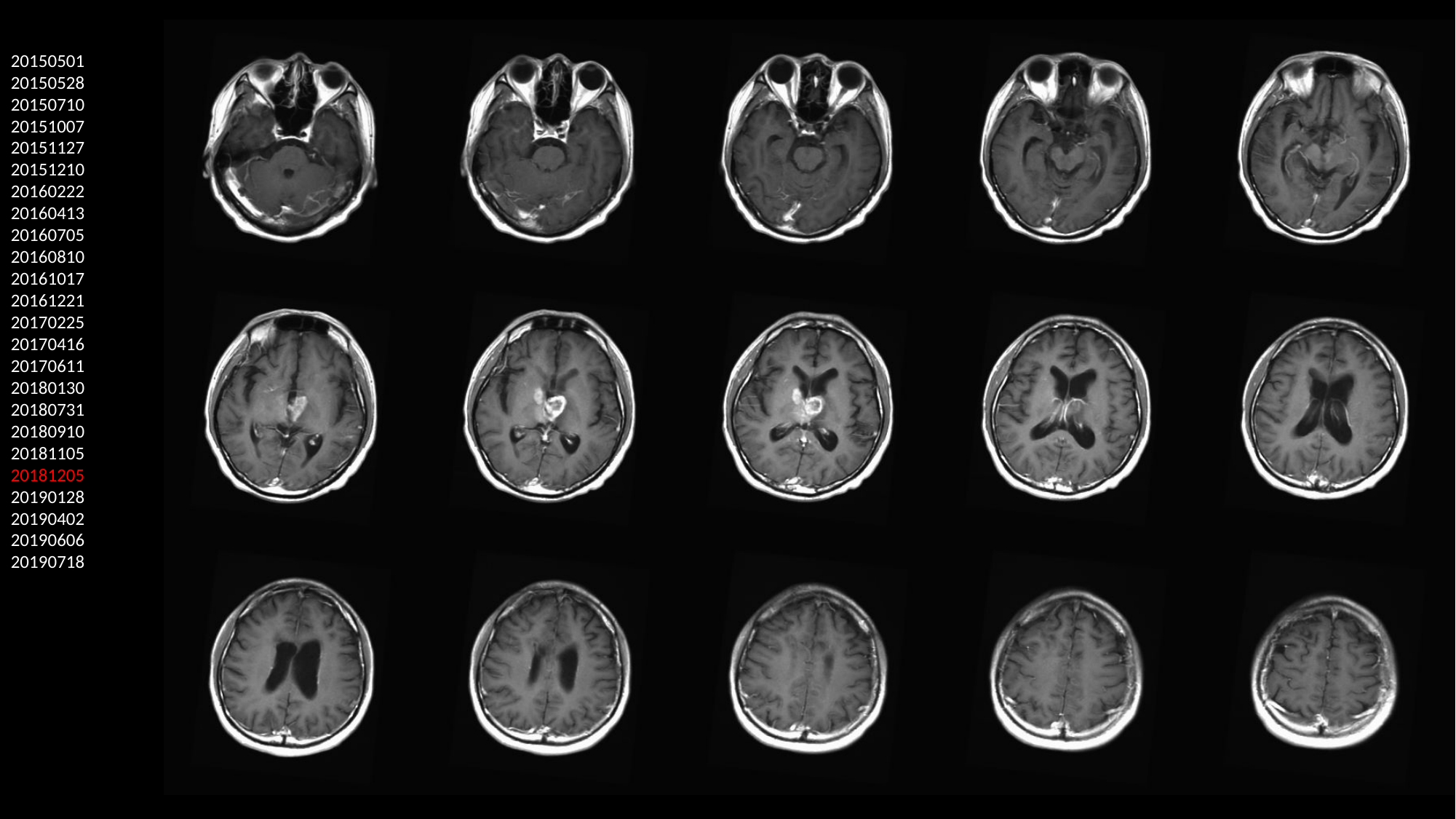

20150501
20150528
20150710
20151007
20151127
20151210
20160222
20160413
20160705
20160810
20161017
20161221
20170225
20170416
20170611
20180130
20180731
20180910
20181105
20181205
20190128
20190402
20190606
20190718

## Slide 52
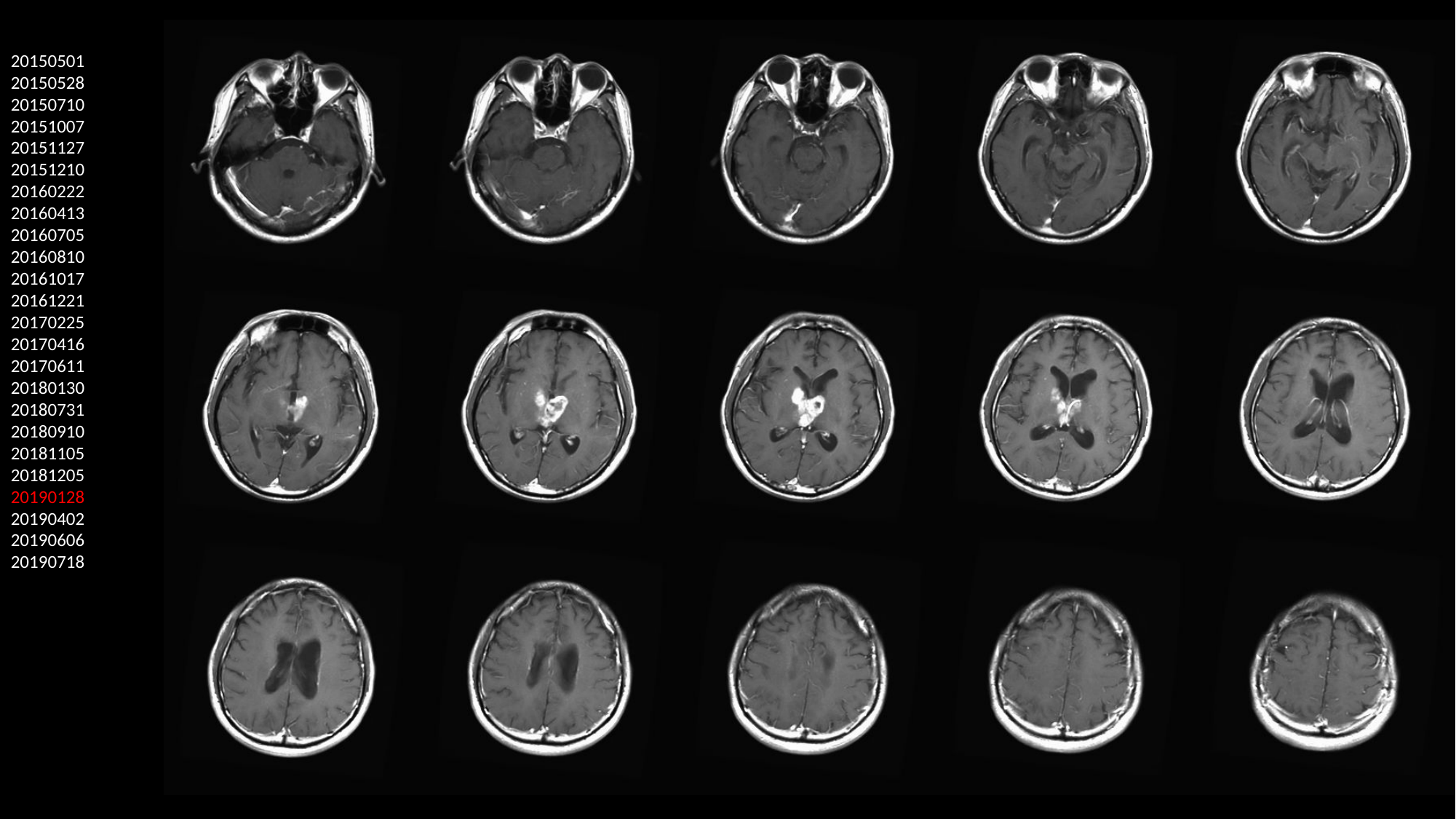

20150501
20150528
20150710
20151007
20151127
20151210
20160222
20160413
20160705
20160810
20161017
20161221
20170225
20170416
20170611
20180130
20180731
20180910
20181105
20181205
20190128
20190402
20190606
20190718

## Slide 53
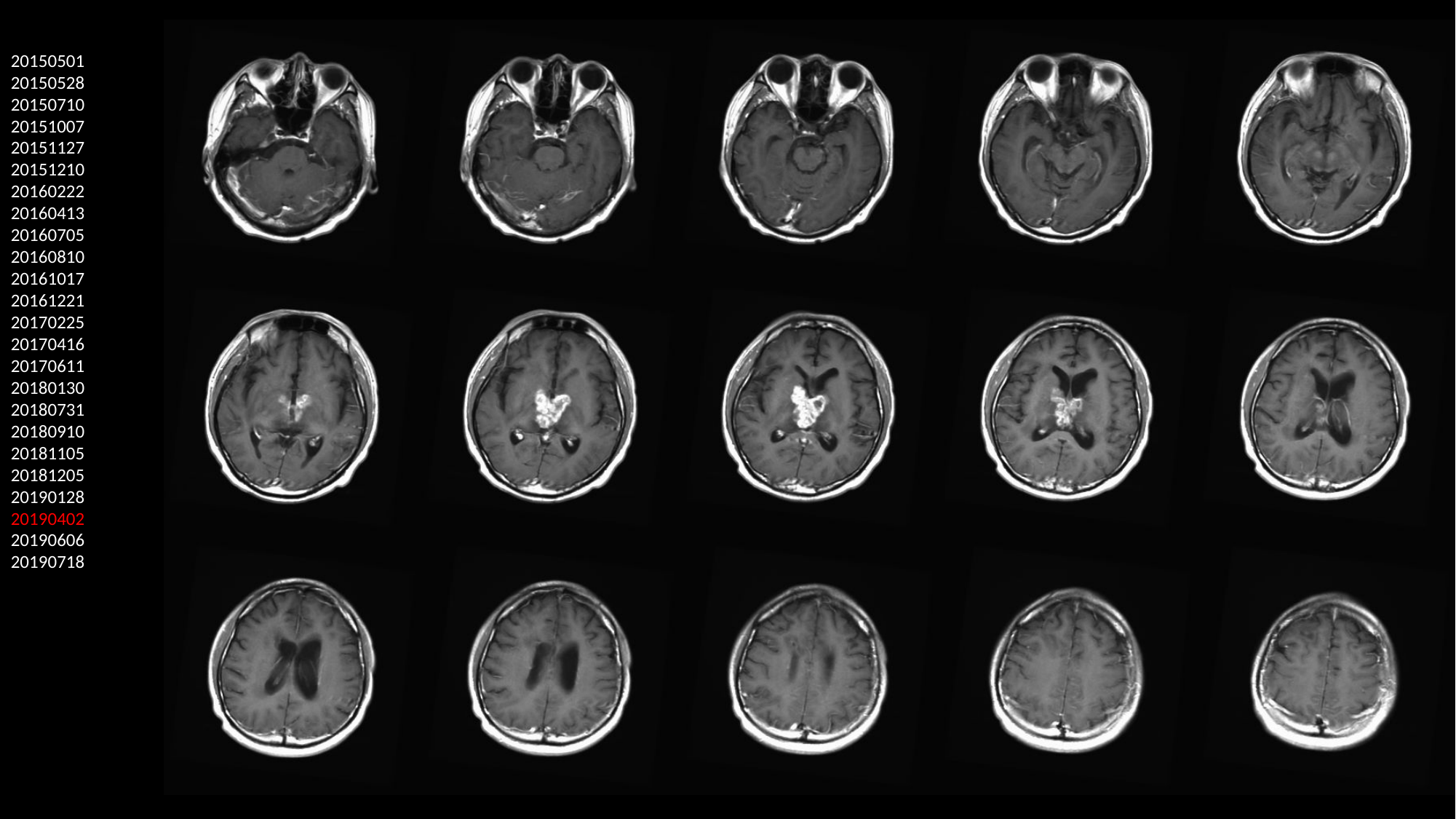

20150501
20150528
20150710
20151007
20151127
20151210
20160222
20160413
20160705
20160810
20161017
20161221
20170225
20170416
20170611
20180130
20180731
20180910
20181105
20181205
20190128
20190402
20190606
20190718

## Slide 54
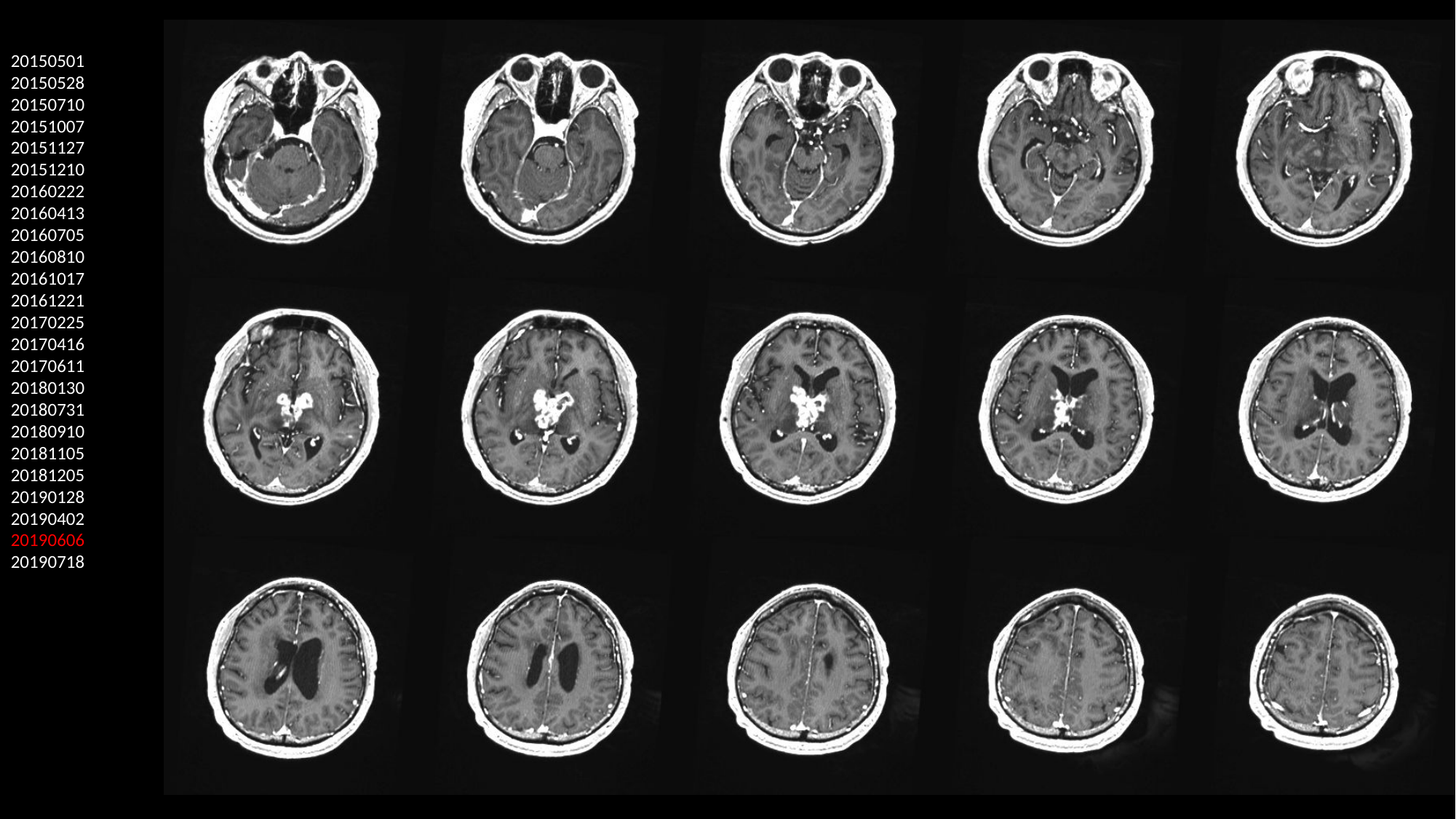

20150501
20150528
20150710
20151007
20151127
20151210
20160222
20160413
20160705
20160810
20161017
20161221
20170225
20170416
20170611
20180130
20180731
20180910
20181105
20181205
20190128
20190402
20190606
20190718

## Slide 55
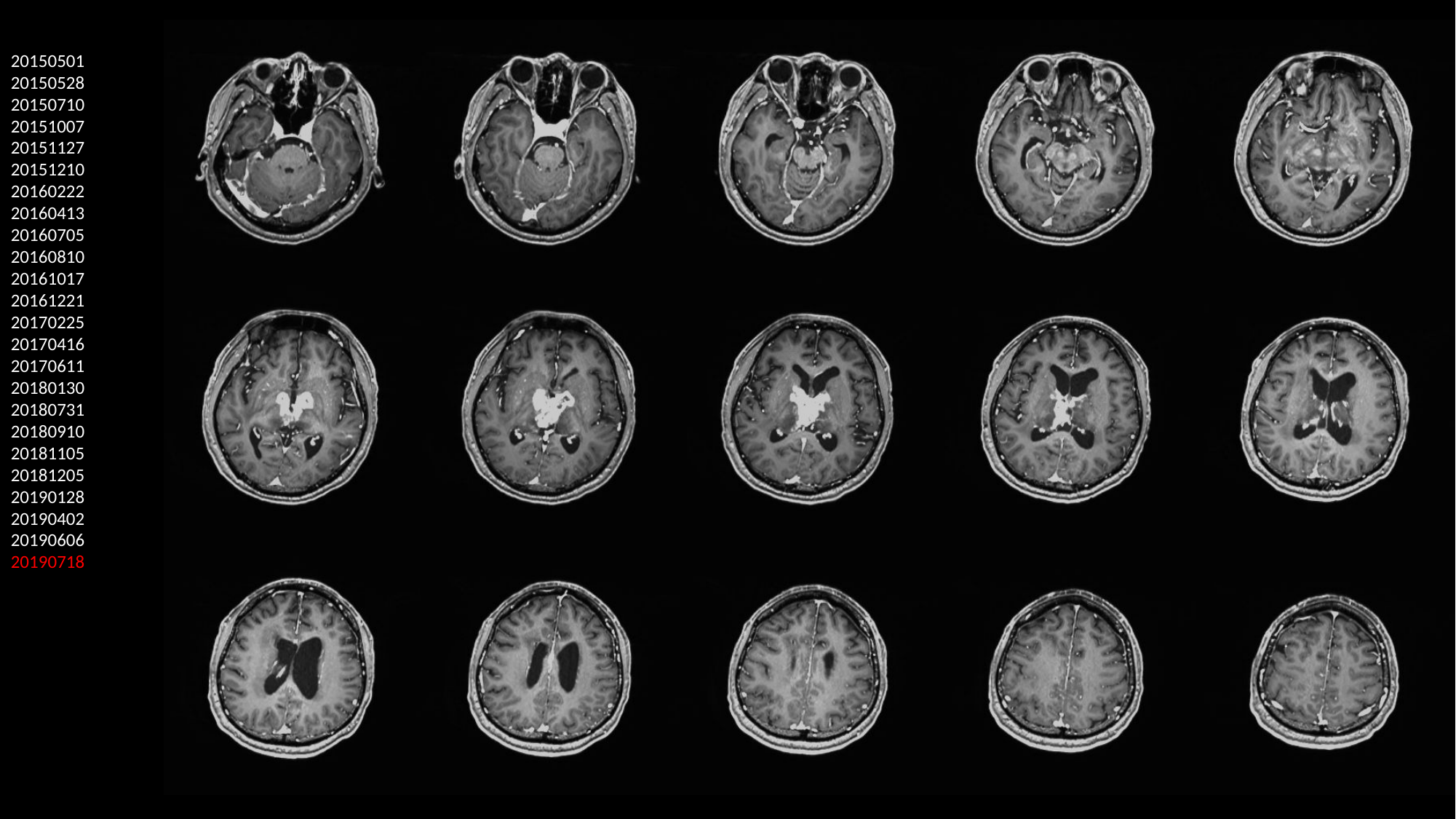

20150501
20150528
20150710
20151007
20151127
20151210
20160222
20160413
20160705
20160810
20161017
20161221
20170225
20170416
20170611
20180130
20180731
20180910
20181105
20181205
20190128
20190402
20190606
20190718
